# The cAMP responsive element modulator (CREM) transcription factor influences susceptibility to undernutrition and infection

**DOI:** 10.1128/mbio.01390-25

**Published:** 2025-06-27

**Authors:** Audrey C. Brown, Md. Jashim Uddin, Rebecca M. Munday, Farha Naz, G. Brett Moreau, Girija Ramakrishnan, Stephen S. Rich, Rashidul Haque, Genevieve L. Wojcik, Priya Duggal, Chelsea Marie, William A. Petri

**Affiliations:** 1 Division of Infectious Diseases and International Health, University of Virginia School of Medicine 214842 , Charlottesville, Virginia, USA; 2 Department of Epidemiology, Johns Hopkins Bloomberg School of Public Health 25802 , Baltimore, Maryland, USA; 3 Department of Genome Sciences, University of Virginia School of Medicine 12349, Charlottesville, Virginia, USA; 4 International Centre for Diarrheal Disease Research, Dhaka, Bangladesh; University of Geneva, Geneva, Switzerland

**Keywords:** undernutrition, malnutrition, genetic susceptibility, amebiasis, transcriptomics, metabolomics

## Abstract

We previously identified genetic polymorphisms associated with *Entamoeba histolytica*-positive diarrhea at the locus containing the transcription factor, cAMP responsive element modulator (*CREM*). Genetic association testing in birth cohorts of Bangladeshi children showed the previously described single nucleotide polymorphisms (SNPs) at the *CREM* locus associated with *E. histolytica-*positive diarrhea were independently associated with undernutrition at 1 year of age. The reference allele for both tested SNPs was associated with lower weight-for-age z-scores (WAZ) at 1 year of age (rs2148483: 0.161 reduction in WAZ per allele, *P* = 0.007; rs58000832: 0.203 reduction in WAZ, *P* = 0.001). Small intestinal transcriptome data from Bangladeshi and American children revealed differentially expressed genes (DEGs) were enriched for cAMP response element-binding sites. DEGs related to mitochondrial function were upregulated in the small intestine of children with the rs2148483 reference allele, while adaptive immune response processes were enriched among downregulated genes. Upregulation of mitochondrial function was mirrored by plasma metabolomics profiles concordant with a shift toward mitochondrial respiration. In mice, global conditional deletion of *Crem* resulted in reduced body weight, downregulation of genes related to innate immune function, and upregulation of metabolic gene expression in the intestine. Tissue-specific deletion of *Crem* in intestinal epithelial cells was sufficient to reduce overall body weight and alter intestinal expression of mitochondrial-related metabolic genes but not immune gene transcription. These data indicate that intestinal epithelial expression of *Crem* is linked to body weight, plausibly by regulation of mitochondrial gene expression, while *Crem* expression in other cell types influences the immune response to *E. histolytica*.

**IMPORTANCE:**

Undernutrition and diarrheal disease are leading causes of global childhood morbidity and mortality. Undernutrition can present as a cause or consequence of diarrheal diseases, leading us to hypothesize these phenotypes share a common genetic basis. Our identification of cAMP responsive element modulator (*CREM*) as a transcriptional regulator that influences susceptibility to both undernutrition and diarrheal disease in children growing up in an impoverished Bangladeshi community advances our understanding of the interaction of two major causes of childhood illness and offers the potential of therapy targeted to the cAMP-regulated transcription factor, *CREM*.

## INTRODUCTION

Globally, 148 million children under 5 are undernourished, and half of deaths among young children are linked to undernutrition (1). This is in part because undernourished children are more likely to become ill with infectious diarrheal diseases. Diarrheal disease caused by infectious agents remains a top cause of death in children under 5 (2, 3). *Entamoeba histolytica* is one such pathogen in which illness, termed amebiasis, causes diarrheal-associated morbidity and mortality in low- and middle-income countries (4–6). Children in Bangladesh who were underweight for age (weight-for-age Z-score [WAZ] of <−2) were more likely to become infected with *E. histolytica* (7, 8). Likewise, children with a history of amebiasis were 2.9 times (95% CI 1.01–8.52, *P* = 0.047) more likely to be undernourished. Undernutrition, as defined by being underweight (weight for age Z score of 2 standard deviations below the norm, WAZ < −2), reflects short-term undernutrition and tends to be reversible by accelerated growth following a period of growth faltering, whereas other metrics of undernutrition, such as stunting (height-for-age Z-score < −2), represent chronic undernourishment (9). Increased diarrheal infections are associated with reduced catch-up growth in children with low WAZ (10). As such, children with low WAZ in early life represent cases where nutritional or anti-pathogen intervention may have the greatest potential to mitigate the long-term impacts of undernourishment, such as shorter adult height, lower educational attainment, and reduced economic productivity (11).

Causal links between undernutrition and susceptibility to amebiasis remain poorly understood, but it is well appreciated that host genetics are a primary component of susceptibility to both infectious disease and metabolic programming (9, 12–14). A recent genome-wide association study (GWAS) of children enrolled in longitudinal cohorts in Bangladesh identified a novel host susceptibility locus for *E. histolytica-*positive diarrhea (12). Two single nucleotide polymorphisms (SNPs) reached genome-wide significance (*P* < 5 × 10^−8^) for association with *E. histolytica-*positive diarrhea, both in a single region on human chromosome 10p11.21. The susceptibility locus contained the genes *CUL2*, cAMP responsive element modulator (*CREM*), and *CCNY* in a region with strong linkage disequilibrium (LD) that prevented the determination of the putative causal SNP. Multiple lines of evidence, including data from the Genotype Tissue Expression consortium and global *CREM* deletion mice, indicated both SNPs affect *E. histolytica*-positive diarrhea via *CREM* (12). Together, these data present a strong association between *CREM* and increased *E. histolytica-*positive diarrhea susceptibility. Therefore, we refer to variants at rs2148483 and rs58000832 as “*CREM* locus” variants in this work, while it should be noted that neither SNP is located within the *CREM* gene itself. Other genetic variants in the *CREM* locus have been previously associated with inflammatory bowel disease, implying *CREM* is a key mediator of susceptibility to infectious and non-infectious colitis.

*CREM* is a transcription factor in the cAMP response element-binding protein superfamily, with numerous isoforms arising from multiple promoter sites and alternative splicing. Depending on the isoform exon configuration, *CREM* acts either as a transcriptional repressor or activator of target gene expression. Known roles for *CREM* include regulation of insulin sensitivity, cardiac hypertrophy, and spermatogenesis (15–19). *CREM* also functions in innate and adaptive immune responses. *CREM* regulates myeloid dendritic cells and T lymphocytes through transcriptional control of CD86 and IL-2/IL-17a, respectively (20, 21). *CREM* has described metabolic roles within both immune and non-immune tissue types (18, 19, 22–24). *CREM* regulates energy production in T lymphocytes through control of initial steps in both glutaminolysis and oxidative phosphorylation (23, 24). In non-immune tissue types, diminished *CREM* expression in visceral adipose tissue was associated with decreased insulin sensitivity in obese humans and mice (18); and *CREM*-dependent gene expression regulated the response to hyperglycemia in cardiomyocytes (22).

Here, we explore the role of *CREM* locus variants in susceptibility to undernutrition in children at 1 year of age. We report that in Bangladeshi infants living in an impoverished community in Dhaka, *CREM* locus variants associated with increased susceptibility to *E. histolytica* diarrhea were also associated with better nutrition (higher weight at 1 year of age). We explored potential mechanisms of *CREM* locus variants in susceptibility to undernutrition using small intestinal transcriptome and metabolome data from independent pediatric cohorts. Complementary studies using both global and intestinal epithelial cell-specific deletions of *Crem* in mice allowed us to map the site of body weight regulation via *Crem* to the intestinal epithelium plausibly by regulation of mitochondrial gene expression, while *CREM* expression in other cell types influenced the immune response to *E. histolytica*. Our work, by identifying a role for the transcription factor *CREM* in regulating body weight and metabolism, offers insight into the interrelatedness of nutrition and immunity.

## RESULTS

### *CREM* locus reference alleles were associated with reduced WAZ in Bangladeshi children

The alternate alleles at two SNPs in the *CREM* locus of human chromosome 10p11.21 were previously identified to be associated with increased susceptibility to *E. histolytica*-positive diarrhea (rs58000832, *P* = 6.1 × 10^−9^; rs2148483, *P* = 9.6 × 10^−8^) in infants growing up in an impoverished community in Dhaka (12). These SNPs were selected for investigation in the present study because they were of genome-wide significance (*P* < 5 × 10^−8^) for amebiasis (12). We investigated if body weight, measured as WAZ, was associated with these *CREM* locus SNPs using data from three prospective birth cohorts of Bangladeshi children: the Dhaka Birth Cohort (DBC), the Performance of Rotavirus and Oral Polio Vaccines in Developing Countries (PROVIDE) study, and the Cryptosporidiosis Burden Cohort (CBC) (Table 1). Major clinical differences across subjects in these cohorts were the percentage of undernourished children as defined by WAZ < −2 (*P* < 0.0001) and median WAZ (*P* < 0.0001) at 1 year of age, with more recent cohorts having fewer undernourished children. These differences reflect decreased undernutrition prevalence over time, consistent with global trends (1). There was no difference in the distribution of WAZ at 1 year by amebiasis history or sex in any cohort, indicating these factors were unlikely to confound genetic association testing for effects on WAZ (Fig. S1A and B).

**TABLE 1:** Characteristics of analyzed Bangladeshi prospective birth cohort participants^*a*^

Parameter	Dhaka birth cohort	PROVIDE	Crypto. burden cohort
*N*	312	432	366
Date of birth range	2008–2010	2011–2012	2014–2016
Female (%)	43.6%	45.8%	53.0%
Undernourished at 1 yr (%)*	35.9%	23.1%	18.6%
Median WAZ (SD)*	−1.55 (1.12)	−1.21 (1.14)	−1.08 (1.15)
rs2148483			
Alternate allele frequency	0.223	0.205	0.197
Observations (*N*)	287	421	349
rs58000832			
Alternate allele frequency	0.230	0.206	0.198
Observations (*N*)	258	374	310

^*a*^ Undernourished is defined as WAZ < −2. Alternate allele frequencies represent that of each cohort. Asterisks indicate rows with significant differences across cohorts determined by analysis of variance (*P* < 0.0001).

We hypothesized that the *CREM locus* SNPs could influence both amebic diarrhea and nutrition at 1 year of age (12). In fact, the reference alleles at both rs2148483 (G > A) and rs58000832 (C > CA) (associated with resistance to *E. histolytica* diarrhea) were significantly associated (*P* < 0.05) with decreased WAZ in a meta-analysis combining the three cohorts (rs2148483: (β_META_ = −0.161, *P*_META_ = 0.007, *P*_HET_ > 0.05); rs58000832: (β_META_ = −0.203, *P*_META_ = 0.001, *P*_HET_ > 0.05); Table 2). The reference alleles at rs2148483 (G) and rs58000832 (C) conferred an estimated 0.161 and 0.203 decrease in WAZ at 1 year of age, respectively, per copy of allele. We next modified WAZ values using this combined set of children to simulate how frequency of undernutrition would be affected if all subjects were homozygous for the alternate allele at rs58000832, which would confer an estimated 0.203 and 0.406 increase in WAZ to children heterozygous or homozygous for the rs58000832 reference allele, respectively. Prior to modification, 25.5% of all children were undernourished (WAZ < −2); this decreased by one-third to 17.0% after simulation of the effect of rs58000832 homozygous reference allele genotype.

**TABLE 2:** Association of *CREM* locus alleles with WAZ at 1 year of age^*a*^

rsID	Position (GRCh37)	Reference allele	Alternate allele	DBC		PROVIDE		CBC		Meta-analysis		
				Estimate [95% CI]	*P*	Estimate [95% CI]	*P*	Estimate [95% CI]	*P*	Estimate	*P*	*P*_het_
rs2148483	35341301	G	A*	−0.178 [−0.390–0.033]	0.099	−0.204 [−0.393–0.014]	**0.036**	−0.088 [−0.300, 0.124]	0.416	−0.161	**0.007**	0.713
rs58000832	35517636	C	CA*	−0.226 [−0.449–0.001]	**0.049**	−0.231 [−0.435–0.027]	**0.027**	−0.147 [−0.369-0.075]	0.197	−0.203	**0.001**	0.836

^*a*^ rsIDs indicate a unique label for an SNP mapped to a specific genomic location. Estimate (beta) equals the average predicted decrease in WAZ for each copy of reference allele harbored by an individual, adjusted for genetic principal components, sequencing batch (DBC), and history of *E*. *histolytica*-associated diarrhea (DBC and PROVIDE). Asterisk indicates the risk allele for *E. histolytica*-associated diarrhea identified by GWAS. Significant findings (*P* < 0.05) are indicated in bold.

In a study-stratified analysis, the reference allele at rs2148483 was associated with WAZ in PROVIDE alone (β_PROVIDE_ = −0.204, 95% CI_PROVIDE_ = −0.393–0.014, *P*_PROVIDE_ = 0.036) but not for either DBC or CBC (β_DBC_ = −0.178, 95% CI_DBC_ = −0.390–0.033, *P*_DBC_ = 0.099; β_CBC_ = −0.088, 95% CI_CBC_ = −0.300–0.124, *P*_CBC_ = 0.161) (Table 2). The reference allele at rs58000832 was associated with WAZ in both DBC and PROVIDE (β_DBC_ = −0.226, 95% CI_DBC_ = −0.449–0.001, *P*_DBC_ = 0.049; β_PROVIDE_ = −0.231, 95% CI_PROVIDE_ = −0.435–0.027, *P*_PROVIDE_ = 0.027) while CBC did not reach statistical significance (β_CBC_ = −0.147, 95% CI_CBC_ = −0.369–0.075, *P*_CBC_ = 0.197) (Table 2), but the effect was consistent in direction. Estimates (beta) from genetic association testing in all cohorts for both SNPs trended toward decreased WAZ at 1 year of age with copies of the reference allele (rs2148483 [G], rs58000832 [C]) (Table 2). For each SNP site, unadjusted median WAZ values at 1 year of age were non-significantly lower in children homozygous for the given reference allele relative to children with copies of the alternate allele, with the exception of children homozygous for either alternate allele in DBC and PROVIDE (Fig. S1C and D). This opposing effect, where a genetic factor increases risk for one phenotype while being protective against another, contrasts with the observed environmental synergy commonly seen between undernutrition and diarrheal disease. We concluded that the reference alleles at both rs2148483 (G > A) and rs58000832 were significantly associated with resistance to amebic diarrhea and susceptibility to undernutrition.

### Reference alleles at the *CREM* locus were associated with increased metabolic and decreased immune pathway transcription

*CREM* is a transcription factor capable of influencing gene expression across broad processes by binding at cAMP response elements in the promoter regions of target genes. Our goal was to investigate how *CREM* locus variants influence *E. histolytica*-positive diarrhea susceptibility and WAZ by identifying *CREM*-dependent intestinal expression differences between genotypes at *CREM* locus SNPs. As the small intestine is the site of nutrient absorption, we posited that *CREM* may affect WAZ by transcriptional regulation of nutrient transporters. Transcriptome data were analyzed from duodenal biopsies of 199 individuals across three groups: American children (*N* = 81), Bangladeshi children (*N* = 37), and Bangladeshi adults (*N* = 81) (Table S1) (25). These children were genotyped for rs2148483, while genotyping of rs58000832 failed, likely due to the repetitive nature of the polyA track where this indel sits.

**Fig 1:**
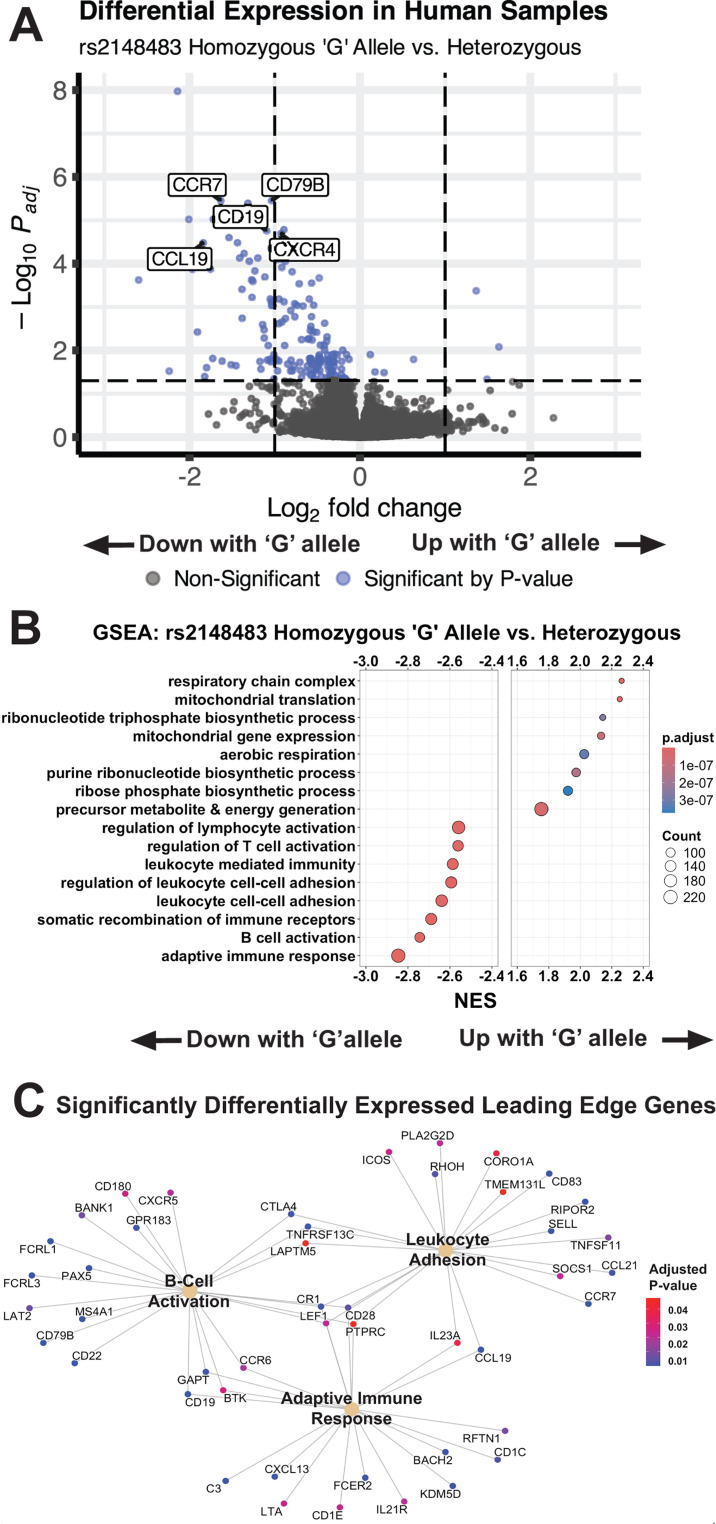
RNAseq of human small intestinal biopsies reveals altered immune and metabolic transcription. (**A**) Volcano plot of differentially expressed genes by rs2148483 genotype. (**B**) Gene set enrichment analysis using biological process gene ontology (GO) terms. (**C**) Significantly differentially expressed genes contributing to the leading edge of the three most downregulated pathways by normalized enrichment score (NES). Gene nodes colored by adjusted *P*-value from DESeq2 results; all genes displayed had negative log2 fold change.

Transcriptome data from children with two copies of the rs2148483 reference (G) allele (*N* = 130) were compared to heterozygotes (*N* = 59). Data from individuals homozygous for the alternate (A) allele were not included due to low sample size (*N* = 10). There were 172 genes differentially expressed between rs2148483 homozygous reference (G) allele and heterozygous individuals using an adjusted *P*-value threshold of <0.05 (Fig. 1A; File S1). Of these genes, 7 were upregulated while 165 were downregulated. The 172 differentially expressed genes (DEGs) were compared to transcriptomic results stratified by study site. One hundred seventy of 172 genes showed the same directionality of log2 fold change when compared to the combined analysis for both American (*N* = 81) and Bangladeshi (*N* = 118) stratified analyses (File S1). One hundred twenty of 172 DEGs were associated with a predicted cAMP response element-binding site, indicating a high percentage of plausible *CREM* target genes in our data set (File S1) (26). In the combined analysis, the homozygous reference (G) genotype was associated with a reduction in several immune-related genes. Genes involved in B-cell activation (*CD19, CD79B,* and *CD22*) and lymphocyte migration (*CCR7, CXCR4,* and *CCL19*) were downregulated in individuals with two copies of the reference allele. Differential expression was not observed for genes encoding nutrient transporters, such as *SLC5A1* and *SLC15A1*, which encode glucose transporter SGLT1 and peptide transporter PEPT1, respectively.

Published data suggested the alternate alleles of both *CREM* locus SNPs of interest reduced gene expression (12). Despite identifying expression changes in genes with predicted cAMP response element-binding sites, we found no difference in *CREM* overall expression level or isoform usage by rs2148483 genotype in human duodenal samples (File S1; Fig. S2A and B). Expression of neighboring genes residing in the same block of linkage disequilibrium, *CUL2* and *CCNY*, was also not significantly affected (File S1; Fig. S2C and D).

To further determine which processes were associated with the *CREM* rs2148483 reference allele, gene set enrichment analysis (GSEA) using biological process gene ontology (GO) terms was performed on full DESeq2 results arranged by Wald statistic. Downregulated genes were enriched for processes related to immune cell function, including the adaptive immune response, B cell activation, and leukocyte cell-cell adhesion (Fig. 1B and C; File S1). Several processes related to metabolism were associated with increased gene expression in individuals homozygous for the rs2148483 reference allele, particularly those related to mitochondrial respiration and metabolite catabolism (Fig. 1B). The highest-ranking genes contributing to the enrichment of metabolic gene sets (Fig. 1B) include mitochondrial RNA polymerase (*POLRMT*), subunits of the NADH-ubiquinone oxidoreductase complex (*NDUFV3, NDUFV1, NDUFB11,* and *NDUFB10*), and mitochondrial ribosomal proteins (*MRPL54, MRPS34, MRPL52,* and *MRPS5*). We concluded that the *CREM* rs2148483 reference allele was associated with increased mRNA expression of metabolic genes and decreased expression of immune genes.

### Reference alleles at the *CREM* locus were associated with decreased metabolite levels

**Fig 2:**
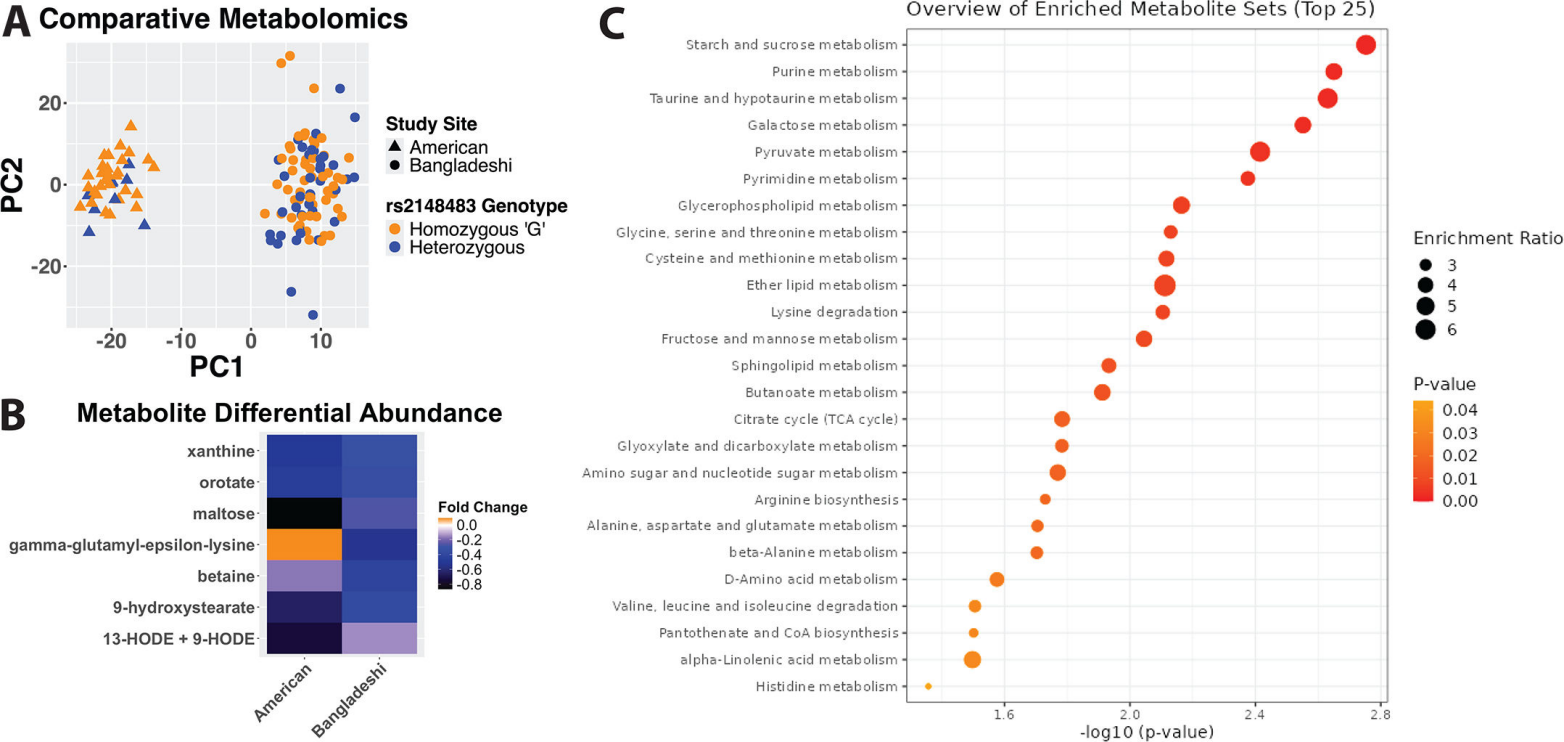
*CREM* locus polymorphism is associated with altered human plasma metabolome. (**A**) Principal component analysis of all metabolites detected in at least 80% of samples. (**B**) Individual metabolites with FDR < 0.1 for differential abundance by rs2148483 genotype in a combined analysis of both study sites. Negative fold change is associated with reduction in homozygous “G” allele compared to heterozygotes. (**C**) Metabolite set enrichment analysis to identify altered metabolic pathways by rs2148483 genotype.

Perturbations in metabolic gene expression were identified across multiple processes (Fig. 1B) that suggested *CREM* locus variants influence the metabolome via transcriptional regulation of aerobic respiration genes. An analysis of untargeted metabolomics data were conducted on plasma samples collected from 139 children (42 American children included in Table S1) and 97 Bangladeshi children from CBC (included in Table 1; Fig. 2A). Participants were genotyped for rs2148483, and metabolite profiles were compared between those homozygous for the reference (G) allele and heterozygous individuals. Seven hundred nineteen metabolites were included in the analysis after filtering for metabolites present above the limit of detection in at least 80% of samples. While study site was the most impactful factor separating subjects (Fig. 2A), metabolite profiles were also significantly different between rs2148483 genotypes by permutation multivariate analysis of variance (PERMANOVA) (*CREM* genotype: *P* = 0.005; study site: *P* = 0.001). No individual metabolites reached the *a priori* determined significance level of false discovery rate (FDR) < 0.05 in *post hoc* testing; however, seven metabolites reached FDR < 0.1, with six of these decreased in the homozygous reference (G) genotype in both Bangladeshi and American children, including the oxidized derivatives of linoleic acid, 9- and 13-hydroxyoctadecadienoic acid, a product generated enzymatically by macrophages (Fig. 2B) (27). Reduction in detected hydroxyoctadecadienoic acid concentration is consistent with reduced expression of immune-related genes in these subjects (Fig. 1). Individuals homozygous for the reference (G) allele had lower lactate and higher citrate levels (nominal *P*-values of 0.02 and 0.05, respectively) concordant with gene expression associated with increased mitochondrial respiration (Fig. 1B). A metabolite set enrichment analysis identified pathways across diverse metabolite classes that were altered between those homozygous for the reference (G) allele and heterozygotes (Fig. 2C). The most altered pathway by *P*-value was starch and sucrose metabolism; however, changes were identified in pathways associated with amino acids, lipids, nucleic acids, and carbohydrates. We concluded that in children, the reference allele for *CREM* locus variant rs2148483 was associated with resistance to amebic diarrhea and with lower weight-for-age z-scores (WAZ) at 1 year of age, increased metabolic gene transcription, and decreased metabolite levels.

### In mice, an inducible global deletion of *Crem* was associated with reduced body weight

**Fig 3:**
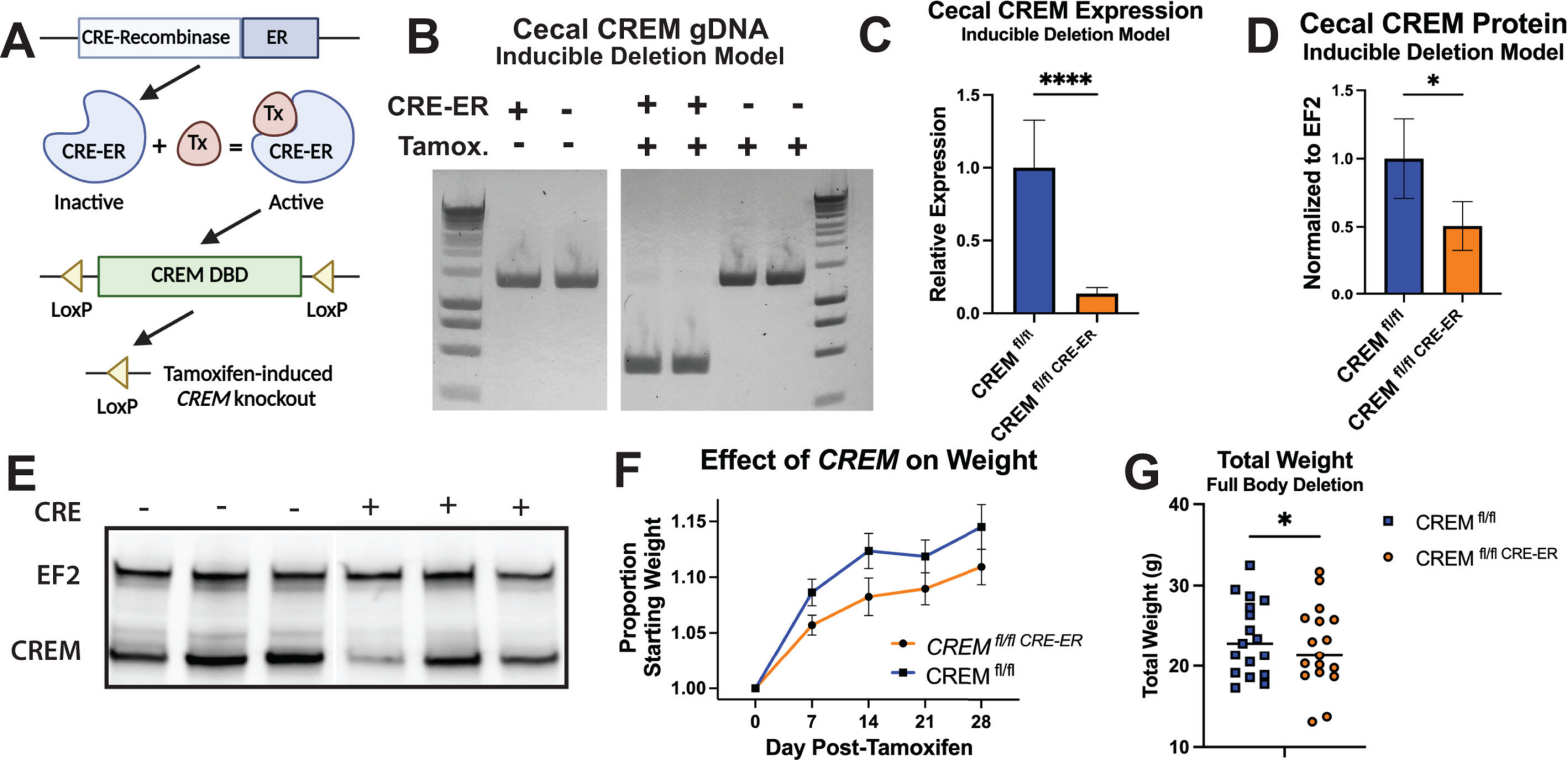
A full body model of *Crem* deletion reveals a role for *C*rem in body weight. (**A**) LoxP sites flank a portion of the *Crem* DNA binding domain necessary for function of all known *Crem* isoforms. (**B**) PCR amplification of a segment of CREM encompassing the LoxP sites shows robust excision after tamoxifen treatment. (**C**) RT-qPCR on cecal tissue for *Crem* normalized to *Gapdh* (*P* < 0.0001). (**D and E**) Western blotting and quantification of CREM protein (*P* = 0.028). (In panels B–E, samples were collected at least 4 weeks after tamoxifen administration.) (**F**) Body weight curve over 4 weeks following tamoxifen-induced *Crem* deletion. (**G**) Body weight data collected at least 4 weeks post-tamoxifen (*P* = 0.023). Bars in panels C, D, and F represent SEM.

Identification of causal genetic variants at the *CREM* locus has not been possible due to a region of linkage disequilibrium that includes not only *CREM* but the genes *CCNY* and *CUL2* (12). We discovered that reference alleles for the *CREM* locus rs1248483 were associated with alterations in *CREM*-regulated gene targets but not changes in *CCNY* or *CUL2* expression. Thus, we focused mechanistic studies in a mouse model on *CREM*. We generated a tamoxifen-inducible *Crem* deletion mouse with loxP sites flanking the *Crem* exon containing the basic motif of the bZIP DNA binding domain, which is necessary for the function of all *Crem* isoforms (Fig. 3A) (28). Efficient excision of the floxed DNA region within *Crem* was observed following 5 days of I.P. tamoxifen administration (Fig. 3B). Detection of *Crem* transcripts in cecal tissue of *Crem^fl^*^/*fl* CRE-ER^ mice was reduced 84% compared to *Crem^fl^*^/*fl*^ littermates (Fig. 3C). Reduction of CREM protein levels was significant (*P* = 0.02), but less marked, following induced deletion compared to the decrease in transcript abundance (Fig. 3D and E). Incomplete knockout at the protein level may reflect an extended protein half-life or antibody cross-reactivity with related transcription factor, CREB. While protein half-life data are not publicly available for *Crem*, the average half-life across tissues of the related transcription factors *Creb1* and *Atf1* was 3.5 and 10.4 days, respectively (29). Despite incomplete CREM knockout at the protein level, an effect on body weight was observed. Pairs of same-sex littermates, both male and female, were weighed at 7 day intervals for 28 days following completion of tamoxifen administration (median age at tamoxifen administration: 7.8 weeks ± 2.7 weeks SD). *CREM^fl^*^/*fl* CRE-ER^ mice weighed less compared to *Crem^fl^*^/*f*l^ littermates (Fig. 3F, *P* = 0.002; Fig. 3G, *P* = 0.0233). This change in body weight was independent of intestinal microbiota: we found no differences in gut microbial diversity as measured by sequencing between *Crem^fl^*^/*fl*^ and *Crem^fl^*^/*fl* CRE-ER^ (Fig. S3).

### Conditional deletion of *Crem* in mice affected metabolic and immune pathway transcription after *E. histolytica* challenge

**Fig 4:**
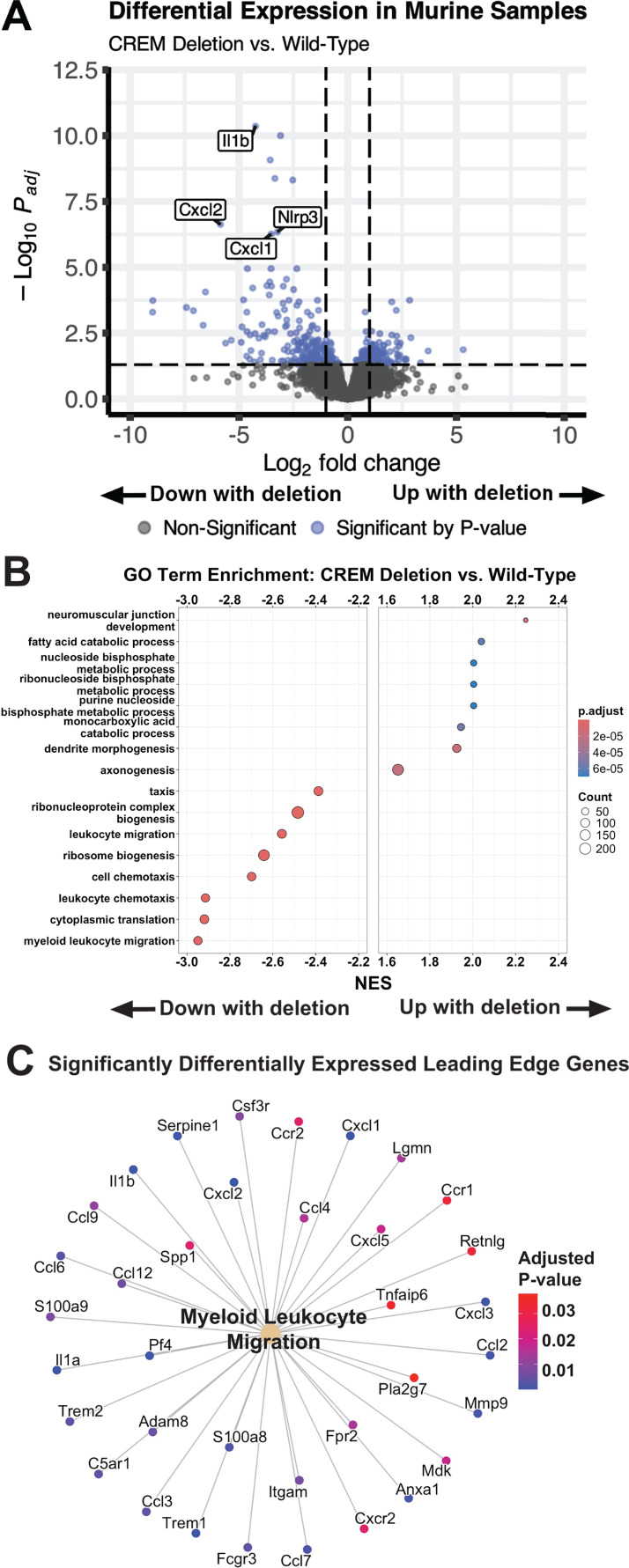
Full-body *Crem* knockout alters immune and metabolic gene expression in cecal tissue. (**A**) Volcano plot of differentially expressed genes by genotype in tissue harvested 72 h after *E. histolytica* challenge. (**B**) Gene set enrichment analysis of biological process GO terms. (**C**) Significantly differentially expressed genes contributing to the leading edge of the most downregulated pathway by normalized enrichment score (NES). Gene nodes colored by adjusted *P*-value from DESeq2 results; all genes displayed had negative log2 fold change. *N* = 4 mice per group.

To assess the role of *Crem* on intestinal gene expression, RNAseq was conducted on cecal tissue from *E. histolytica*-challenged *Crem^fl^*^/*fl*^ and *Crem^fl^*^/*fl* CRE-ER^ mice. The murine model of *E. histolytica* challenge replicates several key features of human pathology, including mucosal thickening and ulceration of the mucosa (30). In this challenge model, 385 genes were differentially expressed between *Crem^fl^*^/*fl* CRE-ER^ and *Crem^fl^*^/*fl*^ mice using a cutoff of adjusted *P*-value < 0.05 (Fig. 4A; File S1). One hundred sixty-one of these genes were upregulated while 224 were downregulated. Transcription of genes neighboring *Crem*, *Ccny*, and *Cul2* was not affected by *Crem* deletion (File S1). Similar to the findings from non-*E*. *histolytica*-challenged human duodenal samples homozygous for the rs2148483 reference allele, *Crem^fl^*^/*fl* CRE-ER^ mice showed alteration in immune genes, including downregulation of innate immune cell activation and migration (Fig. 4). A role for *Crem* in regulating the NLRP3 inflammasome during amebiasis was suggested by downregulation of *Il1b* and *Nlrp3* transcripts during acute challenge (File S1). Several genes involved in myeloid chemotaxis were within the 10 top downregulated DEGs determined by *P*-value, including *Cxcl1* and *Cxcl2*. GSEA for biological process GO terms identified enrichment of several processes associated with downregulated immune cell migration gene expression in *Crem^fl^*^/*fl* CRE-ER^ mice (Fig. 4B and C; File S1). A 32-plex cytokine panel did not detect any significant differences in *Crem^fl^*^/*fl*^ and *Crem^fl^*^/*fl* CRE-ER^ mouse plasma 72 h after *E. histolytica* challenge, suggesting *Crem*-dependent changes in immunity are largely confined to the site of infection (File S1).

Increased gene expression in *Crem*-deficient mice was associated with processes related to metabolite catabolism (monocarboxylic acid catabolic process: *P* = 5.58 × 10^−5^; fatty acid catabolic process: *P* = 6.11 × 10^−5^; Fig. 4B). Based on the observation of altered metabolic process transcription, we assessed systemic respiration and activity using Oxymax/CLAMS metabolic cages. *Crem* deletion mice showed a trend toward increased VO_2_ consumption and VCO_2_ production (Fig. S4A). However, activity and respiratory exchange rate were unchanged (Fig. S4A), as were food intake, intestinal permeability, and whole body water mass (Fig. S4B to D). Glucose and insulin sensitivity, as well as key adipokine levels including leptin, were similarly unchanged (Fig. S5).

### *Crem* deletion in mice in intestinal epithelial cells led to reduced weight

**Fig 5:**
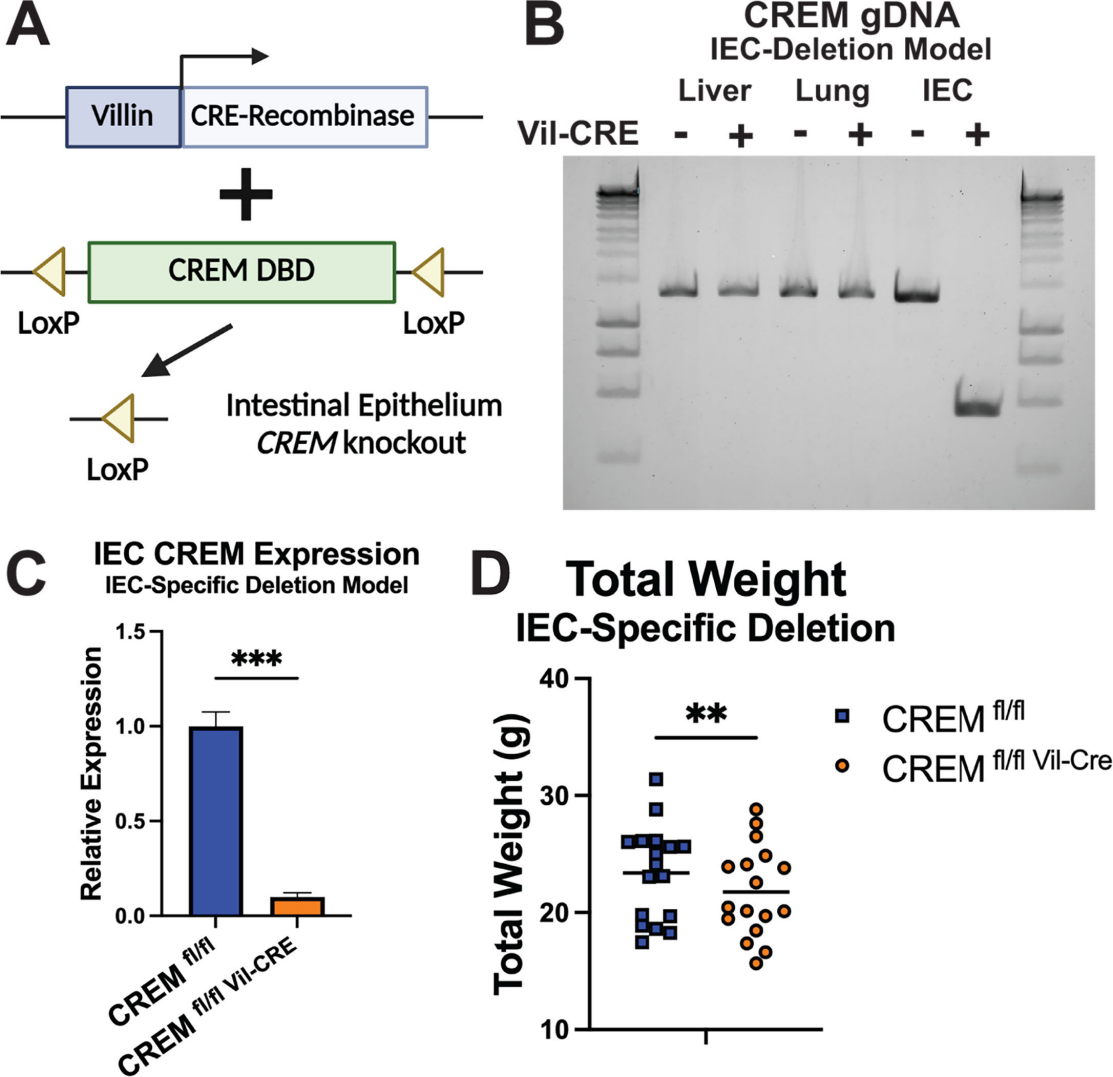
An IEC-specific model of *Crem* deletion is sufficient to alter body weight. (**A**) LoxP sites flank a portion of the *Crem* DNA binding domain necessary for the function of all known *Crem* isoforms. CRE recombinase is expressed under control of the villin 1 promoter. (**B**) PCR amplification of a segment of *Crem* encompassing the LoxP sites shows robust excision. (**C**) RT-qPCR on cecal epithelial cells for *Crem* normalized to *Gapdh* (*P* = 0.0003). Bars represent SEM. (**D**) Body weight data collected from male and female mice (*P* = 0.001).

We sought to understand in which tissue(s) *Crem* is acting to drive altered body weight and immunity. Previous work showed higher levels of apoptotic intestinal epithelial cells (IECs) in *Crem*^–/–^ mice during amebic infection (12). Thus, *Crem* action in IECs may be partially or completely responsible for *Crem*-mediated body weight and immune phenotypes. Mice homozygous for loxP sites around the *Crem* DNA binding domain were crossed as shown in Fig. 3 to mice harboring CRE recombinase under control of the mouse villin 1 promoter (Fig. 5A). Excision of the floxed DNA region within *Crem* was seen in IECs but not in other tested tissues (Fig. 5B). Detection of *Crem* transcripts in cecal IECs of *Crem^fl^*^/*fl* Vil-CRE^ mice was reduced 90% compared to *Crem^fl^*^/*fl*^ littermates (Fig. 5C). Body weight data collected from pairs of same-sex littermates showed a decrease in *Crem^fl^*^/*fl* Vil-CRE^ mice compared to *Crem^fl^*^/*fl*^ mice (Fig. 5D). Meta-analysis of human WAZ measurements at 1 year of age across three cohorts revealed an association of *CREM* alleles with reduced body weight while adjusting for infection history (Table 2). Additionally, both full body-inducible *Crem* deletion and IEC-specific *Crem* deletion models resulted in reduced body weight in the absence of infection (Fig. 3F and G; Fig. 5C). Together, these data show that immune challenge is not necessary to drive the *CREM*-dependent body weight phenotype.

### *Crem* in intestinal epithelial cells affects metabolic but not immune pathway transcription

**Fig 6:**
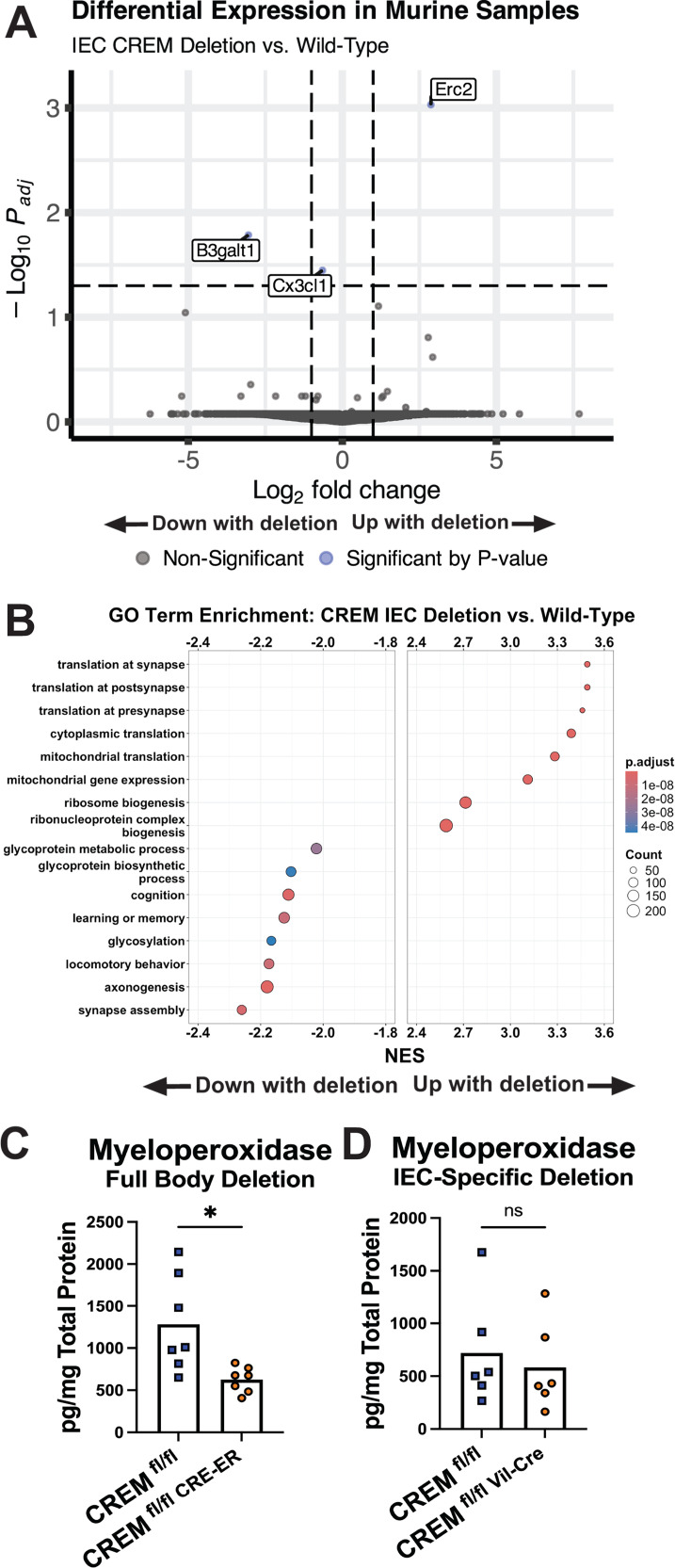
IEC-specific deletion of *Crem* altered metabolic gene expression in the cecum. (**A**) Volcano plot of differentially expressed genes by genotype on tissue harvested 72 h after *E. histolytica* challenge. (**B**) Gene set enrichment analysis using biological process GO terms. *N* = 5 mice per group. (**C and D**) Myeloperoxidase protein by enzyme-linked immunosorbent assay (ELISA) from cecal lysate collected 72 h after *E. histolytica* challenge from full body (*P* = 0.016) and IEC-specific *Crem* deletion (*P* = 0.438). *N* = 6–7 mice per group.

To understand the contribution of *Crem* to transcription, RNAseq was performed on cecal tissue from *E. histolytica*-challenged *Crem^fl^*^/*fl*^ and *Crem^fl^*^/*fl* Vil-CRE^ mice. Only three genes were differentially expressed between *Crem^fl^*^/*fl* Vil-CRE^ and *Crem^fl^*^/*fl*^ mice (Fig. 6A; File S1); *Erc2* was upregulated while *B3galt1* and *Cx3cl1* were downregulated. Notably, each of these genes is known to be expressed in neurons. A GSEA for biological process GO terms identified enrichment of pathways associated with neuronal-related processes, such as translation at synapses and axonogenesis (Fig. 6B; File S1). Additionally, IEC-specific *Crem*-deficient mice were positively enriched for pathways related to metabolic processes, specifically for mitochondrial functions (Fig. 6B). However, GSEA did not show underrepresentation of immune cell migration processes for *Crem^fl^*^/*fl* Vil-CRE^ mice in contrast to the full body-inducible deletion model (Fig. 6B). Global *Crem* deletion mice had lower cecal myeloperoxidase (MPO) protein compared to *Crem*-sufficient controls (Fig. 6C), whereas IEC-specific *Crem* deletion mice showed no difference (Fig. 6D).

## DISCUSSION

The most important finding of this work is that *CREM* locus reference alleles at rs2148483 and rs58000832, previously demonstrated to be associated with resistance to *E. histolytica* diarrhea*,* were associated with reduced WAZ in infants living in a low-income community in Bangladesh. The *CREM* locus reference allele at rs2148483 was associated in the human small intestine with increased expression of *CREM*-regulated genes involved in mitochondrial function and downregulated expression of genes associated with broad immune function. Plasma metabolomics additionally demonstrated that the *CREM* locus reference allele was associated with increased mitochondrial respiration.

The discovery that our identified *CREM* locus reference allele was associated with alterations in *CREM*-regulated gene expression but not changes in *CCNY* or *CUL2* mRNA enabled us to ascribe the *CREM* locus reference allele phenotypes affecting diarrheal disease and undernutrition to *CREM* itself. This conclusion was supported by conditional deletion of *Crem* in mouse models, which not only demonstrated weight loss with whole body conditional deletion of CREM but additionally identified the intestinal epithelium as the site of *Crem* action affecting body weight but not immune function. A picture therefore emerges of *CREM*-regulated gene expression in the intestinal epithelium regulating susceptibility to undernutrition.

### Undernutrition and immunodeficiency

Undernutrition and infection susceptibility have long been known to be correlated (9, 31). However, the underlying mechanism leading to comorbidity of these conditions remains incompletely understood. Our findings indicate host genetic variation within a single genetic locus can contribute simultaneously to both *E. histolytica* diarrhea susceptibility and WAZ. The reference alleles (rs2148483 and rs58000832) were associated with decreased risk for *E. histolytica* diarrhea and increased risk of undernutrition.

It has previously been shown that the rs2148483 and rs58000832 reference alleles were associated with resistance to symptomatic *E. histolytica* infection in Bangladeshi children (12). In concordance with this, full-body *CREM* deletion mice were shown to have increased susceptibility to amebiasis infection (12). In the present study, RNAseq analysis of full body-inducible *Crem* deletion mice revealed downregulation of expression of innate immune-related processes. Altered activity in individuals harboring *CREM* locus SNPs or in *Crem* deletion mice plausibly may lead to increased amebiasis susceptibility through an insufficient immune response causing delayed clearance of infection.

IEC-specific *Crem* deletion mice did not show differential expression of immune-related genes relative to *Crem*-sufficient mice, suggesting different tissue sites of *Crem* activity for undernutrition and immunity. When considered in context with the reduced weight phenotype seen in IEC-specific *Crem* deletion, these data also indicate that decreased body weight is not the driver of *Crem*-related immunodeficiency.

One limitation of our study is that the conditional deletion of *CREM* in mice, while key in demonstrating a role for *CREM* in nutrition and immunity, did not precisely recapitulate the phenotypes associated with *CREM* locus variants in humans. Undernutrition and *E. histolytica*-positive diarrhea susceptibility were independently associated in opposite directions with the same alleles in humans, but in the same direction with deletion in mice. While the generation of a mouse model by allele-specific editing would be ideal, it has not been possible to identify the causative genetic variant(s) due to a region of strong linkage disequilibrium at the *CREM* locus (12). Without knowledge of causal variants, rs2148483 and rs58000832 alleles should be considered only as tag SNPs for our associated phenotypes, as it is probable our tested SNPs are not the drivers of altered undernutrition and *E. histolytica* diarrhea susceptibility but are in high linkage disequilibrium with the true variant(s). Further complicating our understanding, our two phenotypes may be mediated by different variants in the same block of linkage disequilibrium. Future investigations will be required to fine-map variants within the *CREM* locus associated with undernutrition and *E. histolytica*-positive diarrhea susceptibility for putative causal effects.

An additional caveat of our animal model is that mouse developmental stage did not closely mimic that of Bangladeshi cohort children measured for WAZ at 1 year of age. Conditional deletion of murine *Crem* occurred at a median age of <8 weeks old, placing our mouse developmental stage akin to late human adolescence. American children of adolescent age (median: 11.4 years) studied in stratified transcriptomic and metabolomic analyses displayed highly concordant results to tested Bangladeshi samples, suggesting *CREM* affects undernutrition and diarrheal disease susceptibility beyond infancy. However, further experiments using weaning age mice are necessary to determine if the role of *CREM* changes throughout development.

### *CREM* in other immune-mediated conditions

Variants in the *CREM* locus have previously been associated through GWAS with inflammatory bowel disease (IBD), including Crohn’s disease and ulcerative colitis (32–36). IBD results from inappropriate immune responses against commensal bacteria leading to inflammation of the gastrointestinal tract (37). Thus far, mechanistic studies of *CREM* in IBD have been limited to T-cell-specific overexpression of the *CREMa* isoform in mice subjected to DSS-induced colitis (38). *CREMa* overexpressors displayed enhanced disease activity, suggesting that while protective in amebiasis, the role of *CREM* in immune activation and chemotaxis may be pathogenic in the context of IBD. Further research is merited to delineate the specific contribution of *CREM* in individual immune cell populations in infectious and non-infectious colitis.

Beyond colitis, variants within the high LD region at the *CREM* locus have been associated in genome-wide analyses with eosinophil count and eosinophilic esophagitis risk (39, 40). *CREM* locus variants have not been associated to date by GWAS with autoimmune disease. However, a targeted analysis of variants identified a significant association between systemic lupus erythematosus (SLE) and non-coding *CREM* SNPs rs2295415 and rs1057108 (41). Robust literature supports a role for *CREM* in SLE and other systemic and organ-specific autoimmune conditions, including psoriasis, rheumatoid arthritis, and oligoarticular juvenile idiopathic arthritis (21, 42–45).

Elevated *CREM* expression in patients with autoimmune conditions contributes to dysregulation of cytokine production in T lymphocytes, skewing activity toward type 17-mediated immunity by upregulation of IL-17A and IL-21 and downregulation of IL-2 (38, 46, 47). Notably, *CREM* exerts effects on immune modulation not only by direct binding to promoter sites but via secondary co-recruitment of epigenetic modification enzymes that alter chromatin accessibility, including DNA methyltransferase 3 alpha, histone deacetylase 1, and the histone acetyltransferase p300 (43, 48–51). While *CREM* locus variants rs2148483 and rs58000832 have not been identified as risk factors for systemic autoimmune disease, in our work, the rs2148483 reference allele (associated with decreased risk of *E. histolytica*-positive diarrhea) was associated with downregulation of key lymphocyte functions. This suggests that decreased *CREM* activity in lymphocytes may be protective against both infectious and autoimmune conditions.

### *CREM* in intestinal epithelial cell metabolism

RNAseq analysis of human small intestine and mouse cecum demonstrated an association of *CREM* with expression of metabolic process genes, particularly those related to catabolism and mitochondrial processes. *CREM* modulates expression of catalytic subunit 2 of pyruvate dehydrogenase phosphatase (PDP2), a key enzyme controlling advancement of pyruvate into oxidative phosphorylation (OxPhos). Without *CREM*, reduced PDP2 leaves pyruvate dehydrogenase in its phosphorylated, inactive form, thereby restricting progression to mitochondrial respiration (23). Th17-polarized T cells from *Crem*^–/–^ mice have previously been shown to have reduced glycolytic capacity compared to *Crem*^+/+^ cells, consistent with a shift to OxPhos for ATP generation (23). Diversion of pyruvate to OxPhos reduces the accumulation of upstream glycolytic metabolites and flux into branching biosynthetic pathways that emanate from glycolysis, such as the pentose phosphate pathway. No significant differences were detected in metabolic caging experiments of *Crem^fl^*^/*fl* CRE-ER^ mice. This may be due to the inability of our metabolic caging system to detect subtle changes in VO_2_ consumption and VCO_2_ production over the variation within and between experiments. Alternatively, *Crem* expression may only be important for altering catabolism and mitochondrial processes in a subset of cell types, such as IECs, making body-wide changes unlikely to be detected by this system.

Our study revealed that the deletion of *Crem* in IECs was sufficient to reduce overall body weight and alter gene expression of mitochondrial-related pathways, indicating that metabolic changes in IECs plausibly drive the observed undernutrition phenotype. If a *Crem*-dependent metabolic shift toward OxPhos in IECs reduces flux through important biosynthetic pathways branching from glycolysis, proliferation or maturation of IECs may be impaired, compromising function as previously reported in intestinal stem cells (52, 53). Future research will be required to further mechanistically understand how metabolic changes in IECs alter cell function and contribute to body weight.

Undernutrition and diarrheal disease represent two of the most important global health issues affecting young children. These conditions have long been understood to coincide, but the environmental and genetic factors affecting susceptibility are complex and remain incompletely understood. Our results establish common variants in the loci for the transcription factor, *CREM*, as having a modulatory role simultaneously in both undernutrition and immune protection against diarrheal disease.

## MATERIALS AND METHODS

### Genetic association of WAZ

Study design and genotyping of Bangladeshi prospective birth cohorts (Table 1) were described previously (12, 54). SNPs were mapped to Human Genome Build 37. We performed a linear regression using an additive model to assess WAZ at 1 year of age for the two genome-wide significant SNPs in the previously published study (rs58000832 and rs2148483). The reference and alternate alleles at rs58000832 are annotated as C > CA in this manuscript where CA corresponds to the insertion annotated as dupA in NCBI Single Nucleotide Polymorphism Database Build 156. Associations for each cohort were estimated separately in PLINK 1.9 (55). The first two study-specific principal components from previous GWAS were included as covariates to account for population structure and genetic ancestry (12, 54). DBC and PROVIDE were also adjusted for symptomatic *E. histolytica* infection within the first year of life to control for a potential effect on WAZ due to the added frequency of *E. histolytica* diarrhea associated with *CREM* locus polymorphisms. CBC was not adjusted for cases of symptomatic *E. histolytica* infection because the number of *E. histolytica* positive children was very low (*N* = 4), and there was no association found between WAZ at 1 year and *E. histolytica* history in CBC. DBC was additionally adjusted for genotyping array. Sex was not included in any model as it was not found to be associated with WAZ at 1 year in any of the three cohorts. Association results for each cohort were subsequently combined into a fixed-effect meta-analysis also in PLINK 1.9.

### Human small intestinal RNA sequencing

Small intestinal biopsies were originally collected as part of the Bangladeshi Environmental Enteric Dysfunction study (ClinicalTrials.gov number, NCT02812615) (25). Differential expression was determined by comparing individuals with the rs2148483 homozygous reference allele to heterozygous individuals using DESeq2 in R v.4.3.1 (56). Study site, sex, and rs2148483 genotype were included in the DESeq2 analysis design. Age was excluded as a covariate due to collinearity with study site. Seventy-two of 81 American site individuals were school-aged (5–18 years of age), while Bangladeshi site individuals were either preschool-aged (0–5 years of age: *N* = 37) or adults (>18 years of age: *N* = 81). Genes were considered significantly different if the adjusted *P*-value was <0.05. Significantly DEGs were compared to genes in the ChIP-Atlas database identified by chromatin immunoprecipitation sequencing to be bound by *CREM* within 5 kb of transcriptional start site (File S1) (26). A volcano plot was generated from full DESeq2 results using the EnhancedVolcano package in R (57). Gene set enrichment analysis was performed using the gseGO function of the clusterProfiler package with a minimum gene set size of 10 and Benjamini-Hochberg correction for multiple testing (58). The full gene list of DESeq2 results was input to gseGO ranked by Wald statistic. For differential isoform expression analysis, the Salmon package was used for quasi-alignment mapping of RNAseq reads from raw fastq files (59). Calculations of isoform fraction were performed using the IsoformSwitchAnalyzeR package (60). Isoform switch results were filtered for *CREM* isoforms accounting for at least a 1% fraction of all *CREM* isoforms averaged across samples. Significant differences were assessed between homozygous reference allele to heterozygous groups by multiple Mann-Whitney *U*-tests followed by Benjamini-Hochberg correction.

### Human small intestinal genotyping

DNA from human small intestine was diluted using nuclease-free water to a concentration of 1–10 ng per microliter. DNA was then combined with TaqMan Genotyping Master Mix (Thermo Fisher 4371355) and TaqMan Genotyping Assay Mix (Thermo Fisher Assay ID C___9584184_10) according to the manufacturer’s specifications. Samples were run on a CFX Opus 96 Real-Time Detection System (Bio-Rad). CFX Maestro Software (Bio-Rad) was used for automatic allelic discrimination of results. Alternate allele frequency for rs2148483 (A) was 0.41 and 0.33 for American and Bangladeshi groups, respectively, within our data set. Bangladeshi individuals were all ethnically Bengali. American individuals were self-reported to be 84% White or Caucasian, 5% African American, and 11% Other. SNP associations with WAZ were not conducted for intestinal genotyping samples because WAZ is not an applicable metric for individuals over 120 months of age. For children older than 120 months, body mass-for-age Z-score was calculated in place of WAZ according to WHO standard for Table S1. Z-score anthropometry was not calculated for adults.

### Untargeted metabolomics

Human plasma samples were shipped on dry ice to Metabolon, Inc. (Durham, NC), for untargeted metabolomics analysis. All sample preparations and metabolite identifications were performed according to standard protocols of Metabolon, Inc. (briefly summarized here). Samples were prepared using the automated MicroLab STAR system from Hamilton Company. Several recovery standards were added prior to the first step in the extraction process for QC purposes. To remove protein, dissociate small molecules bound to protein or trapped in the precipitated protein matrix, and to recover chemically diverse metabolites, proteins were precipitated with methanol under vigorous shaking for 2 min (Glen Mills GenoGrinder 2000) followed by centrifugation. The resulting samples were divided into aliquots and placed briefly on a TurboVap (Zymark) to remove the organic solvent.

Metabolites were identified by Metabolon, Inc. (Durham, NC) using Waters ACQUITY ultra-performance liquid chromatography (UPLC) and a Thermo Scientific Q-Exactive high resolution/accurate mass spectrometer interfaced with a heated electrospray ionization (HESI-II) source and Orbitrap mass analyzer operated at 35,000 mass resolution. Sample extracts were separated into aliquots, dried, and suspended in appropriate standard-containing solvents for analysis by four methods. These four methods facilitate the measurement of metabolites with different biochemical properties and include two acidic positive ion condition methods, one optimized for hydrophilic compounds and one optimized for hydrophobic compounds, and a third basic negative ion optimized method. Finally, a UPLC/mass spectrometry (MS)/MS method with negative ionization following elution from a hydrophilic interaction chromatography column was used. Compounds were identified by comparison to library entries of purified standards or recurrent unknown entities. Metabolon maintains a library based on authenticated standards that contains the retention time/index (RI), mass to charge ratio (*m/z*), and chromatographic data (including MS/MS spectral data) on all molecules present in the library.

All features above the limit of detection in at least 80% of samples were included in the analysis. These features were imputed using Random Forest imputation, log2 transformed, centered, scaled, and normalized to sample volume. The pre-processed data were analyzed by PERMANOVA using the vegan R package to explain metabolite profiles by rs2148483 genotype and study site. PERMANOVA was followed by multiple *t*-tests, and *P*-values were adjusted using the Benjamini-Hochberg correction. Pre-processed data were uploaded to Metaboanalyst 6.0 Quantitative Enrichment Analysis (61). Only features with KEGG or HMDB IDs were recognized within Metaboanalyst software and tested against KEGG pathways for enrichment.

### *Crem* deletion mice

*Crem* floxed mice were engineered by InGenious Targeting Laboratory. loxP sites were inserted around an exon of the protein’s bZIP DNA binding domain, which is present in all known isoforms. Further information on loxP insertion sites is available in Text S1. Targeted iTL BF1 (C57BL/6 FLP) embryonic stem cells were microinjected into Balb/c blastocysts. Chimera pups with a high percentage of black coat color were selected for construct integration and mated back to wild-type (WT) C57BL/6J. To generate full body-inducible deletion mice, *Crem^fl^*^/*fl*^ mice were crossed with B6.Cg-Tg(CAG-cre/Esr1*)5Amc/J (The Jackson Laboratory) to generate *Crem^fl^*^/*fl* CRE-ER^ mice. *Crem^fl^*^/*fl*^ and *Crem^fl^*^/*fl* CRE-ER^ were administered 100 µL of 10 mg/mL tamoxifen (Sigma‐Aldrich) dissolved in sterile corn oil (Sigma‐Aldrich) by I.P. daily for 5 consecutive days to induce recombination. To generate intestinal epithelium-specific deletion mice, *Crem^fl^*^/*fl*^ mice were crossed with B6.Cg-Tg(Vil1-cre)1000Gum/J (The Jackson Laboratory) to generate *Crem^fl^*^/fl Vil-CRE^ mice.

### Confirmation of Crem deletion

DNA was extracted from cecal tissue using a DNeasy Blood and Tissue Kit (Qiagen). Deletion of the loxP-flanked DNA sequence was confirmed by PCR using the following primers at an annealing temperature of 50°C: Forward: GGTGGAATTTGCTACAGTGGAAGT; Reverse: AGGGGTAAAATGTACTTACCAGG. RNA was extracted from cecal tissue using an RNeasy Mini Kit (Qiagen) followed by genomic DNA removal with a Turbo DNA-free Kit (Invitrogen). RNA was reverse transcribed to cDNA using a High-Capacity cDNA Reverse Transcription kit (Applied Biosystems). Templates were amplified with PrimePCR Probe Assays (Bio-Rad) for *Crem* and *Gapdh* (housekeeping gene) using the CFX Opus 96 Real-Time Detection System (Bio-Rad). Relative quantification was calculated using the 2^-ddCt^ method. Protein for immunoblot confirmation was extracted by adding 300 µL of lysis buffer 1 (1× HALT protease inhibitor cocktail from Thermo Fisher in 5 mM HEPES) followed by bead beating for 1 min. After bead beating, 300 µL of lysis buffer 2 (1× HALT protease inhibitor cocktail in 5 mM HEPES with 2% Triton X-100) was added with the solution and incubated on ice for 30 min. Solutions were centrifuged for 10 min at 10,000 × *g*, and supernatants were collected. Samples were prepared with 2× Laemmli sample buffer (Bio-Rad) and boiled for 5 min at 95°C. Protein was run on a 4-20% Mini-Protean TGX polyacrylamide gel (Bio-Rad), transferred to an Immun-Blot PVDF Membrane (Bio-Rad), and blocked using Tris-buffered saline with Tween-20 plus 5% Blotting Grade Blocker (Bio-Rad). Anti-CREM A-2 and anti-EF-2 C-9 conjugated to Alexa 488 were used at a 1:250 and 1:1,500 dilution, respectively, followed by m-IgG Fc BP-HRP at 1:100. All antibodies were sourced from Santa Cruz Biotechnology.

### Microbiome analysis

Stool pellets were collected prior to tamoxifen treatment and again from the same mice four weeks after tamoxifen treatment. *Crem^fl/fl^* and *Crem^fl/fl^* ^CRE-ER^ mice were cohoused prior to and throughout sample collection in a manner consistent with experiments conducted in Fig. 3 and 4. Stool samples were immediately transferred to TransnetYX microbiome buffer solution. Samples were shipped to TransnetYX for microbiome analysis using shallow shotgun whole genome sequencing with a read depth of 2 million paired-end reads. Results from TransnetYX were analyzed using the OneCodex platform. Shannon indices and Bray-Curtis dissimilarity values for alpha and beta diversity analyses were downloaded from OneCodex along with relative taxa abundance values. Species-level data identified 301 species. Relative species abundance values were log transformed and then analyzed by PERMANOVA using the vegan R package to determine if microbiome was associated with *Crem* genotype, tamoxifen administration, or an interaction between genotype and tamoxifen.

### Mouse cecal RNAseq

Mice were challenged with 2 × 10^6^ *E. histolytica* trophozoites by laparotomy followed by direct intra-cecal injection as previously described (62). Seventy-two hours post-surgery, mice were euthanized for organ harvest. Tissue was collected from the apex of the cecum and immediately placed in RNAlater (Invitrogen). RNA was extracted, and genomic DNA was removed as described in “Confirmation of Crem deletion*”* above. Total RNA was shipped to Novogene for paired-end RNAseq with polyA selection. Raw reads were checked for quality using FASTQC v.0.11.5 and trimmed to remove adapters using bbmap v.39.01. Salmon v.1.10.1 was used for “quasi-mapping” and quantification (59). Differential expression and gene set enrichment analyses were conducted as described above in “Human small intestinal RNAseq.”

### Mouse cecal ELISA and plasma cytokine measurements

Mice were challenged with *E. histolytica* trophozoites as described above. Protein was extracted from dissected cecal tissue by addition of lysis buffer 1 (1× HALT protease inhibitor, 5 mM HEPES) followed by bead beating for 2 min. Lysis buffer 2 (1× HALT protease inhibitor, 5 mM HEPES, 2% Triton X-100) was then added, and samples were incubated on ice for 30 min. Lysates were centrifuged for 10 min at 10,000 × *g*, and the supernatant was collected. MPO protein concentration was measured using an R&D enzyme-linked immunosorbent assay (ELISA) kit (Minneapolis, MN). MPO concentration was normalized to total tissue protein.

Mouse plasma was collected 72 h after intra-cecal challenge with *E. histolytica* (*Crem*^*fl*/*fl*^ *N* = 4; *Crem*^*fl*/*fl* CRE-ER^ *N* = 3). The Mouse Cytokine/Chemokine 32-Plex Discovery Assay Array from Luminex was run according to the manufacturer’s protocol at the UVA Flow Cytometry Core Facility. Twenty-nine cytokines were quantifiable in all samples and subsequently tested for significance using a Welch’s *t*-test.

### Metabolic tolerance tests

Metabolic tolerance tests were conducted as previously described (63). Animals were fasted for 6 h prior to metabolic tolerance testing, including moving animals to cages with clean bedding to limit coprophagy. For insulin tolerance tests, animals were administered 0.75 IU Novolog insulin (Novo-Nordisk) per kg body mass by I.P. injection. Blood glucose was measured using an Accu-Check Guide Me glucometer by collecting blood from a tail snip immediately prior to insulin injection (0 min) and again 15, 30, 60, and 90 min post-injection. For glucose tolerance tests, animals were administered 1.5 g glucose per kg body mass by I.P. injection in the form of 10% glucose dissolved in sterile saline. Blood glucose was measured immediately prior to glucose injection and at 15, 30, 60, 90, and 120 min post-injection.

### Metabolic cage measurements

Mice were individually housed in Oxymax/CLAMS cages (Columbus Instruments) for 4 days at a time. Mice had free access to food and water at all times. The same mice were measured using this system prior to tamoxifen administration and again 2 weeks post-tamoxifen. Data were analyzed using the CLAMS data eXamination Tool (CLAX) software (Columbus Instruments). The first 48 h of data collected were considered an acclimation period and excluded from the results. Values were partitioned into those taken during the “light” and “dark” portions of the vivarium lighting cycle. For each mouse, post-tamoxifen measurements were subtracted from pre-tamoxifen measurements to yield change over time per animal. Water mass was measured using an EchoMRI at a single time point 1 or 3 weeks post-tamoxifen treatment.

### Intestinal permeability assay

Mice were fasted for 4 h prior to FITC-Dextran administration, including moving animals to cages with clean bedding to limit coprophagy. Mice were gavaged with 40 mg per 100 g body weight of 4 kDa fluorescein isothiocyanate-dextran solution (Sigma Aldrich). Mice were returned to home cages for an additional 4 h of fasting. Blood was collected by facial vein puncture and allowed to clot at room temperature. Serum was collected by centrifugation, and fluorescence was measured using a Synergy H4 Hybrid plate reader (BioTek) set to 485 nm excitation and 530 nm emission.

## Data Availability

Raw mouse RNAseq data files are available on NCBI’s Gene Expression Omnibus (GEO) accession number GSE276451. RNAseq counts files and R code for RNAseq and metabolomics are available at https://github.com/petrilab-uva/CREM.git. Full differential expression analysis and gene set enrichment analysis results are available in File S1.

## SUPPLEMENTAL MATERIAL

File S1 mbio.01390-25-s0001.xlsx Results for differential expression analysis, GSEA, and cytokine data.

Text S1 mbio.01390-25-s0002.docx The CREM gene.

Fig. S1 mbio.01390-25-s0003.eps WAZ across cohorts.

Fig. S2 mbio.01390-25-s0004.eps CREM isoform specific expression

Fig. S3 mbio.01390-25-s0005.eps Microbiome of CREM mice.

Fig. S4 mbio.01390-25-s0006.eps Metabolic characterization.

Fig. S5 mbio.01390-25-s0007.eps Metabolic tolerance tests.

Legends mbio.01390-25-s0008.docx Supplemental figure legends.

Table S1 mbio.01390-25-s0009.docx Characteristics of individuals providing small intestinal biopsies for transcriptomics.

ASM does not own the copyrights to Supplemental Material that may be linked to, or accessed through, an article. The authors have granted ASM a non-exclusive, world-wide license to publish the Supplemental Material files. Please contact the corresponding author directly for reuse.
