# Supplementary material for: The cAMP responsive element modulator (CREM) transcription factor influences susceptibility to undernutrition and infection
Source: mBio. 2025 Jun 27;16(8):e01390-25. doi: 10.1128/mbio.01390-25 (PMC12345263; doi:10.1128/mbio.01390-25)
Supplement: Text S1 — The CREM gene. [file mbio.01390-25-s0002.docx]

Mus musculus cAMP Responsive Element Modulator sequence.

Exons are colored in red and bolded.

Primer sites used for amplification of DNA products in Figure 3B are bolded without added color.

Additional notes on inserted sequences from cloning in bold surrounded by [brackets] without added color.

GCGCACGCGCGGAAGTTGTAGCTTCCGTTTTCCTGTCCCACCCCTTAGCTCTTCCTAGTC

ATCTGCCTCAACAAGGCGTCCTCCTCCAATACGCCCCGTC**CCCAAGGCTCCGCCCCGACC**

**CCCGTTCTATTTAATTGTTGGATTGTCACCTCTGCAATCCCGCTGACTGCCAGCAGGGGG**

**CAGAGTTCGAGCCTGCAACTTTTTTTCTCCTCCGACCCAGTCCCCTTAGAGAGGCGATCG**

**GCGTGGGGGAGGGGAGGGTAGGGCTCGAGGCCGCAGTTCGGTCCGCTGCTTGCGCGGCTT**

**TCTGTTCGCGTCGGCAGAGAGGCCGCGACAACCGCATCAGAGCTGACGTGGGCGCGACGT**

**GCGGCCGCATCCGCCGGGTTGCCTGGGCGGCGGCCGTCGGGCTCTGCGTCCCCACCTCCT**

**CCCGTCCGTAATCAGTGACGAGGTCCGCTACGTAAACCCTTCGCGGCGG**GTAAGTGGCGC

GCCTGCCTCGCATCACAGCCCGCGGCGGGCGGCGGCTGCTCGGGACGCGCCTTTCCGCCC

GGCCTGCCCTCACCGCCGCCCGCCCGCCCGCCCGCTCGCGGCTCGCCACCGAGAGCTCCC

CGGGCACGCAGCGGGCCGCCTGCGCTCGCCCTCTTCCGTGCTTCGTCTTGGTACCGACGA

GAAAGCCTACCGTCAGAGAGCCCTCCGGGCGGCCGCTCGGGCTTCTCAGTGTGACAGGAA

AAGGGCGTCGGTCCTGCTCGCCACAGTCCTGGGCCCGCAGTAGCGCTTTCGTCCCAGCGT

TGCGT**TCGCAGTGTAGATAGAAAATAACGTGCCCCATTTCTTCCAGAAGGGTGGCGCAGG**

**GACTGCACCATCGAGTCATTTTTGTCCCCACACAATATAGAGCTGGAG**GTGCGGTGGCGA

GCGGCCGGGCGAGTCGCAGTTGGGGGGTCCGAGGGTCGACTACTGGATTTCCAGACCCGG

AGTAGGGGAGACGTGTCCAAAACAGTCTTGTCGCCTGTTACAGGCGTAACTAACCGCCAG

CGTTAAGTGGCAGGCGCCTACGGTCCCAGGACCTTACCGGGAAAGCTGCTGTCTGCTAGT

CAGCACCCTCCTGGAGCCCTGGTGCCGGTGTGCCTTGGGTGACTTCTGTGCTCCTTGCTT

TTCTGCGCAGTGTGAAACGGGGGCCGATCGGACCACCTTCATTCAGCATCACATTAGTCA

GAGTAATTTATGCTCGTGTTTTCACCCTAGTCTCCCAGTTTGGGATAACAAGCTCTTTTC

CCTTCATAAGATACTTATTTAAATCTCGGAACCTTACACTCCTCAAAGGAACTATGTATT

TTGGCCAATAAATGTGATTATTTAACTGAAACTTTTTGAGAAAGGGTCTCATGTAGTCCT

GGTTATATAATGGCCAGATATGTAAGATATAAATAAACTTTTAAATGTATAAAAGACATA

GGAACTTTTAGTCTTTTTGTTTTTAACTGTGTCCAAAGGTTAATGAAATTTCTCTCTACT

GAAAAAGTTAAATACACTCCAACACTATAGGGGTTTCCTACAGAAATGAGTAGCTGCATG

ACTATATACAGTCTTGCCTAGAATTATAGCATGTTATCAAAAAACACATAGGAATTCTGG

CCAAATCTTAATGAGAATTTAATTTCTCATGTTATTAAGTAAGTATTGGGTTATAAAATT

GAGCCAGGTACTCTACTGGGATGCTTGCAAATGCCCTGTCCCATTTAGATAGACTTTTAA

AAATACTTAAAAAATATTTAATTTTGAAAGGTGTGTGTGTGAGAGAGAGAGAGAGAGGGA

GGGAGAGAGAGAGAGAGAGAGAGAGAGAGAGAGAGAGAGAGAATATGAATGAATCCTATG

ATATTTGGGTGCTCTTGAAGCTGGCGTTACAGTGGCTGTGCACTGCCTGGTGTGAGAGCT

GGGAACTCAATTCAGTTCCTCTGAAAGAGCAAAAAGTGATCTTAATAGCTGAGCCAGCTC

TCCAATTTCATTTAGCTAGACTTTTAAAAAATGAAACAAAAAATTGAACTCTTGCCATTG

AGAGGCTTTTAGAGCAGGGTTAGAACATTGATGGGGAAACAAATCTGAAATATCTGAAAA

TTTAAAGTATTTTTACTTTATTAAACTGATGAATTTCATAAACAAAAGCAATTTTTGTTT

TGTTTTGTTTTGAGACAGGATCTCATGCTGTGTTCCAGTATATTCTTGAATTTGTGATAA

TCCTTCCTTAGCCTCCGAATGCTGGAATTATAGTTGTGTGCCAAAATCAACTTATCCAAT

CTCAATTCTTTGAGAATAGATAAACACATTGGATATGGTTTCAGTATATCAGTGCATACA

TTTAAACATAGAGATGTTAAGAAAAAATGTCTCATGTGTAACACAGTGCTCATAAATGAA

CAGCTTAAGGGGTTCCGTTAATAATTAACTTTAGTTCAGTGGTGTAACACTGTATCATTG

GAGAGTCCTCTGGGAGGTTATTACCTGTATCTTCATTCCCTGCCCACAAACAGAAAAGAA

ATGCATGAAGTTTTTAAAATGTACTTTTTACTATGACGACTGTATAGTAAGGACTGTTGT

TTTCTGGTGATGGAAGAGAAGGAAGAAGGAAATCTCAGGGAATGTTAGTGGGCATTTGTT

TCTGTTTCTGTTGAGACTGGGTCTCACCATGTAGAGTAAGGCTGGTTTTCAGAACAGAAA

ATTGTCTCAATTTACTTCTCTGATAGTGCAGTTTTATTTTATTTTGCTTGGAAATGTCTT

ACTTTTCTATTGCCACGACAAAACACTATGAGCAAGGAGTTTATTTTTCTGTTGGTTTGA

GTATATCTGCACATCTATATGTCAGAAAATGACCTCAGCTCCTGCTCTAGATGGCTGTGA

GCCCCAATGTGGTTGCTGGGAATGGAATTTAGAACCTCTAAAAGAGCAACTAGCGCCCCT

AACTATGTTTTGAGCCCTGATCAAGGCAGTTTATAAAAGAAAGCATTTAATTGGGGTCAT

GGCTCCAGAGGGTTAGAGTCCATGACCATCATGGTGAGGAGCACTGCAGCAGGCACACAC

ACATGGTGGTAGCTCAGAACCTACATCTCATCCACAAGCATTAGTCAGAGAGACCTAACA

GAATGATGTGGGGTTTTGAAACCTCAAAGCCTAATTCCAGTGACATACCTGGTTTACAAC

AAAACCATGCCTCCCAAGCCAAATGTTTCTCAACCTGTGGGTCATGACTCCTTTGGGGGT

CAAACAACCCTTTCGCAGGGGTTAAATATCAGATATCCTGCATATCAGATATTTAAATTA

TGATTCATAACAGCAACATTACAGTTACGAAGTAGCAACAAAAATAATTTTCTGGCTGGG

GGAATCACCATAACATGAGGCGCTGTACTAATGTGTCATAGCATTAGGGTCAAGATCCAC

TGTCCTTGCCAAATAGTTTAAACCAACTATAGACCAAACATTAAAATATCACAGCTTATG

GAGGCCATTCTCATTCAAACCACCAAAAATGTTTTTAGAGAATTCTTTTTCTTCAATGAG

TAACAAGATCAAACCCAGGGTCTGTATATACTAGGCAAACACTCCAGCCCTAAACTATAG

TTTCAGAAGTTTGATAGGATGAAAGCCCTCAGCTTTGGAATTAGGACAACTTTATCATAT

ATAAAGACACATGCTACTCCTTGTGGATACTCTGGACACTTTACTTTTATGATTAGTGTG

GGTGTATGTATGGTGTTCACATGTCTGTGCTGTATGCTCTTTTGTGTGTCTTTATGCTGA

AGCCAGAGCAAGATACCAAGTGCTATCTATTGCTATGTGCTGAATTGCCTTGAGACAGGA

TCTCTCATTGAGCTGGAAGCTACCATTTGAGCCAGACTTGAAAATCTTTGGGTGAATGGC

ATGCACACTCGTGCCTCGATGTTTAGGTGGGTGCTCAGAATTTAAACTCCAGTCCTTAGG

TTTTTATTCTTTTAGAACAAGCATTCTTACCCACTAAGCTATCTTCATAGCCTACAATGC

TTTTATTTGATTGTTTTGAGGCAAAGTGTCATATTGCCCAGGTTAGACTTAAACTTCTTG

TGTAGCTGAGAGCTGCCTTGAGCTATCAGTCCTGTTTCTACTTCTCAGGTGCTGGGTAGA

TGCTGTATTATTAAAGTTGCCAAAGGGATTAAATTTGGTTGTATTTCTTCACTTAAGTAT

TGTTTAAAGAAAAATTTTAAAAAAATTTATGTGTGTGGGTATTTTGCTTGCATGTTTATC

TATGCACCACATGCATGACTGCTATCATTAGAGGTCAGAAGTAGTAGTTCAGTGCCCTGA

AACTAGAGTTACAGATAGTTGTGAGTCACATGTGGGTGTTGTCAATAGAACCTAGGTCTT

CTGCAAGACCAACATGTACTTTTAACTACTGAGCTATCTCTCCAGCCCGTTAGGTATTTT

CTTCAGATATAAAAGCTAGAACCAAACTAGATAAGTATAATGTTTAAATTCTTCTCTGGG

AATACTGGCTAATTCAAGTATCTGTTTCCCTATATTCATGTTTTATATATTTGTATTTTA

TCCATTCATTCATCCACCCATCCATCTATGGTTTTCCGAGGCAGGGATTCTCTCTGTAGC

CCTGGCTGTCCTGGAACTAATTCAGTAGATCAGGCTGGTCTCAAACTCAGAGAGCCACCT

GTTTCTGTCTCCCAAGTGCTAGGATTAAAGGTATGGTCTACTACTGCCCAGCTTTTATTT

TTATAAATTTTATTTTATGTCTATGGACATTTTACCTGCATATGCCTATGCCCTTTGGTG

AGTGAGTGTGTGTGTGTGTGTGTGTGTGTGTGTGTGTGTGTGGTAGAGAGTGATAGCAGA

TGCCAGGTGTTCTTACCACATTGCACTATATTAGATCTATAGTTTTTTTTGTTGTTGTGC

TAATTGTAGTTTTTAATTCTTTTTGCTTTCAGTACTTTCTTGCAAAGCATAATATTAGTG

TATGACTTAAAGATTCTATTTTTGGTATTTAAAGGGAATAAAGACCTAAAACTTTGACTG

TTTTATACATGTTTTATATAGTACTTTTGATTTTTATAATGCTTTTTCCAGTATTTTATA

TTTAGCTTTACCTATAGTATACAGTCCCAAAGTAGAATTTTAAAAGTTATTATTATTATT

ATGATGATGATGATGATGATGTGTATGTGTGTGTATCTTCATGTGGGTTTATGCATGTGA

GTGCAGGTGGCAGAGGATGCCGGTGCCATCAGATGTACTGTAGATGGACAAACAGATGGC

TTTGGATTTTGAACTTGGGTCCTCTGGAAGAACAGCAAGTACTCTTAACTGTTGAGCTAT

CTCTCTAGCCCCCAAAAGTATATATCCTAGAGGGAAAATTATTTTCAAAATATTTATCTT

AGTTTTTAGAGGTATTGCTCTCTTTTTAAATGAGTTGACTATCTAATGTACTGTTTAACA

CAGTTCTAAACTGCAACAAATGTGTCTAGCATAATGAAGCCTGATGATAAGCACAGCTCA

CCAGTTAAGCAAACACTGCTATACTGCTGCCAAGCCTTTGACATTTTGATGATGGAAATT

GACTAGTGTTTATATATATATATATAATATATATATATATATATATATATATATATATAT

ATATATATATATATATATTAGAGGTAGAATTTTACAGTGTAGCTCTAAGCTAGCCTTAAG

CTCACAGTGAATGTGGCTGGAGAGAGATATTAAGATAAGTGGCTCAGAACACACAGCTTG

CAAGTGGTGGAACTTTAGTTCCTACTCCATCAATTGACAATGCTCTTTACCCACCTCTAT

CACCATATTGTACTTGTCAGCCACATTTCTACCTCAGGAGCTGAGGACAAAGATATTAGG

TATGAAGAGAGGACTGCTAGGGTGGCTGAGTTAGTAGATCTGTCATTCATTGAAATAGGA

GTACAGGCAGGTTTGGTGTAACTTTCAGCATTTTGTTTAAGATCTTTGAGGCAACCGCTA

AGAAACACCTGGCAGGTTCTTGGGAGGGTGGTGTACAGGAGGCCTATAGCCTGAAGTGAA

CATTGAGAATCATCAACTAAAGATGAAGTGGCCTAGGGAAAGGATGGAGCATTGTTGTTC

CTGGAGGCCTGGAGAACGCTAGCCTTTTTTGTTTTGCATTTTGAGACAGAGTCTCACTAT

ATAGCTCAGGCTTGCCTAAAACTTGCTATTTAGACCAGGCAAGCATTGAACTCATAGAGT

TCAGCCTGCCTCTGTCTTCTTAGTTTTAGAATTGATGGCATGTACCACCACACCCAGCTA

GAATGCCAACTTTATATGAGTAGATTAGCACATGTGGTTTAAATAGGCTCATTCCTGTAA

TCCCAGCAGCAATGAAAAGGTTGAGGCAGAGCCGGGCAGTGGTGGCACATGCCTTTAATC

CCAGCACTTGGGAGGCAGCGGCAGGTGGATTTCTGAGTTTGAGGCTAGCCTGGTCTACAG

AGTTAGTTCCAGGACAGCCAGGGCTACACAGGAGAGAAACCCCGTCTCAAAAAACAAAAA

CAACAACAACAACAACAAAAAAAAACAAAACAAAACAAAAATAAAAAAAAAGCTGAGGCA

GGAGGATCACTATGAGTTTGAAAGTAGGCTGGGTTACAGAGCGAGATCTTGTTTCAAGTG

TTTATTACATTTTGTGTATAGGCATTTTGTGTGTATTTTTACATATCATGTGCATGTTTG

GCACATTCAGAGGCTTCTGAGGGCATCAGATTTTCTGGGACTGGAGTTATGGATGGATGG

TTGTGAACCACCATGTGGGTATTGGAAATGAAGTCCTGGAAGAGCAGCCAGTGCTCTTAG

CCACCAAGCCATCTGTCCACCCCAAGATCCTGTTTCAAAAACAAAAAGTCAAGCGAGAAC

TGATTAATTTGGAAGAAGGTAGTGTTACAAATACTTCAAGAGGTTAAAATTTGAAGTAGG

AAATGTTGAGGAGACATTAAGGAAAATGGAGCCTAAAAAGATAGACTTGACTAGTTGGCA

GTCAGTCAGGGCCAGGGATAGCATGGGGGGGGGGTGGAGGAGGAGAGGGGAGGAAAGAGA

GAAACAGAGAGACCTGAGAGGAGTGAAACATTTCTCTGATAATTCCGAGCCAGAATCTAG

TGTAGTTGGTTATTGAGGCTGCTCTTTTGACATGCTTGTGATGCCAGGAAATAGGTACTT

TCAGTGATGACTGCCTCTAGGAAGAGGAAGCTGGTAGATGTTCCTGAATTTCATTGACTA

CCTTTTCATCAGTTTTTTTTTTCTTTTACTTTGTCATGATTTCTTTGTTCTTTTGAGAAT

GAATCTCACAGTATAGTCTTTGCTGGCTTGAAACTTATAGTTAGACCAGAGTAGCTTTGC

ACTCACAGAGATCTGTCAGCCTCTACCTCATAAGTGCTGAGATTTTTATTTACTTTTTCT

CCTTTACTTTAAAAGCATTAATTCTTAAGAATATATAAAATGGTGGCACATATCTTTAAT

CTCAGCATATTGGAGGAGGGGATCTCTGTAAATTCAAGTCCAGGTGGTCTGTATAGCAAA

CTCTAGGTCAGCAGCCTACACAGTGAGACTTTATTTCAACAACAAACAACAACAGAATGT

ATTAGCTAATCAGGACAGGCAAGATGGTGTAGTGGGTAAAAGTGCAGGTCTGATGATGAT

CTGAGATTAATTTCTAAGTCTCACAGTGAAAGGAGAAGTTGAGTCCAAAAAGTTGTACTT

TGACATCTACCGGTTTACCCCCTCACACATAAACACAGAATAATGAAAAGATGAAATTTA

AGAACTATATTATTCAATGAATTACAATATTTTATAGTGAAATATAGAAATTACCAGTTT

TTTACTTTATTGTCCTGTTATTGTTATTGCTCAAATATGATCATTCTTGTTTGTTTATAT

ATCTGTTCTCTCTTTAGTGTGCAGAGTCTTGGAGAGTAGAGTGCCTATATTGTAGAGTGT

CTCCAGGACTTTGTGTAATAGAATGACATATAGCAGATTCTGATTAGTATTTAATGAGCT

GATGGTCCCTATCCTTTCTCTTGGTTATAGGACTTTTAAAGACAATAAATGTTTCCTCAT

GGAATGCTACTAGCATCATGTAACAGGGTTAATCTCATGGAATGGCACCTTATCTTTTAA

AATTATTATTATCATCATCATCATTAGTGTGCATGTGCATGCTCTGGGAGTTAAAAGATG

TGTTTATTGATCAGAGGATACTTTGTAGAGTTGGTTCTCTCTCTTTCTCCCTTAATGGGT

TCTGGAGGTTGAAATCAGACCACCATGTTTTTATATAGCCAATACCTTATCAGTTCAGCT

GTCTTGTCAGTTCTTCAGGTTTTTCCCCCAAGATTTATTTATTTTCATTTTATGTGTATA

GTTGTTTTGCCTACGTTCCTGGCATACATCCTGAATTCCTGGCCTTCTTTCTAGCTCTGA

GATGAAATTTTTTACATTTTGATCTTTTTCTTCACCAGTTTTACCTTATTTCAAAGTGTA

CGCATATCTGTCTTTGTACCATAAGCATGCAGTACCTGGGGGAAGTCCAGGAGAGGGCAT

TGGCTTCTCTGGAACTAAAATTACAGGTGTTAGCCACTGTGTAGGTGCTGGAAACCAAGC

CTAGGTCCTTTAGGAGAGCTTAACTGATGAGCCATCTCCCTTCCCTCCTCTTTTTTTTTT

TTTTTTGTTCTTTAAATTTATTTATTTATTATATGTAAGTACACTGTAGCTGTCTTCAGA

CAATCCAGAAGAGGGCGTCAGATTTCCTTATGAATGGCTGTGAGCCACCATGTGGTTGCT

GAGATTTTAACTCAGGACTTTTGGAAGAGCAGTCAGTGCTCTTAACCACTGAGCCATCTC

ACCAGCCCCCCCTCCTTTCCTTTCCTTTCCTTTCCTTTCCTTTCCTTTCCTTTCCTTTCC

TTTCCTTTCCTTTCCTTTCCTTTCCTTTCCTTTCCTTTCCTTTTCTTTCTTTCTTTCTTT

CTTTCTTTTTTCTTTTTGAAATAGGGTTTCTCTGTGTAGCCCTGGCTGTCCTGGAACTTT

GTAGACCAGATAGGCCTCCAGTGACAGATCTGCCTGCCTGAGCCTTCCAAGTGCTGGAAT

TAAAGGCACCCTACCTCCTTCCCTTCTTTTAATGCAGGGTATCTTATAGCTCAAGCTGGC

CTTGAACTCCATGGGTAGCCAGGGGTGACCAAGGATGACTCCTGATTCCCCTAAGTCAAC

ATCATGAGTTCAGGGATTGAAGGTGTGCCAATGTGTGGTAAGCTTGTTTGTTTGTCTGCA

GTTTTAAATTGTGGCCCACTTAGGAGGGAGTTCTGGGAGCAGTGTGTCCATGGCTAATCC

CTGAATTCCAATCTTTCTTTCCAGGTCTGAGATATAATCTTCTACATTTTGCTTTTGTTT

TTTTATTTACTAGTTTAACCTCATTTCACATTGTTAAATGTTTAGAAATAAAATGGTTAA

AAACTTTTAAAAAAGATGTTTCATGCTGTATACCTTTAATTTCAGTTCTTGGGAGATCTG

CAGAGTTGGGCAGATTTCTGAGTGCAAGGCCAGTTTGGTTAACACTGCGAGTTCCAGCCA

GCCAGAGTTGTATAGTGAAACCTTGTGTCAAAAAAAGAAATTAGTTTTATTTGTGTCTCT

GTGAGTACAGCATATGTGTGCAGTGGATGGTGGAGACCAAAGAGAGAATTAGATCCCCTG

GAGCTGGCGTAGCAGGCAATTGTGAGCCACTCAAACGACTGTCTTTAAATATTGTGCCAT

TTCTTCACACACACACACACACACACACACACACACACACACACACACACACAGACACAG

TGTGTGTGTGTGTGTGTGTGTGTGTGTGTGTTGTTTTCTTAGATGGTCTCATTGTGTAAC

CCTGGGTGATCTGAAATATATAGACCAGGGTGGCCTGGAACTCACAGAGTTTTACCCCCC

TGTACTTCTCAAGTGTTAGGATTAAAGGTATGTGCCACCATATCTGGCATAAATTGATGT

TTGAAGAATCATTTCAGCCTTTAATTCTGGTTTTTAGTTCTAAAGAAGCTAGATCATTTA

CCAGTGGGAGGCAGAAGCATGTGGATCTCTGTGAATTCGATCTAAGCTAGCCAGGGCTGT

ATAGGAAGACCCCATCTCAAAAAAAAATTTTTTTTCCTGGCTTAGCTCTGAGAGATTTAA

TTTTTTAAAGAATAAAATGTTATAACTTCCTAATTCAAAATTGTGATTTTACTGCAG**GAT**

**AAATAAGGAAAACAGGGAAGGAACAAAGCATTGATTACACATGTAATAATAATGAGCAAA**

**TGTGGCAGGAAAAAGTATATGAGGACAAATGTAAG**GTAGGTGGATATGCTTTCCTATTCT

TTGGAAGGTCTTGCCTAGAGCTGAAAATTCCAGTGATAAGCAGATTGGTGGTACCTGTGG

GCACACAGGTCTGCACGTGTGAATGTATATCTGTTGTGATAACATGTACTCAAATCACTT

AGCCATCCAGCTAAGGTCTTCAGGATCTACTGTATGCTCTGCATAGTAGAGTATGGGATG

AAGCTGAAATTGGTGTTAACATTTGGCTTTGCTGAGTGACATCCTCAGCCAAAAGTAGAC

ATACCTGTCTATGTGGATGGCTTGGGGGAATTACAGAATTAGAGGAATCCTCAGGGTCTG

TATTAGTCAGGATTCTCTAGAGTCACAAAACTTATAGGTAGTCTTTATATAGTAAGGGAA

TTTGTTAATGACTTACAGTCTGTAGTCCAACTCCCCAATAATGGTTAGCAGCACCTGGAA

GTCCAAGGATCTACCAGTTCCTCAGTCCCACAAGGCAAGCAGACGAAGAAGAGAATCTTT

CTTCCTTCTTCCAATGTCCTTATGTAGGTCTCCAGCAGAAGGTGTGGCCCTGATTAAAGG

TGTGTACCATCACACCTGGATCTGGGACTTGCTTTGTCCCAGATGACCTTGAACTCAGAG

ACCTCCTTGCCTTAGTCTTCTGGGATTCACAGCTGCTATGCCTCGAGATCTCCATGTCAA

GATCCAGGTCAGAAACTTTTTTCTCCAGTGTTTTGTAAATCAGAAATCTTCAAGTTTTCT

TTTGGCTACCCTCAGAAAATACTCTATTTATCATCAGTCAGATTTGTCTCTAAAGAGGAC

ATCCATTTTCTATGCCTTTTTGGTAAAGCTACTTGAAAATAACACATTTTGGCCCAGGCC

AAGTGTAGCTCAATTGATAAAAGTGCCTGCCTAGCATACATGAAGCCCCAAGTTCTAGCC

TTAGCAGCACATAAACTGGGCATGATAGTATAGGCCTCCAGATATACAGGCAGGAGAATG

AAGAGTTCAAGGTCATCTTAGGCTACATACGAAGTTAGAGACCAGACTGGGCTACCTGAG

ACCTAATATTAAGTGGGGAGGGGGCACTAATGAGTCATTTTTTTCTTATTCAAGGATAAT

TTACTTATTTACCTGCCTAATTTGATTTTATAAGTTGGCAAGACCTCAAACTACAAGCTT

ACTAGTTAATTGAACACTTTAATTCTAGTTTTAAAGCTGTCTTATTTTTTTAGCTAACAT

TTGTGAACTGTGTGCTTCCTTTTCTCTTTTTTGAACCTAACTTTTAAAAATTGTTTCCTG

GGTGTCCCTGATAGTTTTTTTAAAAATGTTTTTCCTTTTTTAATTTTGCCTACATAGTCG

TCAATGTGCTGCCTGTGTGACTCCATCCTCAGCAGCCATCATCATTTCTGCCTGTGTTCT

CAAAGCATTTCATATGTAGCATGCTCCACATAGTTTAACACGTTACTTGTCCTTGGCTTT

GTGGTAGGACTGTGATCTCAGCACTTGGGAGGCCAGGACTCACAAGTTCAAAGCCAGCCT

GGCCTACACAGATCCTTTCTCATAAATAGCACCTTTCACTTCACCCCTATCCATATAAAC

ATCAGATACTGTGATAGAAGTGATAAGAAAAGATGTAATATATGATGTGTTGTGTGTGTG

TGTGTATGTATGTATGTATGTAAGTATGTATGTATATACATCCTGGGGATTGAACTATAC

TATCCTGGGGATCAAACTCAAGTCATCAGGCTTGACAGCAGGCACCAAACAGTTAATTCC

CATAAAATACATTACAGCACTTCCTTTGCCTACTTTGAATTTATTGAATAGAGAATTAAT

GTCAGAGTATTAAGGCTGTACTAAAAAACCTATTTCTTATTAAAACCAATTAAAAAAAGT

AGTAAAGGTGGCTGCCAGAAGTGAATGTCAGAAAGGTAGGTAGAAGCAGTAGTCAATATT

TCTAGATCGTCATTTTTGCCTTAATGGGTTTTTCTTTTTGTCTTAATGGGTTTTTCTTTT

TGTCTTTTAG**GCAAATGACCATGGAAACAGTTGAATCACAGCAGGATCGAAGTGTAACAC**

**GTTCTGTGGCAGAGCATAGCTCTGCTCATATGCAGACTGGTCAAATTTCTGTTCCTACTC**

**TAGCTCAG**GTAGGCCATAGGCAGGTACAATGAAAGTTAAAATGGAAATGTGGATTGTGTG

AAGTTTTTTTTTTTTTTTCAGTTATCTTAAATATAGCCATACTAATTGATTTTACTTTGA

TGAGAAGACATTTTTGCATTGTAACATATACTTGACCCCTGACACAAACTCCTTTGTAGC

TGTAAACTCCCAAGTTATTACTTGACTGGTTTGCTAATTCCAAATATTTACAGTTCTAAT

ATCATAGTTATTACAGCTAGTGGAAATTTTCTGTTTGCCCATTATGTATTTGTCTTGATC

TTTACTTTCCTATCAGTTAGGTTTTAAAATTTTTATTAGTTTCTTTGAGAATTTTATATA

CCATATTTTGATTATATTTACCCTTTTTCCAGCCCCTCCAATTCCAACTCCCTTTTCCCT

ACCTTCCCAATGTTGTGTCCTCTTTCTAGAATTTTTTTTTCTTTTTTGAAACTGAACCTC

ACTGTGTAGTCCTGGCTGGCCTGGAACCCGCCATATAGACCAGCCTGGTCTTGAATCCAC

AGAGATCCACTTACCTCTGCCTCCTGAGTGCTGAGATTAAAGTGAACATCACCAGATCCA

GCCATTTCTACAATCTCTTAGCCTCTGTACCTCCACCAGTTGTGGGTATCTGTCAGTTTA

CACCTATTACATATAGAAACTTATCTGGTCAGGGTTGAGAGCTGCATTAAGTTATGGGTA

CAATGATAAGTCATTAGGAAACAGTTTAATACCATGTCCATTTAACAGCAGAATGGTAGT

AGGCTCTTCCCAAGGTTCTATGGCTTGTCTAGCCACAAGCTTTTGGCATGACAGAGGTGC

TAGGTATGGCTTCATCTTATCCAGCAGGCCTTAAATTTAACCAGAAACTGATTGTTTGCT

TCCATCATCTTCATGCCACTATTGTACTAGTGAATATGTCTTGCAAGGCCAGTGACTGTT

GTAACTTTCACGGTTCACAGCTGGGTAAGATTGATGATTATTTTTCTCCTCTAGAGAATT

CCCTGACTAAGGAAACTTAGAAAGTATTTAATTTGAAGTTTGCTTATAGTTGTAGAGGGC

TATTAGTCCATGATCATCACGGCAGAAAGCATGGGGGCAGGCTGTCAGGCATGGTGCTGA

AGCTGTAGCTGAGTGCTTTATATCCGGATTTTTAGGCAAACACACAGAATATGAGACTTG

CTGATGTAGGCTTTTGAAACCTCAAAGTTCACCATCAGTGACACACCTCCAAGGCCATAC

CTCCTAATCCCTCCTGTTTACTGCTCTAGTTTGCAGCATGAACCTGGGAGTGGGCATGGG

CAGGGCCTAAAACTAAGGTGGACTGAAATCCCTTTCAATAGCCAACTTTATTCAAAGATA

AAATAATTTATATTCTGAGGGTCAGAGTTACCTGAGTAAAGCATACTATTATTACAAGTT

CAGTCTTCACGTGGCAAAAGCATGTTGCTTCATAGACATATATAAACAAATAACAGCAGC

TATACTGAGTAATCTGAAGTGAACTCATCTTGTCTGTTCTTGGGAAGCACATCCCAAGAA

CAGCACATGTTATGGAGGAATAGACATTATAAGAATCTCGTCTTACTTACTTAGAGTCTT

CTCACAAGGTCTCAGAAATCTCACACAGCTTCATTTCATGCACTGTGTTCTCTATCTCCC

CCCCCCCCTAATATCAGGATACACAGATTGGTTTTGATTTGAGATGTTGTAAGATAAATC

TAATTGCCATTTGATGCATGAGTATCCTATAAGCAAGTAATCTATAATATTAGAACAGTG

AAAGCATTGTATATAATACTGTACCACCAAAAACTGAGACCCAAACTATGTCCAGTACTA

TTAGTTACATTATTATGTTGATCAGAAAAATCTAGAGAATAGCTTGAAAAGAGACCCACA

TGATTAGTAGTTCTGGTATCTGGTAAAACATAAGCCTGTATATGCCTTCTAATAGGAATA

CAAATGTTATGCTGTTCTATGCAATTAAGAAGTGATTTAAATCTCAAGGGAAAATTAAAC

ACAGCCCGCTCTCATAGACTCATAACCTCATAGACTCAATAAACTCATAAATGAGTCTAT

TATGGTCCCAATGTCCCAGAGTCATTAGTAAAGTAACTTCTCCTTTGGGTGCATTGGGGC

CTTAATTTGGCCCCAGTTGGGCAGGCTTGACTTTTAAATATGGTCGATACCTTCTTTTGC

CCTTCAGCTGTAGGTTTGCTATCTTTATGGCGATTGCTACTGTTTCCTTGCCCTCTGCTG

TTTTCTGGAGTTGGTGGGTGAATAGGAAGAGGAATCCACAGTGTTTCAGTGGGTCCATCC

GCAATGACAGATGCATACCCACCCCTTCCCAGTTTTCTTCTGGAGAAGAAATGTTGCAAG

TTAATGCATCAGCTTGCTAATTATCTTCTGCAAGTAAACTCAGAGGGGATAGGGTATGCA

AGAGTATGAATCTATTGCTAGAAATAATTCTACAATAGTAAACAGTCTGAAATAATATTT

AACTAACTGTAAAAAAATGAGTCAAAACAGCCAGTTCCATCTGTTGGGCTGAGGAAAAGG

AACTATTAATAGATTAATAAATATCTGGGCCACGGATCCCTCTTATACTTGTAGGTGCGC

CACCAATAAAATTAGTATCTGCTTGTAGAATGGGAGAAGTTTGGACAAGTTTTGTGATTA

CAAATTCAGTTCGTATGAGAAAATCCAGGGTTTGCTAGCTGAATAATGATTTCCTATACT

TCCACAATACTTTGCAAATCAAATTTGAAAATCCAGAGTTGTGAAACATTCTTAAAAAAT

GATTGTTAGTTAAGGGAAATATAATACAGTAAGGACCAAACCCATGTAATTGTTGACATC

TGGTTCTCCCCTGCTTCACGAACTGAGCAGTTAGCATTTAATGGCCCTTATGGTATAAAT

AGATCCATTCCAAAAGCTTATCCTCCCATGCTATAATATCTGTAGGAGACAGTTTAGTGG

GAAAAATAAGTAAGTTTAAAGGCAGTGAAGGATCCTCATGAATCACAAATGCTTCTTTTA

GTTTGGATTCAAATAGCTGTAGTTCTTTTCTTGCTTCTCGAGATAACATGCAAGGACTGA

CCAATGATGCCTCTCCTTCTGGAGTTTTATATATATTGGTTAGTGCAAAATTAGGTAAAC

ATTTTGATTTTGCTGAAATTGCACTGAAAATACCAAGTTCCTGAGTCTGGAAAAGAATTG

GTAAAGGAATTCCATTTTCCTGCTGTGCCCTAACTTGCCTTAATTGTAAAGGTAGCTTTT

TCATAGGTTTTCCTAAATCCTTGTCAGAAAGGAGAGAGAATTCAAGATTAGATTGATTAT

TAAATTGTCCACCATTCAGGGTCATTTTCAAGCAATGGAGGATACATGTCTAAAGGAGGC

TCCAGTAAGGGAGCTGAGGGAAATGGCACAGTTGCTGGTGGATTTGGTCTGACTTTTGAG

CATGAACGGGGAAAGTAAAGCTCACACCTGAGTGAAGCATACAATCCTAAGGACGAGCTG

TGCATGACAGATACATCTCATAGACACAGAGAAACAAATAATAGCCAGGGGAAGGTCTCT

TTTGCCAATTTTAAGGTTCTTGTATTCATTCAAGGAATTGGTCATAAGTATTTTGATAAC

AATCTAGTGACCTAAAATTGAACAGGGATCTTTTCCCCTTGTTATTGTTGCCTCTTCAAT

ATTGTTTCAAACACTGTCCCGTACTTCTTTATCTAAAGTTCCTTCTTCTGGAAACCATAA

ACAATGTACAAGTAATCTAATAGTTGTGATTTTGATACTTGTCTCCTCCTTTCTTTAAGG

AATGATGCAAAAGCTTGACATAAGGCAAAAGTCTTCCCTGGTTTCTGTTCCATTATGCTC

ACTCACTCTTTCTGTGAGGATCCCCACTCTATTATTTCATAGCTGACTTCAGGTTCCTGA

TTCCTTGTACTGCTCTAGCTGACAACATGAACCTGGGAGAGCAGAGCCTAAGAGGTGACC

TAAAGTCTCATGCAATAGCCAACTTTATTAGAGCATTAGATAATCTAAACTCTAAGGGTT

ACGAAGAGTCACTTGAATAAAGTATACAATCACAGGACCAGCGCCACATGGTAAAAACAT

GTTTTTCCATAGAGGCATGTAAAGAAATAATATCCAGTTGCAATGAATAATCTGGAGGGG

GGGTGGGCATGAAAGCACATTCCAAGAACAATCCATATTATGGAAGAATAGAGATTACAA

GAATCTTGTTTTGTTTACTTGGAGTTTTCTCACAAGGTCTCAGAAATTTCCTCACACAGC

TTCATTTATGGACTGTTCTGAATCTTTTTAAACAGTCCACCAACTGGGAATCAAACATTC

AAGCATGAATCTGTGGGGGCCAATCCTTTTTTTTTTTAAAGATTTATTTATTTATTTTTA

TGTATATGAGTTCTCTGTAGCTGTACAGATAGTTGTGAGCCTTCATGTGGTTGTTAGGAA

TTGAATTTCGGACCTCTACTCCCTCTGGTTGGCCCCACTTGCTCCAGTCCAAAGATTTAT

TTATTATTATAAATAAGTAGCTGTCTTCAGACACATCAGAAGAGGGCATCCATATCTTAT

TTCAGGTGGTTGTGAGCCACCATATGGTTGCTGGGATTTGAACTCAGGACCTTCGGAAGA

GTAGTCAGTGCTCTTAACAGCTGAGCCATCTCACCAGTGCCCAATGGTGGCCATTCTTAT

TCAAGTCACCACAAGGGCTTTTATTACTGTATGATGTCTAGAAGGGGTGTTATTATCTCT

ACTAAAAAGAGAGGTTGTTATTCTGTATCCCAAGATGACTTACAACTCACAATGTAGTTA

GTACAGGCTGACCTTAAACTCAAGGTAATGTTTCTGCCTTAGTCTCTCAACTCTTAGGAT

CCCGAGAATGAGCTACCACATCTGACTTCAGTTAGGTCTTAAACTTTGAAATAGTTTCAT

TCAGAGAATGATCACATACACACACAGACAAAAGAAGTACATTTAAGAATTTATTGGTTT

AGCAGATACTCAACAAAGTTAACAAATTCTTTTTTTTTTTTTTGGTTTTTCAAGACAGGG

TTTCTTTGTGTAGCCCTTGCTGTCCTAGAACTCACTCTGTAGACCAGGCTGGCCTCGAAC

TCAGAAATCCTCCTGCCTCTGCCTCCCTAGTGCTGGCATTAAAGGCGTGCGCCACCAATG

CCCAGCTCAGAAGGTATTCTTTTAAACACTTATATTAAGAATTTGTTAACTTAGCCAGGC

ATGGTGGCACACACCTTTAATCCCAGCACTCGGGTGGCAGAGGTAGGTGGATTTCTGAGT

TCGAGGGCAGCCTGGTCTACAGAGTGAGTTCCAGGACAGCCAGGGCTACACAGAGAAACC

CTGTCTTTAAAACAAACAACAACAACAACAACAAAAAAAAACCAAAAAACCCTACTGAAT

CTTGGCAGAGTTCTAGATATACAGATTTGTAGCTAGCTTCTGGCTTTAAATAAAGCATTT

GGAGTGGTATACTAGTTACTCTTCTGTAGCAGTGTTTTTCATATATATAAATCCCAGCTG

CAAGTTAATTACCTGCCTCCATGCTTAACTTCCTGAATGAAAGACATGCACACTACCTTT

AAAAAAATTTTTTTTATTAGATATTTTCTTCATTTACAATTCAAATGCTATCCCGAAAGT

CCCTTATACTTTACCCCAGGCCTATTCCCCAACTCACCCACTCCTACTTCCTGGCCCTGG

CATTCCCCTGTATTGGGGCATATAATCTTTGCAAGACCAAGGGCCTCTCCTCCCAATGAT

GGCCGATTAGGCCATCTTCTGCTACATATGCAGCTAGAGACACGAGCTCAGGGGGTACTG

GCTAGTTCATATTGTTGTTCCTCCTATAGGGTTGCAGAACCCTTCAGCTCCTTGGGTACT

TTCTCTAGCTCCTCCATTGGGGACCCTGTGTTCCATCCAATAGATGGCTGTGAGCATCCA

CTTCTGTATTTGCCAGGCACTGGCATAGCCTCACAAGAGACAGCTATATCAGGGTCATGT

CAGCAAAATCTTGCTGGCGTATGCAATAGTATCTGTATTTGGTGGTTGATTATGGTATTG

ATCCCCGGAAGGGGCAGTCTCTGGATGGTCCTTCCTTCAGGCTCTGCTCCGAACTTTGTC

TCTGTAACTCCTTCCATGGGTGTTTTGTCCCCCATTCTAAGGAGGGACGAGAAGTAACCA

CACTCTGGTCTTCCTTCTTCTTGAGTTTCTTGTGTTTTGCAAATTGTATCTTGGGTATTC

TAAGTTTCTGGGCTAATATCCACTTATCAGTGAGTACATATCATGTGACTTCTTTTGTGA

TTGTTTTTACCTCACTCAG**GATGATATCCTCCAGGAACACTGTGGAAGAGGAGTCAGAAA**

**GAATATAAGATGACCAGAAGTGTCCTGTAGCTCTGTCCTCCTAATGGAAACATTCTTTCC**

**TAGAGTCTAGAGAGAGGCTCTTAAGTTGAGAAGCTTACAAAATTATGTGACTCAAAAGCT**

**CAGAAAGTTACAAGGCTCTCAAGGTCATATAAGCAATAAACAATTGTCAGAAGAGAGGAT**

**CCTCCATAGAACCAGCCTGCAAGTTGGACAAAGAATTCTAGGGATATAAATTTTAGTGAA**

**TTATCACCC**ATACTGGTGTGGGCTTTTCAGTGATATAGCTTCCTTTAAGTAACCCCAATA

AACTCCATTTGATTTGTTATTCATGCCCTGTCTAAGATGAATAAGCTTTTGTTCAGGAAA

AGTCAAACAACAGGAATAAAAATATGTATTTGTTAATTTGAGTCTGGCTAGTTTTATTTA

ACAAGTTCATCTCCACTTCCATTCATTTTCCTGCATGTCTTAGGGTTACTATTTCTGTAT

TGAAACACCATGACAAAAAGCAACTTGGGGAAGAAAGTGTTTATTTGGCACTATATTTAT

TTACGTTTTAAATGTTATCCCCTTTCCTAGTTTCCCTCCAAAAACCTTCTATCTTTTCCC

CTCCCCCTGCTCACCAACCCACCCACTCCCACTTCCTGGCCCCTATATTGGGGCATAGAA

CCTTCACAGGACCAAGGGCCTCCCCTCCCATTGATGGCCGACTAGGCCATCCTCTGCTAA

ATATGTGGCTAGAGCCATGAGTCCCACCATGTGTTCTCTTTGGTTGGTGGTTTAGTCCCA

GGTAGTTCTGGTGGGATTGCAAGCTTGTACAACCCACTCTGGAAATCAGTCTGGCGGTTC

CTCAGAAAATTGGACATAGTACTACTGGAGGATCCAGCAATACCTCTCCTGGGCATATAT

CCAGAAGATGTTCCAACTGGTAATAAGAACACATGCTCTACTATGTTCATAGCAGCCTTA

TTTATAATAGCCAGAAGCTGGAAAGAACCCAGATGTTTATTGGGGTTACTTAAAGGAAGC

GGTATCACTGAAAAGCCCACACCAGTATGGGTGATAATTCACTAAAATTTATATCCCTAG

AATTCTTTGTCCAACTTGCAGGCTGGTTCTATGGAGGATCCTCTCTTCTGACAATTGTTT

ATTGCTTATATGACCTTGAGAGCCTTGTAACTTTCTGAGCTTTTGAGTCACATAATTTTG

TAAGCTTCTCAATTTAAGAGCCTCTCTCTAGACTCTAGGAAAGAATGTTTCCATTAGGAG

GACAGAGCTACAGGACACTTCTGGTCATCTTATATTCTTTCTGACTCCTCTTCCACAGTG

TTCCTGGAGGATATCATCCTGAGTGAGGTAAAAACAATCACAAAAGAAGTCACATGATAT

GCACTCACTGATAAGTGGATATTAGCCCAGAAACTTAGAATACCCAAGATACAGTTTGCA

AAACACACGCTGCTTTTATCTCTTGATATCCTTTAAACAGTGCAACAGCTGGGCCACTTC

TTAACATCTACCTCCCGCCAATAATCCCGAGATATTACTTACTAAATCTATATTCTACCA

TGGCTGCCCTGGACCCAGAGGGCTGCCCAACTGGGCTGCACTCTCCTGGCTTCTTCACAT

CTGGCTATGCCTCCCTGTCTTCCACCATTCTCAGGCATGGTGTCTCTCCTCTCCTTCTTC

TGGCATGGTGGACCTCCTCCTTCCTCCATCCTCTCTGTCTCAAGCCTGGGAATCCTAAAA

GTCCCGCTTCTATCTGCTCTGCCCAGTCATTGGTTGCTGGCATCTTTATTTACCAATCAG

AACCAACTGGGGGCAGGGTCCCTCAGTTTCTTATGTGCTAGCACTTGGGTAAGCAGTTTT

GGGGACCAAAGTTAACAATTTAATACAAGCATCATTAGGCCAAATCCACTTTCTATGACA

AAATACTATACCAAGTCAAGCTAAGGAAGAAAGATTATTTTGGGTTTATGGTTCTGGAGG

GGTAGAAGTCCACCATAGCAGAGTGGAGGAATTGCTAGCAAGCAGCAGGTGTGGTGGCTG

GAGCAAGATGTTGAGAGTTCATATCTTGAACAAAGCATAAAGCAGAGAGAGTGAACTAGG

AATGGCAGGAGTCTTTAAACTCCCAGAGCCTGCCTCTAGTGATTTACTTCCTCAAGCAAA

ACTACCTCCTAAACGTACCCAAATAGTGCCACCGATGGGGGATAAGTGTTCAAATAAATG

AAACTATGGGAAACATTTTCCTTCAGATCACTGTGAGTAATCAGATAGAACCTCACTGAG

TAATATAGGTGTCTCAATAATTCCAGTACATATGAAGTGCTGTTAAATGCTGTATATAAT

ACAAAACATAAGGCACAAGCAGAGGGAATAGCTAGCTGACTAGGAACTCTGTATGCGATT

TTTTTCATTTGAGGTAGGTACAAGGGTTAGTAGGAGTTTTTTTCATGTGGATGTGGGGAG

GGAGGGAAGTGTATTTTGGGTAGAGGAAATAATGTGTACAAAAGCACAAAGTCAGCATAT

GGAAAATTACAAATGAGCAACATCTTGTCAGTGGGAAACCATTGTCTAGTCTGGGAAGTA

GACCTGGCAAAAGTATGCAGAAAGTTATAGAGAGGTGAGACTCTGTGCTCTAAATATGAT

TGGAATATTTTCAGATGCCCGTTGTGTTTAGAATGCTAGTAGCACTGTAGAGATGGAGTT

GGAAGTCAGTGAGCCAATAGGCAGACCATCATTGGGATTCTAATGGAGCCATGGTGCTGT

GGTATGTAAGGACTACTACAACTTTTCAGAATTAAAAAGAAATAATGTGTGTGTGTGTGT

GTGTGTATCTGTGTGTATGAACATGCACATGTGTCTACAAGTACAATGGAGGTCAGAAGA

AATTGTTGAATCCTTTGGACTGGAGTTAGAGACAGTTGGAATCAAATTTAAGTCCTCTGC

AAAAGCAGCAAACACTCTAAGTTTCCGAACAGCCTCTAGCCACCTGCCCCAGAATCTTAA

AGAGAATGTTTAGATTTTTTAGTTTTTGTGTTTGTGCTGGCACACATAGGACTGGCATAT

TGACTCAGTGGGTAAAGGTACTTGTCATCAAGCTTGATAACCTGAGTTTGATTTCTAGGA

CCCACGTGATGGAGGGAGAAAGCTAGTTTTTAAAGTTGTTCTTTTAATCTCCATCTATAT

GTGTGTCCTGGCACTTGGAGTACTCATACATGTACATACAAATAAGTGGTAAGTATATAA

GTAAGCAGGAAATCAGTAAGGAAATGAAAAGGGAATTGCATAAAGATGTTCATATCTTAA

CATCCAGAGATATTATATATTGACATTTTTGTGCTTATTTATTTATATATTTATTGTCTT

TGAGAATAGAATAAAAGGAAAAGCATATTTTTCCACTTGGGGTGCTTCTGTTTTGTTTTG

TCTGTTTTAAGGCTATAATATCTGCCCTCAAATTCTGCCATTCCTTGTTGAAGTCAAAGT

TGAAAGCAAACCTTCCTTTCCCCACTACTTTCTTTTCTTTAGGAACTGACTAAATTCAGC

TTATGTCATTTCCTCAGAGACATCTGGCCCAGCATGGTTAGCATACTAATGAGTCCCTTC

ATTTTAAGTTGATAATGTGAAGTTTAAAAGGGACCTTCAGGCTGGTGATCTCCAGCTTTG

TAAGCCTCAGTCATACAGATTGCTAAACGGGTCATCACTAAGATGCTTCAAAAGCACCTG

TAACTTATGCAGTTGGGGAGTCAGCTTTTTTTGTTTTGTTTTGTTTTGTTTTGGGAATCA

AAGTTCAATCATCTGCTTGACAGCAGCACCTTTACCCACTAAGCTGTCTCAGTGGCTCCA

AAAGCTGAGCTTTAGTTTAGAGAATTGATAGGTAGAAAGGAGAGGGAGATTAAGTGTATA

AATTCCTTAAGATGGGGTAATGAGCAGAGAAAGGACAGAGATTTGGGAGATGGCTGACTC

AAGGAACTGGAAAAAGCTATAGTTTATGATGGTTATTTCAAAATAGCAAATGAATTCTAC

ATAGTAAGCATATGAATTTTGTTTTCTTTTTTTGTTTTGTTTTTGTTTTTTTGAGACAGG

GTTTCTCTGTAGCCCTGGCTGTCATGGAACTCACTCTGTAGAACAGGCTGGCCTCCAACT

CAGAAATCTGCCTGCCTCTGCCTCCCAAGTGCTAGGCTCAAAGGTGTGTGCCACCACTCC

CTGGTGAATTTTGTTTTCTTATTTTCTGTATTTGATCACAAACCATGGAGCTCTTTCTAC

CATCTTTCTGTGCTACCATAATGTGTATGTAAATGTCCTGGCAGTTGTCAGGAACATCTT

TACTAGGTTGTGCTTCACTGGTTTCTAAATGTAATTAGGAAGCATGGCTACACTGGCAAA

TTTGGAATCACTTCTGATCATTGTGGTTTTCCCAGGCAGGTTAAACAAGCATGGAATAAT

TAGCCCCAGGCTTAATGTGCCATTCAAAGAGCTGGAAAAATGGCAGAGTAAACCACTCTC

ATCTACTAGCTGGTTTTCATTGTATGACAGTTTCAGCTGGCATTATGGACCATGAAAAAC

CAAGACAAAAAAAAAATACACATGTGGGGGAAATCCTTGAATTTTTTAAATTTTGCTCTG

TACAGCATAAATATAAATAAAATGCCTCTGTGGAGTCTGTAAAAAACAAAACAAAACAAA

ACAAAAAACAAAAAACAAAACACAACCAAAAACCATGGAGCCTGGTGTAATGGCACACAC

CTATAATTCCAGCCTCTAGGAGCACTTGGGAGGCTGAGAGAGAGGAGCTAAACTTCCAGA

CTAGCCTTGTCTAGATAGCAGGACCTTGTCTCACAAGATGAACCACAGTTTTTATCTTTC

ATATCCTATTAATGTGAAGAAATGAAAAAGTAGTATGAGATAATTTCTGAATTATTTTAG

TTATGGGTTAGTTAATGCTGTGTTTTGCATGGACCATGACAGTTGGAAGAATTATACTAT

TTTAGCTAAAATCTTATGAGTAGGCTGGGTCGGTGGTTAAGAACTGTTCTTCTAGAGGTT

CTGAGTTCAATTTCCAGCAACCACATGGTGGCTCAGATTCTTGTAGTATCTAAAACTGTT

TGTGTCTGAATATTTGTTGTCACTATACAATATTATGTATGTGTATTAAAAAGTCAAGCT

GAAACATATTTAAATCCTGTTATATGTGTCCAGAGTGACTAATTATTTTCAGTGGTTCCT

GATAGCACAAGCAACTTTGGCATATTGATATCTTGCTTTATTTAAACTTGAGGATGATAA

ACTCTGTGGTAGTTTAGACTTAAGTTTGGCTTTATATGAAGAGTATTTATTTTTTATGAG

TGTGACCAAATCATCATTAAACTGTTGGATTAAACATTGTGACCAGTTGTGTGTGGTGTC

ATTTGTGTTAGTAGCTGTGGCACCCCTGACTTCCAGAGCTTCTGCCAATCAGGTACCCTG

AAGATAATATTTTCAGTAATCTGAAAACTATTGCCCTTCAGGCTCTGTACAGGGAAAGGA

GGGAAGAAACCCACCAGACAAAGGCTGGAGTTTTATATTTCTCTGCTTTATAACTTTCCA

GTTACAGGCAGCACTATTTTCATCTCTGCTCAAGGGTTAAGCTACAGGATAGCCATGTGT

AGTACGCTATGCCTATAATTCCTGTACTTGGGATTTGGAGGTGGGAGGGTTAGGGCTCCC

AAGACCATGCTCTGTTACATAGTGGCTTCAAAGCCAGTCTAGGGCACATGAGAGTCTCTC

CTCAAGAAACAAAAACTAAGCAAGCTAGCTTAGCAGGTAGAGATACTTATGACTGAGCCT

AACATCATGAGTTCCACCCTCAAGACTTACAGGTTAGAAGAGAGCTGACTCCCACTAGTT

GTCTTCTGACCTCTACATGTGTGCTGTGACATATGAATGCACATACACACACACACACAC

ATACACACACACACACACACACACACACACACACACACACACACACAGTTATTTTATGTT

ACTTTATTTAATCGAAACTATTATCTTAAGTGTATGCTCTGATATTTAAGACTAAGATCC

AATCCAGCCCTGAAAAACACTTGTTAATAATCTAGAGGACATATTTCTTAGAAATTTCTT

CTAAGAACAAACTCTTCCAAAAACCACAATGCTAAAAAACAAAATGCTACGTACATATCT

AAATAATTTTAGTAATATTTACATACTCTGGAACTAATGACAATCCTCCTGCCTTAGCCT

CCTACGTGCTAGGATCGAGGCATGACCTACCGTACTCAGGTATTTGTAAACACTTATTTT

TGTTTTGAAGTGTGTGTGTGTGTGTGTGTGTGTGTGTGTGTGTGTAAATGTTTTGCCTGC

ATAGTGCCTGTAGAGGTCAGAAGGCAGAGTTGGGTCCCTTAGAATTGGAGTTAAAGTTGT

GAGCAACCATGTGAGTGCTGGGAGTTGAATCTGGCTCCTTTGCAAGAACAAGTGCTCTTA

ACTGATGATTCATCTCTTCAGTTCCTTAACCATTTTTAAAAATTATATATATTTTTATTC

ATCTTGTATGTGCATTGGGGCATATACATAGGTCAGAGGACCCAGTTCTGCAGTGTGAGT

CTTAGGGATTCAGCTCACGTTATTCACTTGCTTGGTGACCAAGTACCTTTACACACTGAA

CCATTTTGCCAGCCATAGTATTTTTTTAATTTACTTTTTTTAAAATTAGTTTACGTATGC

GTGGGAGACATATAAGCTTGCCTGTGTGTGCTTGTTTGGCATCTAGGGGAGGGGAGGACT

ATATTGGTTACAGATACTTCAGGGCCACACCTGGATTTTTACATGGGTACTAGATATCCA

CACACAGCTTCTTCTCATTCTCGAACAGCAAGTATTCTTATCAACTGAGCCATCTTTTCC

ATCCAGTTTTAGCCATTTTTAGGTAAACAGTTGAGTAGTGTTAAATTTAATCCCTATTGT

TGTAAAACTGAACTTTCCATCTTGGAAATTTGAAACTGAACCCATAACAGTTCTTTTTTT

CTTCCGTTTTTTCCTTCCTTTTGGTTCCTATAACCATTCTACTTCTGTTTTCATGAAATT

TAGCTTCTTCCTAGGCATAGAATTGTCTAGGATTTATCATTTTGTGATTGGCTTATTTCA

TTTGTCATGATTTCCTCTAGGCTCATTCATGAAACTTCTTATTCCTTTTAAGGCCATATA

TAATAGTCTATTGTGTATCATTGTTATATTTTGTTATTACTTGGTTTTTTTGTTTGTTTG

TGTTTTGAGACATGGTTTCTTTATGTAGCTCTGACTGTCCTGGAACTTGGTAGACCAGGC

TGGCCTTGAATTTAGAGATCTGTATACCATTGCCTCATGAATGCCGGGATTAAAGGTGTG

TGCCATCATGCCGTACTTTTTTCTCTATTTGTTGTTGTTGTTTTTGTGGTTATTACTATT

ATTATTATTATTATTATTATTATTATTACTATTATTATTTGGCTTTTCAAGACAGGATTT

TTTTGTGCAGTCATGGGTGTCCTGGAACTTACTCTATAGACCATACTGGCCTTGAACTCA

CAGAGATCCACCTGCCTCTGCCTCTAGAATGCTGGGATTAAAGGCATTCACCACCATGGT

CAGCTCAGGACCATTTTTCAAGGTTTCACAAACTTGTTATTTTTGACAGCAACCAACAGG

CACACAGAAAGGAAGGAAGGACACTTAAGATAACCATTACCCCTATGGATGTGATTGAGT

GTTTTGCCTGCTTGTATGTGTGTGTGTGCACCACATTAATGCAGTGTCTGACGAGACCAA

AAGAGGGTGTTAGATACAGTGAATTGGAGTTACAAATAGCTGTTAGCCACAGTATGTATT

TTGGGAAACAAACTTGGGTCTTCTGGAAGAGGAGTAAATGCTATTGACTTCTGAGCCATT

TCTCCAGTCCCAGGATGATCTGTTTTGAAGTTGTTTTTGAGGTAGTTTAGAATATTATCA

AATAATAAAATGAATTAGAATATTAATAATTGCAATTTGACTCCTTCCTAGATGTTCTTG

TAAATAGTTGCTGTAACTTGGGTGAAGTGACACAGGCCTGTAGTCTACTTGGGAGACAGA

GGAAGGAAGATTGCCATGGGTTCAAAATTTGTTCACATTGCTAGTTCTAATCCAGGCTTG

GCTAGAGTGATACCCCACTTCACTCCCCACAAAGGAAAACAAAACCCAGAACAAAACAAT

AACAAAAACTCTACTAGGTATGGTGATGCATGCCTTTAATCCCTGATACCATGTTTAAAA

TTACATTAAAATAGAGCTAGAGATGCATGACATAGCTCACTGGTGTATTGTGACATCACA

AACTCCCACTTACCTAGCAAGTGGGAGAAACTGGGCTCCATCCCTAGCACCTGGGAAGGG

TTTTGCCTAAAATGTTCAATCCCCAGTACCACCAAAAACTTAGAAAGAGAAAGATCAAAT

GATTCTTTATTACTTAATGCTAGTGAGCATTATTAAACGAGTTCTTAGAAATATCAAAAT

AAGATATTTTGCATCTTCAAGTGTTCAATTTATTTGTTTTTTTGAGACAGGGTTTGTTTG

GCTTTGTAACCCTGGCTGTCCTGGAATTCGCTCTGTAGACTAGGCTGGCCTCAAACTCAA

AGAAATATAACTGCCTCTGCCTCCCAAATGCTGCTGGGATTAAAGGAATGTGCTACTATT

GACTGGCCTCAAGTGTTCATTTTATAAGGTTCTATTTTTTCTCTTTTCAGTTTTTAAATA

TTTGAGATATTATTTAACATCGTTTTTCTACAAGATATATAGAAATGGGGCAGTTTTCAA

CAACTTTCTTAGATCATGAAAAGGAATTTTGATTCTAGTCCTTGATCTTAGTCTAGATAA

CACAGATTTGATCTGCAGCAGAGTGGATAGTGTCAGTGTTATGCTTTATTGTGAATTCAC

ATTTCTTTAATCCTTTATTAG**CTGTCCTGATTGCAGGCCCATGTCTGTGATGTTCTTAGC**

**TGTATTGTGACATCACAAACTCCACGATGACGGAACCTAGCTACGCACTGGAGCTGGTCT**

**ACTAGGTGCTGATCGCATGCTGTGCTACAACACTGTGAGGTTTCCCCAGTATAAGTGTCA**

**TTATTAGAACTTTTTTTAAAGTTCCAAATTCAAGTGAGGCTATCAATCGCATCACCTTAC**

**CCTGTTTCTTCAGATTAACTACTGTCTGTAGTGGGTTTTCAGTGGATGTGGTGGCATCAG**

**CAGAACCTATGCTTCAGGCATCCTATAGAAGAAGATTATTCTTCAGGGGATCTGGATAAA**

**AAG**GTAAATTTTTATCTGCTGTGAGTTCATTATATATGTTTTTAAAGCCTCAGAAAAAGA

GTCTCAAACTATAAAGTAGAATACACACCACCTTTATGTTTTTAACTTATTTTTGAGAAG

GATGCCTGTTTGATTTTTGGAATGGGATATATACACTTTAGGCTCTCCCCATGACTTTTA

CTTTCTCCTCATTTAGGATTTCTATGCCAATGTCTCCTCCCCTTTGACACTAGATAATCT

CTTTCCATATTTCTCTGCTGCATTACTTTCTAATTCCTTGTCTTGATTTTCTTTTTGAAT

TGAACACTACCCAAAATATTTGTTTTACCAATTGTCTTTTTTCTTTCTGAATTGGGAATG

TAGGGATTTGCATGTTTTCTGCTTAATTCCCACTGACTGAGCACTGGCTCATGAAACCCT

GAGCAAGTGCTCAGAAAGTACTGTTAACAGAAGAGTCAATTAATTAAAAAATGTGGTATA

TTCTTGTGAATTTTTTATTATAAAATTGTTTTAGGGGCCTGGGAGAGATGGGTCAGGGGT

AAAGGCACTAACAACTGAGTTCAAGTTCCTGATCCACATGGTGGAAGGAGAAAAACAACT

CCTGCAAGCTGTTCTCTGAGCTCCATATGTGCACTTATTCACACATATATATATGTTTAA

AAAATAGTTTTAGGCCAGCCTGAGAGATTGTTCAGCAGTTAAAAGTACTTGTTACTCTCC

CCTAGCCTAAAATACTTTTCTTTGTTTTCTCTTTACTACTTTTTCTCATTGCACTTTGGA

AGATTTCTCAGGTTATAGCATATTTGCAAATCTGCTAATTTCTACTTAGACATTACAAAA

TTAAATTTTATCTACATAGCACTTGTTCTTAGAGATTGTTTCCAGCATCCACATGATGGC

AAAAAAAAAAAAAAAAAAAAAAAAAACCTTCCAGTTCCTTCTGACCTGTAGGTGACCAGT

ACACATGTGATGCACATACCTAAGTGCAGGCAAAACATTCATGTATAAAGTAGCCTAATC

TAAAAATGTTTGTTTTAGGTCGTACACAGTGGCCACATGTCTGTATTCTTAGTACTTAGG

AGGCTGAAATAGAAGAATTGCAAATTTGAGTCCTTCTCATAAACACAAAAGGAACAAACA

AACAAAAAATAAAAGTAGTTTTAGAATAAATCTTGATATATATAAGAGAGATTTCTTTGT

TTCTATCTGCTTATTTTAGTTGAAGTACACCTTATTGTCTTCCTATAAGTAACTTTATTT

TTTGTCTTTTTTAAAATTTTTTGTCTTTTGATTTAACTTTTTAAAAATTTGATTTACTTT

TGAGACATGGTTGTCTTATACAGCTTAGGCTAGTTTTAAACCTGCAGTATACATCTCCCT

CCTTAGTGTTATGAATTTGGGGTGTGCTGCCAAGCCTAGTTTACTAATTCCCCAGTGTTC

CCAAAACATCTTTAGAGCCTTTGCCTTTGTAAGTGATTACATATGCATATGAATTCTTCT

CTTGATATTATTCAAATATGTAATCAGTTATGATTCAGTAGTGTGTTGTTTAGTCTTCAC

AGGTTTGCATACTTTCTGTAGTTTCTCTTGCTGTTGATAACTAGCTTTGTTCCATCAAAG

TCAGGTTGAATACAGGATGGTATCACACTTTACCTATTATAAATTTTGGCTTCCAACACT

CACAAAGTGGCTTGCAACTATGTCTTCTTCTGACCTCCATAAGTAACAAGCATACACATG

GTGCATATGGATATATTGTTGATACTTGTTTTGTGTCCTAATATGTGATCATCTTTTTAG

AAAATTTCTTGGACTTCTGAGAAGAATGTACGTTCTTTAGAGTTTGGATGGAACATTTTG

TAGATAACTGTTGTGTCTGTTTGACTTAAGATATCCTTTAACTCCAATGGGTTTGTTTTG

GTTTTTTGGTGTTTTTTTTTTGGGGGGGGGGCTCATTTTTGGTCAGGATGACCTGTTACT

GGTAAGAGTGAGGTATTGAAGTCACCTACTATGTTATATCACTGTAATGAAGTTAATCTG

TGGTTTTAGATCTAGCAACATTTCTTTTCTGAAATTGAGTGCATCTGTTATGGTCTGCTT

ACATTTAGAATTGGGAATATTCTCTTGGTTGATTGTTCTCATGGTGAATATGAAGTGACC

TTATCTCTTTCTGGCTAGTTTGAGTTTGAAGTTTCTTTAGTTAGACATTAAAATTGCTAT

GCATATTTATTTGTTAACATTTCCTTGGAATAAATTTTTTCATTCTAGTATTTTAAAGTA

GTGTCTATTATTGATGATGAGGTTTATTTCCTAGAAGCAGCAGAAAGATGGGTTCTATTT

TCTAATCCAGTCTAATAGTCTGTGTATATTTATTGGGGGAGTTGAGACCACTAATACTAA

GAATGGTCTCAACTCAATTAAGACCGCTAATATTAAGTGACATATTAATATATCACTGGG

AGATGTATATTAATTATTTGCATATTGTTTTTATGTTGTTTTCTTAGACATCTTTTGAGT

AACTATTGGAATTATTTATTACTTGTGACCTCCTGGGTGTGCTTATCCTTTTCTTCAGAC

TGGAGTATTCTTCTAATACTTTGCATAGAGCTTGCTTAGGGAATCATAAATTCCTTTAAT

CTGTTTTTGTCATGAAAAGATTTTTTTTTCTCCTTCAATTTTAGTAGATAATTTGGTTGT

GTATAATGTTTGGATTGACAATTTAGATCTTTTAGAACTTGGAAGATATTGAAAAGCCAG

AAGTTTTTGTTGAATAATCAGATAATCTGTTTATGGGTCTGCCTGTATAAGTGTCATGAC

CTTTCTGTCTTATAGATGTTGATCCCTTTATTTTGTGTTTTTAATGTCTTAACTATAATA

TTTTGTGGGGAGGTTCTTTTTTGGCCCTGTCTATTTGGAGTTCTGTAAGCCTCTTGTACT

TGGATGGGTATTACTTTTTCTAGGTTTGAGAAATGTTCTTCTCTGATTTTTGTTTTGTTT

TAGAGGCCAAGTCTCACTATGCAATACTAACTGGCCTGGAACTCACAGTGTAGAGCAGGC

TTGCTTTGTGCTCACAAACATCCACCTGCCTCTACCTCCTGGATGCTGGTATTCAAGACT

GTTTGCTATGATGCCCAGCCCCTCTGTAATTTTATTGAAAATATATTCTATGCCTTCAAT

GCGATAGTGTCCTTTTAGTCCCACAATATACAGGTTAGATCTTTTCTTGGTGTCCAACTT

TCTCTTCATATATATTTCAGAATTTCTCAATTGACCTCTGCCTTGTCCTCTAAGCCCAAA

GTCTCTATTTTCCTATGGTCCATTCCATTGGTAAAGCTTTCCACTAAGGTATATGTTTGG

ATTATTGAGTTTTGCATTTTCAGAATTATTTCAGTTGTGCTTTTCTTCAGCAATTCTGTC

TCTGTTAAATTCTGTTTTCACATCTTGGATTGCCTTCTTTACTTCATTCATGTCACTGTT

TGCTCTTGGAGTATACTCATGCCTTCTATGAGCGGTTTGAACATTATTATAATAATTCTT

CTGAATTCTTTGGAAATTCTTCCAAGTCACTGTCATTAGGTTCCATTAGGAACCTCATTC

AATTAGGGAATTTACTTGTGGGGGATCCTTGTCATCTTGGTTTCTGATTTTGTTTGTGCA

CTTGGACTTGTGCATTTAGAGTAGTATGTTGGTTGTGACTTCCTTCCTTCCTTCCTTCCT

TCCTTCCTTCCTTCCTTCCTTCCTTCCTTCCTTCCTTCCTCTTTATGTCTGTCTCTCTGT

CTTTCTTTCATTGTCATCCCTCAGTGGAGGTATTTATAGTATTCAGGAAAGGACTTAGTA

GTAGGTCTGAGAGGCCAGCTTTCTTATGTCTGGCAGGTACAGGAAGAATATGAGCAGCAT

AGTGGGCCTAAGCAGAGGAAGCTGGATCTCCTACTTGCTGCAGGAGGCTGTGCAGGATGG

CTGAGGACTGAAGGGTGGGCCTATATCTGGAGGCAATGACCAGCACTTGGAGTGTGGTTT

CTGGGTGGTGTAGATGGACCAAAGTTTGGGATGGATGCAGGAGGCCACAGGGCCCAGGTT

CCTGGTGAAAGTTGTTCCTGGACAGTGCTGCCATGTGAGCTTTTAAGATGATGAAGTTTA

AGATCATTAACTGTACAATTACAGGCTTAATGCTTATCACTTCCCAGGGTTGTTAAAGCT

AGTGAGCATATAGAATGGCAGTTTTACAGATCTAAGTGCTTTCTTGATTAAAAGGACCCA

TCTAAAGTCTAGCATCAAGGATACACTATTTGTGTTTTAGGAGTTTAATGATGTCGGACA

TAGCAGAGGTATCATTGCAGAATTCACTTAGCTCACCTGTAGGATTTTGAGGTCACAGCC

TGTGTTGGTGGGAACTTACAACATGTTTTAGTTTTTATTTGTTTGTTTCGTTTTCCTGAG

ATGGTCTCACTATGTACTTCTGGCTGGCCTGGAACTCACTATGTAGACCAGGCTGGCCTC

AAATTCACAGAGGTCTGTCTGCCTCCTGAGGGCTGACATGCAAAGGCATGCACCACCATA

CCCAGCCCAGGTAATCTTTAAAAGATAATGATTTTATGTTATTTATTTTGTGTGTATCTG

GGTGTGAGCACATGTATACCATGGTGTATGTAGAAATCAAAGAACAACTGTGAGAGTTGG

TTATGGTCTTCCATGATGGAGTTCCAGGATCTGACACAAGTCATCAATCTTGACAGGGAA

GGGGCTTTACTCACTGAGTTATCTTGTTGGTCTACATCAGGTATTCTTGAAACTAAAACT

TTTGATAGAACTGTTGTTTAATATGGAGTTATGAGAATTATACTTGAAAGATACGCAACT

AAAAGCATGTTCTATAACTATTTGTATAATATGTATATACATACATATATATGATATATA

CTCACAGCTATATAAATATATACCTAGTATAATTATATATATATATACATATATATACAT

ATATATATATATATATATATATAAATGTTATAATATAATATACTATATATATATAAGCTA

GTTTTATGTCAACTTGACACAACCTGAAATCATCTGAGTGGAGGGAGCCTCTATTAAGAT

TAAAGCCTCCATAAGATGAGCCCGTAGGCAAACCTGTGCAACATTTTCTTAATTAGTGAT

TGATGTGCTAGGGCCTATCCCATTATGAGTGGTACCACTCCTCGTGGTCCTGGGTTCTCT

AAGAAAGTAGGCTGAGCAAGCCACGAAGAGCAAACTAGTAAGCAGCACCCCTCCATTGTC

TCTGCATTAGCTCTTGTCTCTAGCTTCCTGTCTTCACTTCCCTCAGTGTTGGATTCTTAC

TTGGTAGTGTAAGCTGAAACAAAGATTGATGACTCTTTCCTCTGCAAGTTGCTTTGGTTA

TCGTGTTCCATCATACCAATAGTAACCCTAACTAGGACCGTGTGTGTGTGTGTGTGTGTG

TGTGTGTGTGTGTGTGTGTATTCACATATGCATATGTTATGATTTATTCTTTTTTATAAA

GTTTTCTTATAATTTATTTATTTTCGGTGAACTGATGTTTTTCTTCCATATGTCTGTGTG

AGGGTGTCAGGTCCCCCGGAACCAGAGTTACAGACAGTTGTGAGCTGCCATGTGGGTGCT

GGGAATTGAACCTAAGCCTTCTGGAAGAACAGTAGGTGTTCTTAACCATTGAGCAATCTC

TCCAGCCCCATGATTTATTCTTGCTTTAGTTCTTCAAATAATGCGACTTTGAAGCTGCAG

TGTTTATTCACAGGAAGTGAGTACACAGATTGCAGATTCTTCATAACATAGCACAACTTT

TGAGAATATTTCTAGTATTCTTGAATTTCTCTATTATAGTTTCAAGTTTCTTATCTTTAA

ATGTTTTACTTTTTGTTGTTAAAAAAGCAAATCATTGAGATATACTGGGGCTCTGGCAGG

GCTTGTCAGAGAAGTCTGATTCTATAGGGAAAAGTCCTTTCATATGAACAACTTTCATAG

TTTTTTCCACCACCAGCTTGATTAAGTGTTGTGCTAAAGGATGCCAGTTGGTAACATGTA

GAGTTTGAGACCCCTATTTAGAAAGAAAGAAAGAAAGAAAGAAAGAAAGAAAGAAAGGAA

AGAAGAAAGGAAAGAAAGGAAGAAAGGAAGGAAGGAAGGAACGAAGGAAGAAAGAAAGAA

GAAAGAAAGAAAGAAAGAAAGAAAGAAAGAAAGAAAGAAAGAAAGAAAGAAAGAAAGAAA

ATAAAGGGGAAGGTGAAGGAAGGGGAAGGGGAAAGGCAAAAGGGAAGGAAGGGAAAGGAA

ACACAAAAGCAAATGTGGGGTAAGAACACCTGCAGAGGGTGTGGCATCCTCTTATGGGAA

CATGCTAGCCAGTACTTCTTTTTGAGTTAAGGAAGGTTTGTTTTGCCTTTCTATATAAGA

TAAAAAGCATAATTTATATTTTGCTAGTCTCTGATCTACAAATGTCAGTGTTACTGGGTT

TGAATTATGACTATGAAACTGTATTAGAGCTGAAATCTCAGCCTGTCTCAGCCCTCTGTT

TGAGCTGAACAGCAGACTGAAGCAATGTTATTATTGAGCAGCCACTGAACAGTGTTCTAT

GCACTGCAGAACCTTGGGAAAGATGTGCAGATGCTTGCAAAGGATTTGCTAAGATAAATA

TATAGAGTAACAGTGTATTTTATATATAATACAATTTTATTTACCTTTTACTCTAGTAAC

ATTTGTACTGGTGTGCTCAAAACAATAAGTGGAAAATAAACTTTTGTTCTTAACTTGAGC

CATATTAGGGGCATCAAGGACCATAATTAACCGGCTTTTATATGCTTCACTACCTTTCCT

TTGAAGTTAAACACAGCAGCCAGTTTCATTGGAGAATATTTTGGATGAACCAGTAAAAGC

TGCATAACAACATGACCCTTGAGTACATACTACCTTAATTTAATCCTCTTTGTTTTGAAA

TGGGATGTGTTTTTGAGGGGAGGTGGTCCACATTCGCTCCACCCACTGAGCCAGTGCTTC

CCTACAAATATTTCAGGATGTTGTATAATATTGAGTCAGCTAAAGATACATTCAAAGCCT

ACAGATGTCATTGGTACAGATTTGGGAACAACCATTTGTGAGTTTCGGAACTATGTCAAA

GAATATTCTTGCTTGCCTGTAAAAACTGCAGTAAACTCCCCTTGCTCTATAAAATCCATT

GTCTATGAGATGCAGATTGTTTTTCACGTATCTACTCATGAGACACAAGATCCAGACTTT

CTTCACATATGACCTCATGAAACACACTGCAAATTAATTGTAGAAGGATATATTAGAATC

TAGATCTGTTTAATTGAAACAAGTCATTAAAGCCAAGTATAGTAGAGCAGACTTAAAATC

TTAGTCACTTGACTGAGACAGAAGTCATCATGAATTTGAGACCACCCTGGAATATATAGT

GAGCCTGTGATAAGTGAGAACCTTTAAGGCAGGCAGATCTCTGTGTGTTCAAGGTCAACC

TGGTCTGTGTAACTTCCAGGCTAGCCAAGGATACACAGAGAAATCCTGTCTTAAAAAACA

AGACAAAACAAAAAACAAAAGAAAGAAAGGAAGGAAGGAAGAAAGAGAGAGGAAACCAAA

AACCAAACAACAACAAAAATCATAGTCATTAAAAGAAGTAGTTAGTCTGTTATGGTGATG

GTGGTACATGCCTTTAATCTCAGCAGAAGCAGGTGGCTCTCTGTTCGTTTGAGGCCAGCC

TGGTATATGTAGTAAGTTCCAGGCCAGCCACGAGACAACATGAGACAACATAATGAGATG

TTGTCTCAAAACAACAACAAAAACTCACAACCAACTGAAAACACATATACATAAAAACCC

CAGAGTAAAACACTGTCAGAATTTTCACTAAATAGTTTTGTTTTGAAAAATGTAACTTTT

TCATAAAATTATAGCATTGCAGTAGATTTAGCATTGCTGAGCTATTAGGTGATTATTCCA

TTTTAATTTCTAGTGTGGTATTAATCAGTAGATAAAAATCTTCTGCACTGTATTATATGG

GTTTGCAAGAACCAGATATGTACATCTTTATCTAATTTTGTGTCCATCATCCCTATTTGC

AGCCTGAAACAAGTCATGATCCATAATGTATTGTTTATGTCACAAAAATAAACAAAAACT

GTACATCAGGATTCCACTTCTTAGGATTTGGTTATTCAATATTTACCCATCACTCTACCT

AGGTAGAAATCCAGTTGATTCAGATACTGAAACCTCTTTAGGGCTGGTGAGGTGGCTTAG

ATGCTTGCTTACTACTGAACTTGATGACCTGAGTTTGATCCTGTGGAACTACATGGTAGA

AGGAGAGACCAACTCCAATAAGTTGTCCTTTCAACCTTTCAACCTCACACAACACAGTGG

CATGAACACACAAAGTGGCATATATACATACACAGACTTACTCTATATGTAATAAAAATT

TGAAAACAACAACAAACACCTGTGAGATGATAGTTAGGGAGTTTAAGTATAAAGATACCC

TGTGAGAGAGACTGTGAGGTCACTGGAGAGGAGGAGGAACTTTGTGCCAGTCAACACACT

GCTGGATAGCCTTCTATCATTTTTTTCTTGTGCTGACATTTATGGAACAAATCCTTTTTA

TTTGTACCTTTAGCTGCTCAACACACACGCACACACACACACACACACACACACACACAC

AGTACTTTTAATATAGATAGATGTACTTTTCTCCTACAATCCAAATAGATGGTGTTACTG

TAATAGATAGGTACGTTGTCTGTATCCATTGCCTCTGGCCTATTTGTAACCTTCGTAATC

TAGGCTTAGATCAAGGTCTGGCTTCAGCTCTGGCTCCCATAGAAAGCCTTCACTTTGTGT

TCAGGGCTAAACTGATCTTTCTTCTGTACGCCTGTGACATTTATGGCTAGTAAAGTTAGA

GCAGTTAGCTCTTTACTTGTTTGATGTGTTGTCTTTGATTGGGCACCAGTTTTACCAATT

CTACCTACAATTTATACCTCAGTACATGAAGTACAGTGAGTGCTAGACATACGTATCTTT

TTTATGTGTTAAAGTATTTGCTTTCTAGAAAATGACATTGTAAATATATCTTAATTATTT

GTTGTAAATCTGCCTCCCCCCTTCCGCACCAGACTAGGTTTTTTCTGTGGTTTTTATCCT

GGCCACAAATTCATGATCCTTCTGCTTCAGCTTCCTGAGTGTGGAGATTTCAAACATTTC

ACTTTGCAAAGCCATTTTCAAATAGTTTTAAAAGTAATAAAATACTAGTAAAGTAGCCTG

GGACTCACTGGTGTTTCTTAAAAAATCGAGGCTTCTGTGAAAATTAACACCTTGAGGTTC

TTTTATTACTCAAGATAGAAACTCCCACTGCATTTCATTTTCAAGGTCTTTGAGTGGTTT

GAAGAAATCAAAATGCATAGTGAACTGAATAATATTAAGAAAAGTTTCTAAAATTATAGT

GGCCAGAAGGACAGATTACTAGAGACTTTGAGTCCAATCCAAAGGGTGTTTGTGGATACA

CTCAGTAGGGTGATATGGGGAGATGCCTATGTCACTCATCTCTCAGTTCAAGACTGATGG

GGCTCTGCACCTCTCAACTGATACTCCATGTTGGAAAGAGGGCTCCCAAACTAGCAAAAG

GCTTGAAATTTCTTTTTTTTTCCTGTGGTGTATTCTCTCTCTCTCTCTCTCCCTCTCTCT

CCCTCTCTCTCTCTCTCTCTCTCTCCCTCTCTCTCTCATATGTGTGTGTGCGTGTGTGTG

TGTGTGTGAGAGAGAGAGAGATAGTGACTGCAGAAACAAGTATGTACATGTGGTTATAGA

ATATCAAAATGACTTTTTCTTGAACTAATATTGAGGCAGTGTATCAGCTTACATCTAAAA

ATAGTGAAAAAAATTGCCACATGCAAAACATTTCATTTATCAAAAAGTAATCCACCACAT

TTAAATTTTCTTCAGTTCCTCTTAATCAGTTAAAGAGGAGGAGTTTGTGTTTTGTCCTGA

AAAGGGTGACCCTGTTAGTAGCCAAGAGGCTGCATTGAGGTAGTTTAACTGCTTTAGAAC

TTTGTGCTC**GGAACATTCTTAGCTGCCAAAGCATGGGCAGCAAAAGTGAGGCACATGGGT**

**TCTTTAGTATCTGTCACATCAGAGAATGACAGTTCCAGGACAGTGACTACCTCCTAATG**G

TAAGTACCCTACTTGTTCTTCATTTCTTAGATTCAGAAAAAGAAAGAAGTAGGCTGGTTG

TGATAGCAAGTGGTACTCTTAAAAATAAACAAAAGCAAAACAAGTCCAGCTTTAATCCTC

AATGATGGTCATTTTTTCAG**GTTTCTGTAGCTGGATCAGGCACTGGAAGAGGCTCCCCAG**

**CTGTGACTCTAGTACAGTTACCTTCAGGCCAAACTGTACAGGTCCAGGGAGTTATTCAGA**

**CACCACATCCATCGGTTATTCAATCACCACAAATACAAACTGTTCAG**GTTAGCGTCCTTC

CTTGACGATTTGTCTTTGACTTTCTTGAGCTTTAAATTTTTTTCTGTTGTTTAATTCATT

TATCATCATTTTCTTTTACTCTTTTAGAGATAAAGTTTCACTATGTAGCTAAGATTGGCT

TTAAACACCTTTTCCTCCTCCTCAGCCTCTCAAATTATGGGACTACAGGCATGTGGCACT

GCATTCGGCTGTTTTGTGTAACTTTATATTGAAGGTGTCGTCTTGAATACTTGGAGGATT

CCTTCTTATGTATGGTACATTGTGTTGGGCTGTGTATATAGACTGACTTATGGGTAGGAT

TTTTGTTACCGCTCTTAATTTTGTAAAATGAGTAATTGTTAAATAAGCAAGTTCTATATT

TTAGTTTCTTTTTTTTCCAGACATATTTATTTAGCAAAGCTCTCTAGTGGTACTTTGAGG

GTTCCTAGAAAACATTGCTATAAAAATAGTACAGACCTGGTTGGCTTTGGACTCACTATG

TAGCCCAAGTTGGCATCAAACTTCATGCCTGAGCTTCTTGAGTACATCATTGTACTTTGT

CAGAAATTGATTTTATATTAGTGAATGTAACCCTCAGAAATTGAGGATATTTGATGCTGG

GACTAAAACTGCAGATCTTTGTGGATGTTAAGCACATGTTCTAATACCTCCAGAATCCCA

GCCCAAAAGTACAGATTCTTGTCACTTGGGTACTTTATTTTCATGTCACACATTCTTGTA

GTTGTGGTTGTGGTACAGATAAATATTTTATGACTTCTCATCATTGTAATTATCACACAT

GATACGAATTACATCCTAAACTCTTGAAGGTAGTTTTTTTCACTAATAGATATTTATTAT

GTTTTATGTACTTTATAACATACTTTATAGTATTATGTACTTTATAACATACTTTTCATA

TTTACTGAACTGATTTCTGTTTCTTTTTTAGATCAATTTGTTAATCTAATTTTTTTGTTT

GTTTGTTTTTTTCGAGACAGGGTTTCTCTGTATAGTCCTGGCTGTCCTGGAACTCACTCT

GTAGACCAGGCTGGCCTCAAACTCAGAAATTCGCCTGCCTCTGCCTCCCAAGTGCTAGGT

TTAAAGGCATGCGCCACCACCGCCCGGCTTGTTAATCTTTAAGTGTAGAAATTATTTGAA

ATTAAGTGGTGACAAGCAAGGCTCAAATCTGTGAGGAAAATGGATGTGCATATACTTAAT

CTTGCCTTCACATGCATGTGCTTGAGTGTAGTTTCTCACAGAATGCAGTGAGGGCCTCAG

CACAGCACTTACATGATAAATTTCCGTAAATAAATTTTTCTGAGTTTGCTTCTTTTTCCA

GCATAGGTATCTTGTTTTTGTCCCAGGGTGTAGAAACAATTCCTATCATTGTCTTTAGTT

TTCTGTGATGACCCTTGATTACAAGAATTTGTAATTTCTGATTTTAATTATAAAGTAAGA

AAGTTCCCCCTTACTCTTCCTTGTATTATGGATTTTATTGGCAGTAGAAACTTTACCTGG

CTGGTATTGGAAGGCAACATTGTCTTATAATAGGAATACTTTATTATTGCTGTTTTGTTA

AGTGAATTATTTGATTGTCTTTATAGAGATAAATGCTTTTAGATCTTATCTTAGAACTCT

TGTGGTTTAAATGACAAATCTTCTGCATGAACTTCAATTTTTAAAATGTTTTTATTAGCA

TATATTAATTATATGCAATAGTGTTTTCATTATTATGACATTCCATACTGTACTAGAATT

TGCTCTGTAGACCAGGCTGGCCTCAAGCTCACAGACATCCACTTGCTTCTGTCTTCTAAG

TTCTGAGATTAAAGATGTGAGCCACCATTGCTTATCTTGTCTCAGCTTTTCTAAACTGGA

TGAAATAATGGGCTGTACTGCATATAAGGGAATTGCTGCGTTTTGGACAGCTGTGTGAAG

TATATTGCCAGTGTAAGATACATGGTGTATGACCATTGTAAGGCAATAACACTTGTTTCC

TAGTAGATTATGTTGGGTGTGTGAGTGCTTGTGTAAGAGGATCCATGACCAGAGTGTTAT

ATTTGTAGATGCATATATGTATGTAGATACACATGCCTGTCTACATGCATTGAAGACGTT

AGCATTTCTTCTATTAGAATCAGAAATGAATTTTTAGTATATGTTTTAATTGGTAAACAA

TTTTTATCAATTTATTCTGACAACATTTTGCTTTAAAGTTTGTATATATTGTGGTATGAC

TAAATTGGCTAATTAAAATATGTGTTATATAATTTTTACATGCTGGAAACACTTTAAATA

CGCTAATTCTACACGAACTTTTGTAATTTTCAAGAGGAAAATATAATGATATTCTTCTTT

TTGTTCCTCCTCCTCCTCCTCCTTGGCGTTTTTCTAGAGAAGATTTTTGAAGCTCAAGCT

GGTTTCAAGCTCATAGTATATTTAAGACTGTTCTTGAATTTTGTATGCTTCTGATTCTGC

CTTCAAATGTTAGGATTAGAGGAATTCACCACTATTCCAGGTTTTATGTGATGTTGGGGA

TCAAATAATATGGCTTTGCTGTGCATGATGAAAGCACTCTATCAACTGAGTCGCCTCCTC

GGCCCCATACTGGTTTTTTTTTTTTTTTTTTTTTTTTTGAGACAGGGTTTCTCTGTGTAG

CCCTGGCTGTCCTGGAACTCACTCTGTAGACCAGGCTGACCTCGAACTCAGAGACGTTCC

TGCCTCTGCCTCCTGAGTGCTAGGATTAAAGGCGTGTGCCACCACACCTGGCCCCGTGCT

GTTCTTAACTGGACTTGTTTTCAGTCTTGATTCTTAGTTTTTTTTTCCCCCCATATCAAA

GTCTCATGTAACATGAAGTTTTGTAAACCTACCTCCCCTAAACCTTAGAGCTAAATTACT

AAATATATTGACAGAAGACATGTTTAGAGATTTGTCATTTTAGTTTTCTCATTTTCTTTA

ATTGACATATCATTCATTGTATTGTTGCCTGGTTTGAAAACTCTGATACTTTTATTTTTA

TAACTATCTGTCTGTCTGTCTGTCTGTCTATCTATCTACCTACCTACCTATCTATCATCT

ATCCATCCATCCCACCCACCAGTCTTATTTTTTGTGTGTGGATGTGTCTTTGGGTGCATT

CCATAGTGTCTACCTGTAGGATAGTGGGGATTTATTTTTTGTCTGATATGTGGGTCTTGG

GACTGAACTCAGGTTGTCAGGCATAGTGGCAAGTGCTTTTATTCCCTGAGCCATCTTAGT

GGTCCTTAAATATGAATTTGTTTTGACGTCAGAATCTTTACACTCTAATATACTCCTTAT

AATATAAATCCTTCTCTCAACAAGCTTGGTTTTACTTACTTGTTTACATAAGTATTTATT

CTTCTTTGGGCCTGCTTAATGTCCTAACTTTTACACCTAGCTTAAGCCAATGACGATGTA

GAGAGAGCACTAAATTGGGTAGTTTTTCTGCATCTGTTATGGTCTTACTGATATCTGTAC

TTACAAGGACACATTTAGGTTGGGGGTTTGGGATCTTAAGTCTTTTATGCTTTTCCCTTT

CCTGTGAAGTCCTACTTGGTATTCTAAGTACATTTCTATTGAATTTATGTAGAATTTATT

ATCAATTTCATTCCTTGTTTCCCTCTCCACATAAGCACTGAATCCTATCCACAGTCAATC

TTAATCACCATTTTTGAGTGAAGATGGAGATCATGTGTTTTATGATGCATTTCAGAAAGT

CTTGTATTATAG**GTAGCAACAATTGCAGAGACAGATGATTCTGCAGACTCAGAAGTAATT**

**GATTCGCATAAACGTAGAGAAATTCTTTCACGAAGACCCTCATATAG**GTGAGTTAACTAA

CTTTCCAACTTGTATATTTCCACCCAAAAAGACACAATAATTGTAGAGCAAAGCAATGAT

TCTTTATGGCTTTCCTGTTGTCTCTTTCTATATCCTAATTGCTTATATTGTTATATTATA

TTTTAG**AAAAATACTGAATGAACTTTCCTCTGATGTGCCTGGTATTCCCAAGATTGAAGA**

**AGAAAAATCAGAGGAAGAAGGGACACCACCTAACATTGCTACCATGGCAGTACCAACTAG**

**CATATATCAGACTAGCACGGGGCAATACAGTATGTATGCTATGATTCCATAGACACAGTG**

**CTAGCTTTATGTCTTCCCTATTACAGTGAAGGAGTGTGGGCTTGGGCATCCGAATGTTTC**

**ATTAACAGCTGTGCATCGTTAACAGCTGTGCATCACAGAATAAACAGTACATGTTAAAGC**

**AGTAAGATATTGATAAAGCAATAAGTACTGATAACTGAAAAGTCTTTATTGCTGAATATG**

**AATGATAAGTGGTTAAAAATTAAAGGCAGGGTTCCTATTTACATGTTTATATTCATCTCA**

**GGTAACAATTTTAAAAGTTATTGTTTTTGTTGTCTTTATGCTATAAATGTCTGTATTTCA**

**TTGTTGCTGGCATTTGTTGCCAACAACTTATTACACTTAATCATATAAAAGAAGTAAATA**

**TGTTTTCTAAGTTGTGTTTTAAATGTACTTTTGTTTTCATAACTTCGTTAATTTGTGCTG**

**CAGTTTTTCTAAAAGAACAAAATCATTTAACTTTCAAACTGTAGCAATTGGATAGATCAT**

**TTTATTTGAACTTTACACACTGGAAGCTCTAATAAACAGATAGATTGGCATTTAGTCTTG**

**TGCATTTTAATGTATTGCTACTTCTGGCTTTTTTTTTTTTTTTTTTTTTGTTCTTACTAA**

**AGCACTAATCATTTTCCCAAGTCAAGCACATTTGATAAACAAGAATAATGAAATCTAGAA**

**AGCAAAATAAAAGGAAAGAAGGGAAGCCAAGGGAAATAACTGCTTGGGTCCTTGGGGATG**

**TGTGTAATGTTATATACTGTGTCCAAATAAAATTCAAAGACTATATTTTCATTTTGAAAA**

**TACCTGCAAAGCCTCAGGACTTGGCTATTAAAACATGTTATCTCC**TTTTCTGTATGGAAG

ATCTTATGTAGTTATTAGAATTTGTTGTTGTGTGTTTCTTAGAATTTTTCCTGTGGTGAT

AGATATTTGCAATAGCAGATTCTGCGTGTAATTTTAGGTCACTACCATCATGGGTATAAC

TTGTCACACTGCCAGATGATGAATGCACATGTTCAAAATGAGCGTGAAGAAAATTCCCAC

AACCGTTTGTGTCTAGTTTGGCTCATTGGAATTATGTGGTGAATGTTTTAGGACTGTAAA

CCAGAGCTTGCCAGCTTTTGTACCTTAGTAGTACAGTTCTAGGTTCCTGGAGGGCAAAGA

ACTCCTCAGCCCTCTGTCCCTAGACAGACTGAACAGGGCTGGGAGGCTAGAGCCTCTAGA

CTGATCCTCAGCCTTGAGCAATGTTGCCTGCTTACCCAGTGGGCTGTTCATGCTATTGTT

TTCTGTTTGTGCTTTGACAGGGTAAAGGTTGGAAAATACTGCTAAAAGAATGTTAACACT

TGAATTTTGGATTGAGCTAAGAGGCTCAATAACTTGGCATTTCTCTGGAATACCCTGATG

CTCCTTAGGACTTTTCATGGGAGCAGTGATCTATTAACAGGGAACAAGAAAAGTTGATGC

TGTGGGTTCATTTTCTTAGATTATTACCATAAAATGTGACATAGTGAAAAGAAAGCTAAG

AGAATATTGAAGCTGGAGGGGATATGGTAAAGGGCTCTGGGGTCTGTATCTGCTCACTCA

GTGATCAGGTCAAGAAGCATCTCAGGCTTTAGTCTTTGAAGTGATAATTTCAGTGGCCAA

ACTGTAGGGTATATGTGAACACAAAAGAAAAGCATAGGCCAAAGTGGGGAGAAAAAGATG

TGCAAACATTGGGCGAAGACAAAGGAGTTTCGACTAGTGGAGATGGTCTTCTTTGTGCTC

ACTAGGCCTGTCCTTTCATTGAGCTTCAGTACCATAGCTTCTGTACCGTGCTCCCCGCCC

CAGAGTCTACACTTTGCATTGTAGCTCACTATATGTAGGCAGCAAGGTTGGGGAATGAGT

CAGAAGTTCCAGGGTAAATGAGGCAACTGCATTTAGAAGTTTAACTTGTATTTGTTTGCC

CACAAGAATAATTTCTATATGTCACTAGGGAATATTTATATTTACATTATACCTTTATAC

ATACAAGATCCATTTAATTAATCTTACTTCTTAGTATTAAAAATTTAGCATGTTTTGGTG

CTTTGATGTTATATATAAGTATTTAAGAATAAAAAAAGCTGCTAGCATTCTTTGGATTTT

CGAATAGTTTCACTAGAACTATGCATTGTTTTGAGTCACCCTCATAATCTTGTGACAAGA

GTATCAAACTTGATGAAATAAAATATCTCCCACATATAGTTACATTTTTTTTCTTCGTAT

TTATATGTATGCAGATTAGTGTATGTGGATACACTTGCTTACATACAAAGGCTAGAGGCT

GACATAGGTGTCTTCTATGGCTTTTGTACCTTATGGTTTGAGATGAAGTCTCACCTGGAG

CTCACTATTTTTGGCAAGACTTTCTGGCCAGTCCAGGCCCTGGTATTCACTCATCTTCAT

TAACTGCTAAGGTTACAGATGTAGTACACTAACCCCAGCTTTTACATGGGTGGTGGGGAG

TGAACTCAGGTCCTCATGTTTGCACAACTCAGCCATCTCCCTGGCCCAAAGTCATATTTT

TAATATTTGAAAGATATTTGTTGAAAATTGATTTGATTATTTTGATTTCCTATTTTTATT

TTTTGTTAGTTCCAGGGATCCATCTTGAACCTTGTACATGTTAGACAAGTGCTGTATCAC

TGAGCTATATACATCCTCACTTTCTAACTGCTGTTTATCACAGAGTACAGGAAAAATAGG

CAGATTTAAGAATAGAACTTAAAAATGTAAGCATTAAACAGGCAAGGGTTTATTGTTATC

GTACCAAATAATAGCTATACTTGCAGAGACTCTGCTGGGAAAACAGAAACTTGCAGTAGG

TTTGTTGCTAGGGGTATGTCTCCCTAGGAAAGCATTTGCCTGGTGTGTAGGCCTTGGCTT

CAATCTCAAGTGCTATAGCCAATTGGTTTGGTGAAGGAAAATCTGTTATATCTCAGGGGA

ATATCTTGAGATAAGGTACCATATGATGGCATTAGAGATAGTGTTAGCAAAGAACACTAC

TTGCTGCATAAAACATTGAAAATATACAATGAATTCATAAATGCAAAAGTGAAAAGAAAA

TTAAATGTCTTAAAATTCTAAATGGCCATGTGCCTTAATCACTAAGCCATCCATCTTTCT

AGCCCCAAATGATTGAAAATTTTGATTCAGTAAACTCTGTTCTCTTAGGAAAGGTTTTTT

AAACACACATTCTGCTATACCCAATCTTTTCCTTTACCAATAAAAGGCTGGTGCCAAGAC

TTCACATTATTCTTTTAGATGTGCCAGGAGTTTTCTCTGTTCCTATTGTATTTTCCTAAG

AACTTTACTGGAATGAGTCTCTATGACTCACCTTTTTATTCCTGTATATCATAGGTAAGT

GGGTCAAATTAAAATCCACCTTCATAAAATGACAAGTATGGACATTGTGAACTGCTTAAA

GAATTAAAATATTTTATTAGGTGTGGAATGTGATTTGTTATGACAAAGTCATACATGTAC

ATATGTACCATAAGAATTCAGAATCCTTAGGATGCTCATATGAACAAACACTTTGTCATA

AAAGGAATTGGTTTACACAGTCTAAAGAAACTGAGTTTTTATATTCTTAGTAATGACATT

ACTTACATCCCTCAAGTAACTCAAAATTATTTATGTAGTGACATGTAAATGTGGCTCTTT

GATGTTTGTTTTGCTGTTCTAATGGCTACTGTGATTTAATCTGTTCTGTACCTCTGGCTT

TCAAGACAAAAGTATTAGTCTCTCTCCCCATTTCCTCTCCTTCTACCACTCCCTAATGCA

CACACACACACACACACACACACACACACAAACACAGTTCCTGAGTTCCAGGGATAACCA

GAGCTCTACTGTAGTGAGGCCCTGTCTCAAAAGAAAAAAAAAATGTAGTGGAGCTAGGAA

TAGTGGCACAGCAAGCTTTGAATCCCAGCATTCAGGAGACAGATGTAGGTGAGTCTCTGT

GTTTGAGGCCAGCCTGGCCTACATAGCGAAATCTAGGAGAGCCAGGGCTGCACAGGGAAA

CCCTGTGTCAAACAAAAACAAAAAAAATCAACAAACAAAACCCCCAACAACAACAAAATT

AATGTGAGATTTCAGTTTTGAATCTTCTAATTACAGTTTGAAATTAAAATAGTTTGGAAT

TACATCTGCTTAAATATTTACATGCTTATGTATTTGAATGACTTCTATGAAAAACTTTAG

GATTGAAAAGAAACATGCATTAAGTGAAGAGAACTCAGGTCTTACTTTAAGTTTGAATAT

ATTGTGCTCCTTTGCTTGGGAACTTTTCCAGTTTTCAACTTTTGATTTTTGAGACAGGGT

CTCACTATGGAGCTCTAGTTGGAATTCATTATAGACAGGCTGATCCTGAATTCATAGTCA

TCTGCTTAACTCTGTCTCTTTTTCCACAGTGCTGGTATAAAAGGGGTGCAACACTGTGCC

TGGCTAGGTTCCCACTCTTAAAATCCATGTTTTCTGTCGTTATCAGTTATATAAGACCTA

AAATTTGCCTTATGGGCCTGGGGATGTATAACTAAAAATTAGTGGCTTAAGAGATGAGTA

GGTAAGGGAATGAATGAATGACAGAAATATCTCTTCACAGTGCCTATGATACCAGCTGAG

TATGGCAGTGTGTGCCTATGATACCAGCTGAGTATGGCAGTGTGTGCCTATGATACCAGC

TGAGTATGGCAGTGTGTGCTTATAATCTCAGCTGAGTATGGCGGTGTGTGCCTGTGATCA

CAGCTGAGTATGGTAGTGTGTCCCTGTAATCCCAGCATGTAAGAGGTAGAGGCAATAGAA

TTACGAATTCAAGGTCATCTTCTAGTTCTAGGACAACCTGGGGTGCATGAGGACCTACAT

CAAAAACTCGAAACAACAAATACATTTCTTTTCACTGTTATATATGTTGGAAACTTTCTG

TAATAGTTTTGCTATTTTAGGACAAGGTCTCACTGTAGCCTAGACTAGCCTAGAATTCAC

AGCTGTCCTGCCATGCCTCCCGACTGCTAGGATTACAGGCACATGCCACCATGCCTGGCT

AATAAGTTTTGTTTGTTTTTAAAGGCTAAAATAAAATATTATATTTAAATTCATTCAAAT

ATATCTTATTTTCCTACTTTTCCTCTTGATTGATGTTTATTAAAAGCAAATTATTCTATC

CTTGGATAGTTTGGCTTTTTAGAAGTTTCCTGCTTAAATGTATTCCTATAGATAACACAT

TCCCAGAATCTCTGAAACATTTTTATTCTCCAACAATAGTTACCCTTTTGAATGAGAAGG

TCCTCTGTCATTTCATAATGCCCTGCCAGTATAAAGTGTGAAGGGCTAATGTATGAGATT

TATCAGTAGAAATGTTTTCCCATGCCCTGCATGGTGGCATGTGCCAGGGCCAACTGTGCT

GTGGGATGGGTGGGGTGTATATGTGTGGATGCATGTATTTTCAATTTTAATTATATTTTG

AAGAAATATATTTTTCAAATGTATTTTATATTTTAAATATATGACAAAAAATAAGATGAC

AGATACAATATTGTAATGTTTATGTTATGAAAACAAAAACACTTTAGTTGGAAATTTAGA

GAAAAATTACTTGAGAAATACTAAGAAGGAAGAAAGTAAGCCAAAAAAAAATTAGGAAAA

ACAAAAGTAATTTGGTATTTATGTCAGTATTCTAAAAGCTGCTACAAAATTGTATTATTG

AAAATTAAGTTCTGAAGGTTAGGGTGTAGTCCTCCTTAGAGTATGTGCCCCCAAAGCATG

AAGCCCTAGGTTCAATCCTTATCACTTTATCAAACTGGGCATAATTGTGCAGACCTGTAA

TCTCAGCACGAAGTGGGCAAAAGTAGGGCATCCTCGACCAGGGAGTGAGTTCACTATAAA

GATTAGGGATACATGAGACCTTGTCTCAAACAGAAAAGAGAAAATTCAACTTCTGTAACT

GGAATTTGAAACCTTCATTTAATTCACTTAGAATTACAAATTCTTTCTGCCTTCCTCTCT

TCTTCTCTTTTTGTCCCCTCTCCTTCCTTTTATTCTCCTTCCTTCCTTCCTTCCTTCCTT

CCTTCCTTCCTTCCTTCCTTCCTTCCTTCCTTCCTTCCTCCTTCCTTCCTTCGTTCCTTC

CTTCCTTCCTTCCTTCTCCCTTCTTTTCCCTTCCCTCCTTTCTGTGACTGCTTGCAATTT

GCAATTGAGTCCTGAAGCTAGCATCTGATCTGAGCATGCTCTCACCCCCAGCTGCCTTCC

ATAATAAATCAGAAATGTTTAGGTGACCCAAAAGTATAAGCCTAATGTAACTGCAGTGCC

GACTGGAAATCTTACTGACCATTTCCTAAGTATCAGGAGAATGTTGCTTATAACCAACAA

TATTTGAAAGAATAGGTAATAATACTTCAAAGCTGGACTTTCTGTCTTCCTCTTCACTTT

TTCTCCTAAGTAATTTAGTAGAGAAGCCTGGAGAGATGGCTTTGTAGTTAACAACATGGC

TACTCTTTCAGGGGCCACAGACTAGGTTCCCAGCATCTGCATGGTGTCTCACAACCTTGG

AACTCCAGTTCTAAGAGACCCAGTGCCCCCTTTTGGCCTTCACAGACACAAGGATGCATG

TAGTACACAGACATACATGTAGCCAAAACATCCATACATATAAAATAATAAAGGGATGCC

TAGAAGGAAAACTTTGTGGACTCTAGGAATTAAGGAAAATTCTGAGTACAAAATACACTG

AACAGCAAAGCCAAAATAAAATACATTTATGTTGGTAATCTTTTACTATGATAAAGAAGC

TGATGGTTTTCTTATTGATCCCACTTAGAAAGTAACAGTGGAAGCAATGATAGTAAATAG

AATATAATATATTCTCCCAATATGGGATTAAAATAACTTATAATTATCTAATATTTCTTC

TTTTTTTTCTATTAAG**TTGCTATAGCTCAAGGTGGAACAATCCAGATTTCTAACCCAGGA**

**TCTGATGGTGTTCAGGGACTCCAGGCATTAACAATGACAAATTCAGGAGCTCCTCCGCCA**

**GGTGCTACAATTGTACAGTATGCAGCACAATCAGCCGATGGTACACAGCAGTTCTTTGTC**

**CCAGGCAGCCAGGTTGTTGTTCAAGGTATATTTTATTAATCTAATACATTTAGAATACCT**

**TTGTCACACATTTTAGTGTCTACCTGTAGTATTGTGAAATATACTTGGGTAGAATTTTAG**

**GAGGTGTGGGAAATCACTTAAAAAACATTCAAAGAAATGTTTATACTTCAGAGAAGTTAA**

**AGTATGGTTTTCACTGACTCTGAAGAAAGGGTAATTTCAGAGAAGCATGATTAAACAAGG**

**GAAGATGGAAATATAAATGCATTTTTTAAAATTAAGTAAATATATTTCATTAATGTC**ACA

TGCTCTACCTAGTGTATTTTAATTTTTTTTGTTTATGTTGTGCTGTGTGTGCTCATATAT

GAGAATGCACAGTGTTCATGCCATGGCGTGAGGTTAGAGGACAGTTTTGTGGAGTCATTT

ATTTTGGCTTACATTTTATGTTGGTTCTGGAGATCAGATTCAGCTTATCAGTCTTGTGAG

GCAAAAACCCTTTACTGACTGAGCCACCTCACTGGCCTCCAGCACATCTCTATTACTTTT

TATGGGGTATTTAAGAACATAGTCAGTGATATCTTGAGTCTGTTCTTTTTTCCTAGCTTA

TTTTCCAAGTATAACTTTCACACAACTCATTTAAAATAAGTGCTGATTTTTATATCTTTT

AGACTCTTTTTAGTATAAAGGCTAGTAGACTTTTTAAAAAATTTTATTTATTTATTTTAT

GTATCTGAGTATACTGCCGCCATCTTCAAACACACCAGAAGAGGGTGTAGATCCCATTAC

AGATGATTGTGAGCCACCATGTGATTGCTGGAAATTGAACTCAGGACCTCAGGAAAAGTA

GTCAATGCTCTTAACCACTGAGCCATCACTCTAGCCTGACTAGTAGATTCTTATAGCTCT

TATGAAAGGAGTCTATAGTGAAATAAAACACGATGTAGCCATAAGCTTTTAGGGTTACCT

ATATGTTCAGTTCAAACTGGGTAATACATTATTTTAATTTCATTTAGCTGTGCTTATGAT

AAATATAAAACATGTATTTAATACAACTTAAAATCATGTTTTAATTCTAATCTTTTGGGT

CTATTTGTCTAGAATGTATCACACCCACTTAAAGGAAAGTAATATGTTCTTTTATTCTGA

ATTTTCTTGGATATATGGCCCAACTGACCCAGAATTTGAAGAAATTTCCCTGTCTCAGCT

TCCTGAGTGCTGAGCTTACAGCCATGTGTAGTGGCAGGCCTGGTTGATGAATCCTTTTAC

TCCTGTGTACTTGGGGGATGAATGGTGGGTATAATAAGAAAATACAGAGTGTGAAAAGGT

CTGGAGAGATGGCTCTGTTGTTTGGAGAGATGGCTCTGTTGTTCAGAGTGCTTGCTGCAC

AGTCATAAGGACTAGAGTTTAGATCCCAGAACTCAACAAAACAAGTCAGGTGCAGTGCAT

ATGCCTGTAACCACAGCTCCAGAGGGTGGAGACAGGAGGATTTCTGGGGCTTTCTGACTT

CCAACCTAGCTGAGAAAACAGGAACCCCAGGTTAAGGAAGAGATCCTACCTCACAAAATC

AGAGTCAGAGGAGGAGAACTGGCCATGCACACACATATGTATATATACTCAACACAACAT

AGAAACACATATAAACAACAAACTAAACAAGTAAATCTAACCTTAAAATAAGAAGAAAGA

CTGAAGTTGGATATGCTAGTTAGTACATGACAGTAATTCCAGTACTTGAGTCTAAGGATT

ACTTTGAGTTTTAAGCCAGTCTGGACTAAGAGTTCCCAGCCAGCAAGGGTTCTATAGTGA

CACCCTGTTCGTTCCACCAAAACAAACAAGAAAACAAAAACAAAAAGTGGTGGTATTTTA

TGAGGAGAAAATAGTGTTTACACACTAAGGTTTACTTTACATTAAAATTAAAACCATTTG

TTAATTTTTCCTATGCTCAAACCTTGTTTTTCTAAACAAATAAAGAAGAGTCTTTTCTTC

TCATATCACCTGTTTTACATGGAGAAATTGATACTGCAAAATTGATACTTTAAAAATGTT

TTTAGACATTTTCATGTTTGAAACTTTGTACCTAAGTTTTATGCCTTGCGTGCTAAAAAT

CATTTTCTTTTACAAGTTTCAAACATATATTCACTTTTGTGTATGGGGTATACATGTATA

TCAGATACATGTCTGTATCTGGGTGGGTTTCTTTCTTTCAACTGCTGGGATTGCAGGCAT

ATCTGGGCCTGGCTTTTTACATGGATGCTGGGGACCAAACTTGTATCCTGAAGCTTGTGT

GGCAAACACTTTACTGACTAAGTTGTTTTCCAGCCTCCTTTTTGCTTTTTCTGTTTTCTG

TTGTTTCAAGCTGGAAACTCAAAGTTACCCTTCACACACTCTATCCCTTTCTGCCCAAAG

CCCCTGTATAGACTCTTCCCCATGGTTAAGTTAAAAGGTTTTCATTTCTCTCCTATCATC

AGCCTATCTAGGCAGCTGTCCCTGTGGTCTGGATGACCACAAAAGTCAGCCTTCTTGGAG

CTGAGAGCTGTAGCAACATTTCAGTCCTTCCTGGCTCAGTTTTGGGGGAACACTATGCCA

GAGTTCCCTTAAATTCTTGTCATTGGGCTGGAGAGATGGCTCAGTGGTTAAGAGCACTGA

CTGCTTTTCGTGAGTTCAAATCCCAGCAACCACATGATGGCTTACAACCATCTATAATGC

GATCTGATGCCCTCTCCTGGGGTGTCTGAAGACATCTATGTTGTACTTATATATAATAAA

TTAATAAATCTCTAAAACAATTCTTGTCATCACCTTCAAATGATTCTGGGCCTCTAATAC

TTTTTAAAAGGAAGATGTTATCTCCAATATCTCTGCTTCTTTAATACACTCCTGTGTCTG

CAGGTTAACGTGGATGCCCCAATATTCTACCAACATTCCCTAAATCTGTTCAGATTGAGA

TATAATCCTATAGACTGGATTGCTTTCATTATTCATATGTAATATATGTCTATGGTTCTG

GACACTGGGAAGTCGAAGAATCTTAGACTAAGGGTCTAAGGGTCTCTGGAGTGCCTGCTG

ATTTACATAAGCCATTATCTGTCTCTGTACTTGGTGGAAAAGACATGGGGCTCTCTAGGG

GCTCTGTGTTAAGGGCATTAAACCCAATTAATGAAATCTTGTTATGTAACTTTTCCTGGG

TAATGTGAACAAATGTCTGTTAACCCAAGTTGGGGTGGCAGTGGTATATGGTGCTGTGAA

GCTGTGCATTGATTTGGTTGCTTACAGGAGTACATGTGAATGGTCACGTCTAGGAGCATG

GGGATTCAAGTGTAGCTGTATCACAGAAAAGATCACTCCAGCAAAGCTGGCACTCCTTGT

CCATCCTGCAGGCATCTCTGGGGTGGGAGAGTCCCTTCTGCTCAGCACCTATTACTACTT

GTGGAGTAATAACCATGGGCAGAGGCTAAAAAACATTAGTTCCTAGAGCATCCGGGGACA

CTTGTTTACTTGTGAGTCTTAGGGGACTCATCTTCCCACTAGAGGAAGGGAAGGGATATT

TTCATTAGAACAAAATCATTACACAGAAGAACTCTACTCTCAAAATATATTACTTCCAAT

CCTGTTGCATCAGGATTAGGATTTCAACCTAGAAATGAGGAGTTGCGCATGCTTTTGCTC

TGTAGCATAGTGGCTGACTATACTGAGAATGTCAGCAAACACTGGGCATGTGCTAGGCAA

AGTTAGACATCTACTGTGTTACCTACCTCTTCCTCCTGTGTAGGCCTTGCTCTTTCTCAG

GCCTTCCCTCATTCCTCCTGACAGGTAATCCCATTTCTTTCATTTCCTCTGACAGGTTTT

TTTTTCTCTCCACAG**CATTTATATCTACTCCTTGTCCTGACAGGCATCATTTCCTCAGCC**

**TCTACATGTTATTCAGGTTTTATCTTAGATAACCCTTCCTCCAGGAAGACTTCTCTGGCC**

**TTCCAAATCTGGCCTAGGCAACCCCACAGTTCCCAGTGCATCTTCTTAGGTAATAGATCA**

**CGTGTACTGTC**ACTCCGTGTCCTGCCTCAATGCTAAGTTCTATTTGCATTACAGCCTTTA

GTATCTACCAAGATTTGTCATGTGATAAATGTAAACATTACAAATGGAAGTGGTGTAGTG

GCACATGTCTATAATCCCAGCACTTGGAATTATCAGTTTGAAGGCTTCAGAGGGAAGCTC

TGGTCAAATAAGAATAAAAAAGACCCCTATATCTGAAAATATAAGGTCAGATCAACAGTT

TAGTGTAGAGCTGTATTTAAGCTTTCCTAATTGCACTAATCACCTCCACCTTAAAAATGA

CATTTGTCTTTTATGGGTGGACATGGGTATAACATATACACATTAAAGAACAACTTGCAG

GAATTAGTTCTCTCCTTCCAACAGGTAGGTCCTGATGATCAAACTCAGGTTGTCAGGGTT

GGTAACACATGCATTTATCTACTGAGCCATCTCATGCGCCCTTTCCCCTCTTTCATACAG

TATCTATTGACTGTCAATGTGTACATTTAAAGAAATATTGAGATAAAAATAAACATGGAT

TCTGAATTATAAATAAGGATTATTAATATTATTCTGACCCTCAAAGCCTTAGGTGTTCCT

GGATAGGAAGGAGCCCCTGGTGGAGCCTTGTTGAGACTGTCATCAAATCCTCATCTTTAA

TCTTCACTTGCAATTCCAGTTTCATTGCAAGGACTCTGCAAAACCAGTAGAAACATGGAT

GATCTATGCCCTTTGGTCACCTCACCCATGCCACAACATAAAATCCTTACCTTTACTGCA

GCATGCTTAGTTGAATAACATAGAAATACAGCATTTAAAAGAAATCATTGTGTCAGGTGT

GGTGGTGCATACCCTTAATCCCAGCACACAGGAGCAAGGCGGGTGGATCTCTAAGTTCCA

GGCCAGCCAGGGATACATAGTGAGAACCTGCCTCAAAAGAAAAAGTATTGGCCATTTATG

ACATACAAATAGATCATTAATATAAAGTTAGGTTGAGTTTTAATCATACAAGACAGTGGA

TTTTTACAACCCACTGGGGATAGGGGAGAGAAGATCTCATGAAGCAACATGTCAGGACTT

TCTTTTTTTTCCTTTCTTTCTTCAGTAAAGGTGAAAAAATATTCCACACTGTGGAGCCTG

GTATGTCTTAGAACATATGTCAACTTCTTTATGACCTTGATATTGTTAAAATTTCAGTAA

TTGTCCAGCTTTTCATGTGCAGAAATCTCAAGTTATCCAAATAAAAGCTAACATTAAAAA

TATGAATAGCAATTTTAGTTATGAATTTTAAAATCATTAAACTTCAAGGTATTACATTGA

ATCACAAATTTCTATTTATATGGGTCAAAAGGATCAACTATCAGCAAGTTCACATTACTC

TGGTAATTAAGGAAGACACTGTATACCACTTGCTGCTTTGGCATATTGAGTTTCTAGACT

GTCAGCATTTCCTCATTTCAGGTAATGTGAACAAATTCCAAGTCACTCTGGGAACAGCTA

GATTTTATGGTGTGATAGCAAATGGTTTAGCAAATAAAAGTAACTGAATACAGGTACCTG

GATATTTCTACCTCAATAAGTTTAGAAGTGCCTATGAAATACATTTTGTTTTGTATTGAC

TGTCAGGATTTTTTTTTTATTAGATATTTTCTTTATTTACATTTCAAATGTTTCAAACAT

TTCAAACCCTTTCCTGGTTTCCTCTCTGAAAACCTTCTCTCCTATCCCCTTCCCCCGCCC

CCTGCTAACTCCCCCTTCCTGGCCCTGGTATTCCCGTATATTGGGGCATAGCCTTCATAG

GGACTGTCAGGATTTTTTGTTCAGTCCCTGAAATGTGGATCTCCAAACGCTTCCTTCTTC

AGGTTCTAAGAAACAGATAGGGCTCATCTCTCCTCCTGTCTCCACAGCTTCATGAGCTAC

AACTCTGTAACTTCTCATTTTTGCTTTTTGTCAGAGCTCATATGAAAAGATTTTTATGTC

AAAAGTGAGAAACAAGAACATTCGGGTGGTTTATTACAGGAAGAGTTTTTACAGGGCTTT

GCTTTCAGTGAGCTGCACATTGATGGCAGTGATAGGCTGGTGACGTCACTGTGATGTCAG

TGCTCCTACTTATCTTACATATGCTTGCTAGTTTGGAAC**ACTTTATTTTGGACTGTGGTA**

**CGGCCAATAAGACCACTCTATATGCAAAAGCCCAACATGGCTGTAACTGGAGATGAAACT**

**G**GTAAGAACTTAAAGGGGGATTTACATTAATTTTGTTTTGTTTTAATATAGGATTTAGAA

ATTTTTAGGAACTTTTGAGTGTTTTAGAAAGTGTGATTTTTTTTTTTTGTAGTTGCTTAT

GCATAAGAAGTTTCTTTTCATGCTTTTGTTAGAATTAGAGTGCGATTGCTTGTTTATAGT

GCTATTGTATCTTTGCTCCCGGGCTTTAATGCTCTGTTGACTAAGGCAGTGATCTGCCCG

CTCGCCCTGCTTTTCTCTTCCCCTTTCTCCACTCGTATCTTATCCTGGTCGTTCTGCCTC

ACGAAACTAGGCTAATAATTTATCTGCAACCTGTTGGAAATTCAGCCTAGCTACCAGTTA

AAATATTTGATGTCCATGTTGTAAACACTTGAGCTACGAGGTGCACGGGATAGGGGAAAC

AGTGTGCTAAGAATGGATGTTTTTCAGCCTGCTAGTTCTTTCCTCCTGCCTTTAAAAAAT

GGTCTGCTCTTCAGCTAATTGAGTTCAAAGGGCTGACGTCATTACATTTCCTGGAATCGT

GTTTCAGCGGGGAAGT**GGAAAAGTTAAACTCCAAGCACATTGACGTCAGCTCCGAGTCAT**

**GTTGTGATTTAGTCAGTTCCTTTCTGCTTTGTCAAGCTTGGCGGACAGATTGCAAGTTTG**

**TTAACTAGTTCACCACCGCTTTTGGCTGCGGGGACGTCTTTAGATCGAATAACAAGTCTG**

**ACTTTAGCATTTCTCTTGTG**GTAAGTATCTGCTTGAAGATAGGCACGTATTAGGGCACCT

GTAATGCTTGCATTACCTTCGGAGAGGCTCTCCCAGCACCTGCCTAGTGACAAGTGGGCT

ATATTTAGGCCGAGGTTTTGGTGCTTGCTAGATTCATAGACAGAGTGCCCTCTAGTGCCT

TGTAAGGGACGTGTTTGTCTTTCATTGTTTCCTCAGCCTTTTCTACTTAAGCTTTGTAAT

AAGTTCATTAGGTTTTGCTTTTCTTAGTTATAGATAAGTGATTTTGAAATACTGAGTTAC

CAGTGACCTTTGTGTAGTTTGCGACATTGTTTATTCAGTTGGATTTATTTGTAACCCAGT

TTTGAATTTGCAGTTGAATTGAGAATAAAGAGGATTACAAGGCTTCCCCACAATTTAGCT

CACATTGCATTGTTTCTGAGATGTTCCTTAAGAAGCTGTTGTTTTGTCAGATGGTGCTTT

TTGTGAAGTCTGTAGATTGCAGCAGCTATGGAGCAACTGAGGAGCCCCAAACCAGACACT

GTTGTAGTGTGATCCTGAAATCAATGACTTTTTTTTTTTTGGTGCATGCATTTACTTTAG

AAAAAATATTGGTCTGTTTCTTTTAAGCAAACTTTAAATTTCTATTCACCATTTGACTGT

CAGAGCAGGAAAGAAAATAGTAATACAATAAAATAGAATAATTAAAGTATCAAGTATGTT

TAAAGGTGAATAATTAAGGTATTCATTTATATTAGAATTTTAGCCATTGCTTGTGAATTA

TATATTCATATACTTATGTTGATAGAATGAATATGTCATCTTTGAAGCACTTTCTTCTTA

TAGTTTGTTTACCACCACTAGGCTACAGTTTCAAATTTAAAATTTTCAAAAACACTAAAA

CTTATAAATGTTGAACAATATCCAACATTTCCCATAAGACCCAACTGTAGTTTGGAAATA

ACTATTTTTCTGAATCTGTCACTTTTCTCAGTGACATTTGAAAAGATGTTATATTGAAAA

CAATAAATGAAATTTATATCCTTTTTATTAATGTGCAAAACTGCACCCGATTGAAGAACA

TTTTTTGGGACTGTGGCAGAAATGCTCTTAGCAGTCTTTGGATTCTTGGCTCTGGGATAG

TATGGGTTGTCAAGCTGAGGAAATTGGCTTATATCAAGGACTTAGAAACCTTTGATCACC

ACCAGCATTTTATATCATGACATAGTGATATGTGTGTGTCACTTTGAATTTATATAAAAC

AAGTTTCACACAAAACTAACACAAAACACAATTATTTTCTAATTTTCTTTTTTGCTTTAA

ATGTTATCTTGACCCATGAAATTGATTTTATGACCCATGATCTGTAATGTATGCCTGAGA

TCCACACTCAAATCAGGCTTCTTATGTTGATGACTGCCCTAGAAAAGATCTTTATGGCAT

CTCTAATAGGATCATGGAATCAAATAAAAGAATGATATTGTTAATTCTGCTTGGTGTAAT

GTAAATTTGTATTGTCTTTTTTTTGGTCTTATTTATAATTGATTATGTTTGGAACTGAGT

TTACTGTTTTAAGACCTGGATTGTAATAGCTTGTATTCAAGAGGCATTTCAGAATTGTTA

AAACATACTTTTCAACTATTCAGATTTGGGAAATAGTATCTTAGTATTTGATGTAGGAGA

ACAGGGATACATACCACTTTTGTGATTTCTCTTAGGAAAATTTAACGTTAAGGGATGGTT

TTGATTAGGGTTTGACTTTTTAAGTCGACATTAAAGCAAATTATACTTGTATTGGAAGTA

GATAGAAGAATAACCTGCTGTGAGTTTAGTAAGGCCTCAAAGCTCATAGGACAGCTATCA

TGCTGTCTCTACTTGGTGAATATATTTAATGCAGTGTTTGTTATATTTTGCTTTTTCTTA

TCTTATTTTTCTATCTGAACATATTGCCTGTCAACACTTTGCATTTATGTAAATATATAT

ATAATATATTATAAGTAAGCATAAATACAAGTTAATACAAGTTAATGAAAGAGCTAGCAA

AGTACTCAGATTGTAAAGGAGACATGGAAGGAAGGAAATAGAGGTTGAAGCCCTGGAGGC

CCACCTAGAGGAAGCCATATGAAACACTGGAAAAGGAGAGGTACTTTATATTCATGCTCA

CTGTTCACATAGGACACACACTACTCCACAGGTAGTAGTATGACTGAATGCAGATCAGAG

TAGGCAGGGACTCTTTCAAGTTACAAAGCTAGCCAGAAGTAAAGCAGCTAGGTTGTCAGA

AGCCTCTAATTCTGCTCATACTGCTCTCATGACACTGATAAGTCAATGCTAATGTAGCAT

AGATCATAATAGCATGCAAGACGTAGCTTTTACAATGTATAAAGTGAAGCTAACATCACT

TTTAAGTAATCAAAATGGAGTTTTTTCCTAGACTACTATTCTGATAAAGCAATAGAGCAT

TTGTCTTTCTGTAGAACCTTAAATTTTCAGTATTCATGACTTCCTGTATTTTTCATAAAT

TTCCTAACTTTTAAGACTTTCATTGAAAAAAACAACTTAGTCCCTTTATGTGTATCTTGG

GTTGAATATGTTAATTATTTCATATTGTATATCAATGCATCAAATATCATTTGTACACCT

TGATTATACACATATATAATTTTAATTTGTTAACTATACCTCAATAAATAAATAAAAACT

GAAAGTATTTAAAATTTATTTATTATTAGTTTGTATGTGTGCAGGTGTGTGTGTGTGTGT

GTGTGTGTGTGTGCGCGCGAGCGCACTTGCAGCTGATGCCATGGCATGCATGTGGATGAC

AGAGGACAGATTTGAGGAGTTGCTTCTCTTCCCTCATGGGTTCCAAAGGATCAAACTCAG

GTTGTCAGGTATGCATGACAATTACTTTTATACCTTGTTCTCTGGTCTTAAACAGAAATC

ATTTAATGTCTTAATTCTATATAAGACTTTAAACACAGTTGAAAATTCTCACTGCTTGTT

AGTCACCTTTATATGATTTTGTTGACATGGAGAAGAAGGAGCAATGGGAAGGGAAACTGT

GACAGAATGGAACATGAGGCACAGAGAGGCCTGAAGTGGGAGCTACAAGCATTGGTGTGC

TGTTTGAGATAGAAAATTAGGGAAGGGATAAGGAAGATGCCAGTGGGTTTTGGAGCTTTG

GGAAAGTAACTCTCCTCAGTTACAAGGATGATGGCTGAGCTACCCCTACTGTTTACTGTT

TTGCCTTGTTTTTAGATATTTTCTTCATTTACATTTCAGATGCTATCCCGAAAGTCCCCT

ATACCCTCCCCCCCACCCTGCTCCCCTACTTACCCACTCCCGCTTCTTGGCCCTGGCATT

CCCCTGTACTGGGGCATATAAAGTTTGCTAGACCAAGGGGCCTCTCTACCCAATGATGGC

CGACTAGGCCATCTTCTGCTACATATGCAGCTAGAGACACGAGCTCTGTGAGTACTGGTT

AGATCATATTGTTGTTCTACCTATAGGGTTTCAGACCCCTTCAGCTCCTTGGGTACTTTC

TCTAGCTCCTCCATTGGGAGCCCTGTGTTTCAGCCAATAACTGACTGTGAACATCCACTT

CTGTATTTGCCAGGCACTGGCATAGCCTTACTCGAGACAGCTATATCAGGGTCCTTTAAT

GTTCTTAATATCAGTGAAATGGCACAAAGGTGGGAACTTATGTAGGTATGGTGGCTTATG

TCTATGGTCCCAGTACTCTGGAAGCTGAAGTAGAAAGATTATGAGTTCAAGGTCCTGTCC

CAAAACAACAAACCAACAAAGCCCCTGGTATTCAGTCAGTAGGCACCCCGATAAATAAAT

AATTAAATAAAATAACAATCTTCTCGAGGTATTGACAAATAGTTTTTTTTTTTTTTGAAA

GCTATCAAAATAAGCACTTGAGGTATTTGTTAGTTATTTCTTTGTTTCTGTATCTGACTT

CTTTTTCCTAAGGACTAGGAGTTATACACTATGATTTATCAAACTTGTTAGAAGGGGAAA

TTTCAATGGGAAAAATTATTGGTGGTATTATAAACCTTGTAGCTACATATAGTATAAGCA

TAATTTGGAACACTGTATTTCAGACACTAAGGGAGGGGATTAGAAGGGAACTAATGTGTT

AAATGTCTTCTGTGTGCCAGGCACTATGCTAGACACTTTGACATGTATTACAG**GGTTTCT**

**TGTGAGTATGAAATGAGATGATGTATCCCCATTTTACAGATGAGGAGACTGACCTTGCCC**

**CAAGTCACATGGCTG**GTAAGTGGCAGAGCTGGACTAGTACCCAGACCCCAAACCCAATGC

CTTTTCTGCTGTACCGTGTTAGAGCTTGAAAAAAGAGAGGTATGAAATTCATGATCAGAA

TTGACAGCTCCAGGAATTGTTTTTCATGACAGGAGTTAGATAGTTAGAAACACACTATTA

GAATCCCTGACTCTAAGCTGCCATGTTAGATGTATCTAAAGTGTATTAAGGAAGAACAAT

GTCTCTTGTCAAGAAATGCTGGATTGAATGAACATGTGGCATCTCATGGGACTTTATATA

CCTTTGTGTTAAAGACAGTTACAATAACGTGTCTACAGAAGAGTGCTTATTAAACACTCA

GATGGCAGCATGCCTGTGCAATACCCTGGGGAGTTTAAAATTGACTTTGTAGAGGGTACA

TTAGTCCATTAAGCACCCTTCATACAAAGTCCTGTGCTCAATTCCAGAGCATAAAGTGAA

ACAATACTTTTCTTGACTTGTCTGAACATTCTTGCCTACCTGAGAATAAACTTGGAGTAA

ACTATAGCAGTACAAAGACATTTTCTTATTATTTACTGTCAGTCATTATTTTTAAGCTCC

TATACATGATTTATGGATTCCCAAAAGATAAATATGAAAATTCTAAAGTCAGGTATGGTG

GCACCTTTTTGTAACTTCCAGCCTTTAGAAGACTGCGGCAAGAAAATTGAAAGCTTAATG

CCAATCTGAGTTACATGGCAAGATCTTATCTAAAAAAACCTTACAAAATAATTTTGATAC

CTTTAGTACTTAGATGGGCTAGTAATTGGGCTTAATAAATAGTACAGGAATAAAGGAATG

TAGGGTGGTTCTTGGAAATTACCACCGAATTTGCAATGTTAGAGACTGATGCCAACATAA

ACATGTTCTCATAACTTGTTTAAAAGAAATCTGATATTTAGATTCCCTCAAACATTTTCC

CCATCATTTTAATATACTTTACGAAGATTATATTTGCATGTAACTACACTAATTCCTACT

TGCTTGAAGTAGTGAGTTGGCATTCTGGACTTGCGTTATAACAAATAGAATGAATATACA

TCTGAGGTACAATGAAGCTGCACTTCTCAGATATATCAGAGCTAATATATCCAATATATC

ATCCCCTTCAGATGCAAAGTGGCTAAGCTTTAAACCTCTTTGACAGATTTAAAGAATGCC

CTGTTTGCATTGCCCTTGTCCCTCATGTTGCCAAGCCTGGTTTTCCAAGGGCTTCTGTAG

CTTCTGCAGACAGACAAAAGGTCTTTTGTTGTTGCTTTGCTTTGCTCTGTTTTTAATATG

CTGTTTCTGGTATGCATTTTATCTGTGGTGTGTCTCTTTTCCTCTTCCCTTAAAAAGATG

AAAAGAATGCCCTTAAACCAGCTTTCAAACATTCTGTTCTCACTTATATTATAGAAAATA

GTGGCTGGGCGTGGTAGCGCACGCCTTTAATCCCAGCACTTGGGAGGCAGAGGCAGGCAG

ATTTCTGAGTTTGAGGCCAGCCTGGTCTACAAAATGAGTTCCAGGTCAGCCAGGGCTATA

CAGAGAAACCCTGTCTCGAAAAACAAAAAAACAAAAACAAAAACAAAAACAAAGGAAGGG

AGAGAGAGAAGGAGAGAGAGGGAGAGAGAGAGGGAGAGAGAGAGAGAAAGAAAAAAGAAA

GAAAGAAAGAAAGAAAGAAAGAAAGAAAGAAAGAAAGAAAGAAAGAAAGAAAGAAAGAAA

GAAAAGAAAGAAAATAGTGAATGTGTACAACAGGGGAGAGAACTGTGAAACCATAACCAT

TGCTTCCTCCCTTTCCTGCCTGCCTCTCCCTCTTAGAAAAGCACTTTCTATATTTAGGAC

ATGGCAGCAACAGAAGAACCCCTCACACGCTGCACATTTTTAATATTGTTTTTACACATG

TTTGTGAGCAGTTTCAAAGAAGGGTTGGAAGCAGGTGTGAATTCAGGTTGCCAGCAAGAT

TTGAGGGCAGGGGTGTGCAAACTTGGTCACTGGCTATTGTGTGGACTTCTGGACAGCCTC

CAGCTGGGACATTGTTTCAGCATTTGTTAACTTACAGCCTTCTCTGGATGTGGGTAAAGA

AAGGTGGGAGGACCTTAGGCCAGGTTCCCACTGTTCTCTCTTATTGCACGATGTTAGTTT

TTATATGTCATTTTCTCCTTAAATATTTACAATTGCACTTCTAATGAGATGTCCTTGTGT

ATAGTGTTTGCAAGTGTCATGAAAATAGAGGACTTTACTCAAGATGCTGATTTAAGAACT

TTATTGCTTTTTCTTCCTGTTCTTTCTGTTACCGTTAATGAATATACAGCCAATTAGATT

CCTTCCCTTGCCTTAGCCTTTTTCTTTTTTTCTTCTTATTTTGGTATTGGGAGGCTTTCT

ATATCTGTTTTAGGTGCAGGCAAATGGAAGTCCAAGCTGAAGCTTCCAGAGGATGGCTAT

GCCTAGAGTATTCGTGTTGCCTTTACTGACTTGCTAGAGCCAGTTGTTAAGTTTCAGAAC

TTTTGTGAGATGGTTGTGGAACTAGCTGTTATTATCAATTATCAGTGTATGTATATGTTC

ACATACTTAAATAGATTTCATTGAAAGCAGCAATAGTCCTCAAATACTTATAATTTCGAA

TAATCTTCAGGTCATTTCCTTTTGTGAGCACATCTCTTCTCAGCTTGACACTGAGTCAGG

TCATATTGATGTATTTGAACTT

**GGTGGAATTTGCTACAGTGGAAGTG[41bp_insertion_between_SDL2_primer_and_exon_loxP_site]**TTGTCACTGGGGAAGGCATCAGAGTGCAGAGCAGGGATCCTCACTCTTGTCTCCAAAGAACAGCTCCTTATAATTACTAGCACACTGCTGATACAGGCTAAATGAACTGTAGTCAAAAACCCTATTGCTAAAAGAGGTCTAAAATCAGCTAAAACATTTTCTAAAATGTCGGGAACAGGCTAGAGTATTTGTAGCTTTGTTCTAATTTGTCAAATAAAATTCTTCATGTTTAAGATCCTTATACTACTAGGAACAATAATTTTTACCTCTACTAAACAATCTTTACTGACATGTACAAGGTATACTATTTCTAGGTTAATTTTACATAAAACATTAGTGAGTGGATTGGTGATATAAGCTTGTAATACCTTATTCCATTGCAG**CTGCCACAGGTGACATGCCAACTTACCAGATCCGAGCTCCTACTACTGCTTTGCCACAAGGTGTGGTGATGGCTGCCTCACCAGGAAGCCTGCACAGTCCCCAGCAACTAGCAGAAGAAGCAACTCGCAAGCGGGAGCTGAGGCTGATGAAAAACAG**GTAAAGTACTGGGTAATCTAGAGCCACTGTAGGAAACATTTGCTACTGCGGAGTTCTTAATGTACATTTGCAAAACTGTTTCTCAGATTAAGTGTTCTGTGGCTTCGTGTGTGTGCTTATGCTCTGCCCATGATGATAGTTTGTCTTCTGGTGAGTTCCTGGTCATTGATGCTTATGAAGATAAGCAGTAGATAT[**~120_bp_insertion**_**from_cloning_contains_loxP_site**]GTTTCTTCATGTTGTGAGGAATATCAAAAGATATGTAATCTTTAAAAACAGTATTACTATAATATATATGTATATATGTTAAAAATAGTTCACTAATGAAGAATTTTTTAAAAGATTTATTTATTTTATGTATATGAGTATACCATTGCTCTCTTCAGACACACCAGAAGAGTGCATCAGATCCCATTACAGATAGTTGTGAGCCACCATGTGGTTGCCGGGAATTGGCTCAGGACCTCTGGAAGAGCAGTCGGTGATCTTAACTGCTAAGCCATCTCTCTAGCCCTATTATATTTTTTATGATAAATTTGTTTTATATATTTTTGTGAAATAGTAT**CCTGGTAAGTACATTTTACCCCT**

TAAATTGGTATTATAGATAAAACCTGGGACCTAGGGCATGCTAGGCAAACATTGAGATAC

ATTTCTGTCCCCAATTTTTTAGCTTCTTTATTAGCAAAATAAATTTAAGTATTAAAGCAT

TGTTTTATATTTTATCATTCTAGCTACTTTGTATTGTTTTTACACCAGCACAATAAAGTA

CACGTTCTTTTGACATTCTAGTCATGTATAAATAAAAAAATAATGGAACCTCCTCATGCC

CCTGCCCTCTAGCTAATGGCTAATGCTGTAGTGGGAGCGCTTGCAGTCCTCTCTCATCTC

CCACTTGTGTTCTGAGTCTGTCTCTTGAGCACCTCCATTTCTCTCCCTGCTTAGGTACCA

GCCTGCCATGTTAAAAACACACTTAAGTAATAACAGTTTATTTACTCGAATTGCATATTA

ATTCTTTAGTTATTATGGAGAATGGTACAACTCAGTTTGATTTTCAAGATGAACAGAGTT

GCCTTAACTGTTTCCCAGTATATTTTGTCACCTATTCTTTAGTGTTAGATTATATCCTAA

TTGCAAGAAAATAAAGCACATTAGGTGGGGTCAGCTGCTTTGCTGGTCTGTAGAACAGAA

GTAGACTGGTCTTTGACAGTCTTAATAGATGTTTCCAACCTAGTTAATCTACAGATTG

TGGTAGTGGAATCCCTGAAACTCAGTACTGAGATGATCACTGCCCTTAGGTCTTGTCTTC

ACTGCATTTGTGTTTCTTTAAATGACAGTTTGGGAGCTAAGAGAGATAACTCAGCAGTTA

AGAGCATCTAGTGTTCCTCAGAGAACCTTAGTTCTGTTCCCAGCAACTATATCTGGTGAC

TCACAGTACCTTGTAACTCCAAGCTCCCTTTTCTAGCATTCAAAGGGACCTATACAGGCA

TGTGCTTGCGCGCGCGCGCGCGCGCGCGCGCACACACACACACACACACACACACACACA

CACACACACACACACAGATAAAAATAAGGATTGGAGAGATGGCTTCACACACACACACAC

ACACACACACACAGAGAGAGAGAGAGAGAGAGAGAGAGAGAGAGAGAGAGAGAAATAAGG

ATTGGAGAGATGGCTTAGCAGTTAAGAGCACTTACTGTTCTTGCAGAAGACCTGGGTTTG

ATTCCCAACTCTCACATGGTGGTTCATAACTTCCTGCAACTCCATTTCTCAGGTGATTGG

TACCCTGTTCTTGCCTCCATAGGCACCAGGCATGCACATGTTGCACATACATAAATTCTT

GCAAAACACCTATACACATAAAAATAAGACTGAGTGTGGTGATATACACCTTTTATTTCA

GCACTTTGGAGGAAGAGGCAGGTGGATATAATGAGTTTGAGGCCAGCCTGGTCTACATAA

TACAGTTCTAGGCTACCCAGGGTGACAAAAACAAACAAACAACTATAACAAAAACTTTTA

TAAGAAACAAAAATAAATCTTTTTAAAAAAAGATTCAATTTCATATACCGTGTAACTCCT

TAAGTTGGCTACATTTGCCAAATTTATTGCTTGCCTAGATCAGTAGATGGTGAATACACA

TACTTTAGAAGTAGGATGATAAAGATAGAAACATTTATCAGTGTTCTGTCCCAGGTAATA

TTTTTTTATCAGCAGTGTTGTTGCTTTAAAGATCCAGAAGGAATGGCTGGAGAGGTGGCT

TAGAGGTAAAGAGCACTTGCTGCTCTTCAGAAAGAACCAAGTTTGGTTTCCAGCATATAG

GTCAGATTGGGACACACCTATATTTAAATTATGTAATTAATAATTACTGGCATATATGGG

AAATCTTTCTGGCAGGTACAGAGGTATGTACAGCCGGAACAAAGGTCTCTGGTGTCTCCA

GGGACCGGGCTTCTTGCTGCCGCATCTTCAGCATGCACATTTTTGTCATCAGGACCACAG

GATGTCTGCTGTTGATGATGACAGGAAGAGGGGAGGAGGGCTGTGGTCAAAGAGTGCAGA

TAACTCAGTCTGTTTCTCCAGAAGACGTTCCTGTCTGCTTTTACCTACATTCCATTGGCT

AGAAATAGGGCCCCACTATAAGGGACTCTGAGGAGAATTATTTAGCCTGCTAGTGTCAGA

CTAGAGATAGCTAGAGAAAAGCAGCAGCATATGGCTCTTGGGTGACTCAGCATTAGCTTA

ACAAATTTCAAGCACTTGTTCCTCCAGTTTCCTTAAGTCTTACCCAATTCTGTGAGTACA

GAGTAATTCAAGCCTCACTCTGGAAATGAGGACCTGGAGTTGGAATGGTTAAGTGGCAAG

TTGGCCAAGATCTTAGAGCCAGGGGACCAGAAGTAGGCTATGCTGCCTAGCAGTTCTTTC

TTTCAGAGCCTTCCTCTGTCCTTATAGCAAGGTCACCTCAAAGGATCTTGATGTAAAGTC

TGGACTTCATGGAGAAGATGCAGGCTTTACAGTTAGCAAGAGCAAGATCAGGTATCAGGT

GACATTGATAACAGATAATTATATCCCTATCACACTCGTCAGCAGTAGCTTGGATTCCTT

GTAGCCTGGTCATCATTTCCTGTTTAGCTTGACATTGGCTTTGAGATGGTGTGGCCCAGG

CTGGCCTCAGACATGTGCTTCTCTTGCCTGCCTTCACCTTCTGAGTGTTGCTGGTACAGG

TGTGTAGCACACCTGGCTCTTCACCCTTTTCCTGCAGTTGCCATATGATTTCCTTTCCAT

GCTGAGGATGTATTTGGTCAAACACAAGCCTGCAAACTGAGCTACCTATTAATAGAAGAA

ACAGGTGTTCTCCACAGCTGAGAATACTTAGGAATACTTGTGTGATCACTAAAGCTTTGT

GCTTGGGACATTTTGACTCCAGTCACATCTCACACACAGTGTCTGATAGAGAGTTTGGAT

ATTCACAGTAAGTAAGAGGTTAAAAGCTGACCACCTCACAACACTGTATTTAAATTTTCA

TTTAAAATTTGTATGGGAAGACTGTCTTCCTTTTATATTTAGAGTTTAACATATTTAATG

ATGAAAACATAGGTACTTATAAATACATTTAAATTCCCTGACTGGCAGCTATGCGGGGAG

TATGCTGACACGCCCCACTGTGCACCATTTGGATTACTGTAACCCCTGCTTTACCAAGCT

CCATTTACTTTGACATGTTTTAGTTTTTTTTCACTGCTCAGTTGTGCAGCCCTTGGTGGG

TTCACAGGGTGAGTATAGCACTCTTGGCTAGGAACTAGGGCTTGTTCTTTTTCCTGCAGC

CCATCAAGATTTCAACATTCATTTTCTGCTGTCTTGCTTTGCATGGGAAAGAGACATCCA

TTCCATGAGAGCCAATTCTTCTGAATTGTGAAAATATAGCAGTTGGCCATGTCTGTTGTA

TGTGTCACAGATGGGGGATGGCATTGCTAGCTGACATTGCAGTGACTGGTATCTACCTTT

GTGAATTCAGTTGAGTGACAGTGACTGGCAATTTCTGAGAACTAAGTATTCCCAAATAAA

GCTTTATCATAAAGCCTCTCCTTAGAGCAGTTAAGTGAAGTAGCCTCTATCCTGTCCCAC

TTTTAAAAACAGGAGAAAGAACATGGGGACAGTGTTAGTTATTGTTTACTGGAATCACAC

ATAAAAGTGACTAACTTATCTTTGTTAGTTTCAGTTATTTGGGTGCTTGTTGGTCTGTCT

TTTCACTTTGGTTGCGTAATGTGATGTGAAATGTGTTCAGTTAGCTAGTAACTGCCATTA

TATTTGTCTGTCTGTTTATTTATGATAGGCTCCCAAATACCTCTGACTAGCCTTGTGCTT

GTAAAGTAACTGAGGCTGGCCTGGACCATTTTTTTAAATGTGTATATGTATTTGGCCTGA

GTATATATCTGTGTAACACATGTATGCCTAGTGGCCACCCTGGAACTGGAGTTATAGACA

GTCGTGAGCCAGCATGTGGGTGCTTGGGATTGAATCTTCCAGGTCCTCTGGGAAAGCACC

CAGTGTTTGTAATCACTGATCTCTCCAGCTCTTGGTTTTGTATTTGTGGTTCTGCTGTCT

CTACCTCCCAAGTGCTAGGTCTACAGACATGTACCACCATGCCAGCTTCCTTTTCTGTAT

TTGTAATTGTGGTATTCTAACACTTGTCAGCTTAAATTGTCTGCGTTGTTAAGTTTTGTG

TCATGTTATGCCATTCTTTAATTTTTACAAGCAGTGCCCATTTTCCCTTAGTGCATAAAC

ATCCATGTGTTTTATTTATTGTTATAATTTATCTATATTGTGTTTTCTATTCAGGTTGTT

TTTGAAAACCGTGACTTAGAGATTTAGTCTCAAAGTATACAACTTTTCGATATTGGCATT

TGAGACTTCATAGGATTTTTAAACTCATTTACATCAAGTGACATTTTCTCGTTGTGATGA

GCATGTATGTTTTTTGACAACCAAAAATTAACTGAGAGGGCTGAAAGGATCTGTGAATGT

CTTAGTAGGGATTCTGCTGGGAGTGTTGTCCTTTCATTGTTGTTTATTGTACACAGAGGG

TCTTTCTTGCCGTGGCATGGGTGGCCCAAGAGAACACTGTTTGGGAAAAGCTACCAGTGC

TCCTCATTTGGTCTGTTGTTCTCTACAAAGCATCTGCTTTGCTCTCTTTGTTGTGTAGCT

TTGTTTCCTCAGGTTGGTTGGCTGTTGACTGGGTAGAAGGGGGGGCAGATGTTCACGGAG

CCATTCTGGATTGTGCTGGGAGGTTGTTCCTGTTGTCATTTGCCTTGTGTTGGTTCCAG**G**

**GAAGCTGCCCGGGAGTGTCGCAGGAAGAAGAAAGAATATGTCAAATGTCTTGAAAATCGT**

**GTGGCTGTGCTTGAAAATCAAAACAAGACCCTCATTGAGGAACTCAAGGCCCTCAAAGAC**

**CTTTATTGCCATAAAGCAGAGTAACTGTGTTTGATTTGGACCTTGTTGACTGTGAACTCT**

**AATCGGGGCAGGCGATGCAGCATCCTCATAATGGCCATGTGGACTTGTAGATGGGTCTCT**

**TAACCCTTGCTTAAGAATACAGTCTGCTGTAGAGTGTGAATTGGGAATACTGTTCCATGG**

**GTTGGAATGCAGCTCCCCTCACATTACCAAGCTTGCTCTATTGCCAATAGCATGCAACAT**

**ATGTTTTGTTTGCCCTTCTGCTTCTACTTTTTTCAGGGAAGCTGCTAAAGAATGTCGACG**

**TCGAAAGAAAGAGTATGTGAAGTGTCTTGAGAGTCGAGTCGCAGTGCTGGAAGTTCAGAA**

**CAAGAAGCTTATAGAGGAGCTTGAAACTTTGAAAGACATTTGCTCTCCCAAAACAGATTA**

**GTAGAAATATTTAACTATGAACTGATTACAGCATGTACAGTTGCTTTTGAATGCAATACA**

**ATATATAGCCGGCAAGAATTATGGCTTTTTCCTTTGTATCATTCATCTAACTTTCTAAAA**

**CTAACATTCCTAAGATGCTTTGTTGTATTTAATTTGCTCTTACCTCTAAGGTCAATTTTT**

**TAGAAGAGACAAACTGTAAAAAATGTATGTAACAAATTCTTAAAATGAAGTATTTGTAAG**

**ACTTGTTCCAGTGCAACATATTTACAGTTCCCAGTCTCTCTGTCATGAATAGTGTCCTAT**

**GCAATAAAAATTTTGCAGGTTTTAAGAATCATTTTAGGAAAGGGTGATCAAAGGCAGTGC**

**ATCTCTCCAGTAGTAAGATAAAATCAACCCATAGAGATACCTCAGGAAAGAATGAAAGGA**

**AGTGTATCCTGATGACATGACTACGTGAGAATAGCCTACAAATGAATTTATGCATTTATA**

**GATTTTTATAATCGTCACTTTGTAAAGAAAGTATTGTATTGCTGTCCTTGGGTGCCACAG**

**TTGAAGACAGTTTTAAATAGAACCATGTTGGTTGCTCTTTGTACTATTTGGTATTTATTT**

**AAGTATCTGAGCATTTACTACAGCTTCCTACTATGTATGTAGTATGTGAATTTCTACAAA**

**AGTTTGTGCTGCTTTGCTGTTATTTAATGAAAGAGACAACATATTTTCATTATCTGGAAT**

**GAGTTCCACAAGTATGAATTTATTGCTACACTGGATCAGCAGCCTTGCAAATACTGGGCC**

**ATTTCATTAGAGGACAACTGCAGGGCTCTAGGAGCAGAGTTCAGTGTGGAGCACTTGCCT**

**GGCATGCTACATGTTCAGTTGAAAGGGAAGACCTCAAGCTCTGCAAATGGAATGGGGTCC**

**AGGGGAAGAGGTTAGAGGTTAGCCTTTGTGCTGTACTAGGCTTCTTGCTGATCGTCTGGA**

**GAGTTTCTGCTGATGACCCTCCATTGTGAATTCTTGCAACCTCAGGAATGTTAACGTTTA**

**AAAAACAACCCAAGATGTCATTTTTGATTTTACAACTTGGATCAATTTTGTTTTGCTCTT**

**TGGAATATAGCTGTGTACATTTGTCACGTAGGTTTAGGCTGGCCTTAAACTCACAGTTCT**

**CTTGCCTCAGCCTTCTGAGTGCTTGGATTACGGATGTGGGCCAGAATATCCAGTTTGATC**

**AAGTATTCTTTTATAAAATATTACTTTCTTTTTAATTGCCTGTGTGTATGTCTATGTGTA**

**TGTGAATGTGGGTTCCCAAGAAGGCCAAAAGCTCCCCTGGAGCTAGAGTTATAGATAGTT**

**GTGAGCCTTCCAACATGGGTGTTGGGAACTGAACTCAGATATTCTGCAAGAGCAGCACAT**

**GTTCTAAATGACGGAGCCATCTCTCCAGGTCCATGATAAAATATCCTTATGCCAGAACAT**

**TCAAATGAAATTCATTACAATTGTGATAGCTTTGTTACTAGTAGCGTCCATAGTTGTTTT**

**TATAAGGAAAAGACTCAAGGTATAAGCTCGGATCCCTAACC**CTGTGCTATGTCATCTTTC

TCTCACCAGCAGGTACGGATTAGACAGTGGGCTATAGTCGCTGCATTGCCTATGGCTGAT

CTCTGATTTGTTAAGAACCTG Protein Sequence of the Cre Recombinase deleted exon:

MPTYQIRAPTTALPQGVVMAASPGSLHSPQQLAEEATRKRELRLMKN

This exon is present in all 11 isoforms on UniProt.

Full protein sequence (isoform 1; P27699-1) with deleted exon in bold and protein’s bZIP DNA binding domain in red:

Residues 263-309 are deleted by Cre Recombinase activity

MSKCGRKKYMRTNVRQMTMETVESQQDRSVTRSVAEHSSAHMQTGQISVPTLAQVSVAGSGTGRGSPAVTLVQLPSGQTVQVQGVIQTPHPSVIQSPQIQTVQVATIAETDDSADSEVIDSHKRREILSRRPSYRKILNELSSDVPGIPKIEEEKSEEEGTPPNIATMAVPTSIYQTSTGQYIAIAQGGTIQISNPGSDGVQGLQALTMTNSGAPPPGATIVQYAAQSADGTQQFFVPGSQVVVQDEETDLAPSHMAAATGD**MPTYQIRAPTTALPQGVVMAASPGSLHSPQQLAEEATRKRELRLMKN**REAAKECRRRKKEYVKCLESRVAVLEVQNKKLIEELETLKDICSPKTD
