# Supplementary material for: The cAMP responsive element modulator (CREM) transcription factor influences susceptibility to undernutrition and infection
Source: mBio. 2025 Jun 27;16(8):e01390-25. doi: 10.1128/mbio.01390-25 (PMC12345263; doi:10.1128/mbio.01390-25)
Supplement: Legends — Supplemental figure legends. [file mbio.01390-25-s0008.docx]

**Supplemental Figure Legends**

**Supplemental Figure 1. Distribution of WAZ across cohort children is not different by amebiasis history or sex**. Weight-for-age Z-score distributions at one year of age stratified by A) amebiasis history and B) sex. Stratified data are also presented by child genotype at both rs2148483 and rs58000832. D- and P-values represent Kolmogorov-Smirnov test results for the respective graphs on which they are displayed. C-D) Distributions of WAZ by genotype at 1 year of age stratified by cohort. Unadjusted median WAZ values are indicated within graph.

**Supplemental Figure 2. *CREM* rs2148483 variants do not significantly affect *CREM* locus expression in duodenum**. A) Normalized counts of all *CREM* transcripts analyzed from 199 human duodenal biopsy transcriptomes (adjusted p-value from DESeq2 result = 0.60). B) *CREM* isoforms constituting at least 1% of all *CREM* transcripts expressed in duodenal biopsy transcriptomes. No significance differences (p < 0.05) were discovered between homozygous reference (G) allele to heterozygous groups by multiple Mann-Whitney tests with Benjamini-Hochberg correction. Asterisk denotes the ICERI-gamma encoding transcript, the only ICER isoform found to constitute at least 1% of all CREM transcripts. Normalized counts of C) *CCNY* and D) *CUL2* transcripts analyzed from duodenal biopsy transcriptomes (adjusted p-values from DESeq2 = 0.63 and 0.26, respectively).

**Supplemental Figure 3. Murine gut flora is not altered by induced *Crem* deletion.** A) Alpha- and B) Beta-diversity of the gut microbiome. C) The top 10 most prevalent microbial genera by relative abundance in *Crem^fl/fl^* and *Crem^fl/fl CRE-ER^* mice measured pre-tamoxifen and 4 weeks post-tamoxifen. Bars represent S.D.

**Supplemental Figure 4. *Crem* deletion does not significantly affect respiration, activity, food intake, or intestinal permeability**. A) Key metrics of respiration and activity measured by Oxymax CLAMS metabolic cages are presented as change per mouse two weeks post-tamoxifen compared to pre-tamoxifen. Data are separated into measurements taken during light and dark vivarium lighting periods. B) Daily food intake normalized to total mouse weight. C) FITC-dextran detection in mouse serum to determine intestinal barrier permeability. RER = respiratory exchange ratio. D) Water mass measured one to three-weeks post-tamoxifen treatment.

**Supplemental Figure 5. *Crem* deletion does not significantly alter key metabolic hormone levels**. Effect of *Crem* deletion two weeks post-tamoxifen on A) insulin and B) glucose tolerance tests presented as time course (left) and area under the curve (right) from two independent experiments. C) Leptin and D) adiponectin concentrations in serum measured by ELISA. Bars in A and B represent S.D.
