# Supplementary material for: The cAMP responsive element modulator (CREM) transcription factor influences susceptibility to undernutrition and infection
Source: mBio. 2025 Jun 27;16(8):e01390-25. doi: 10.1128/mbio.01390-25 (PMC12345263; doi:10.1128/mbio.01390-25)
Supplement: Table S1 — Characteristics of individuals providing small intestinal biopsies for transcriptomics. [file mbio.01390-25-s0009.docx]

**Table S1.** Characteristics of individuals providing small intestinal biopsies for transcriptomics. ‘N (rs2148483 AA)’ represents the number of individuals homozygous for the alternate allele (A) at rs2148483. These individuals were excluded from further analysis, including characteristics quantified in this table. WAZ = Weight-for-age Z-score; BAZ = Body mass index-for-age Z-score; BMI = Body mass index.

|  | **American Children** | **Bangladeshi Children** | **Bangladeshi Adults** |
| --- | --- | --- | --- |
| **N** | 81 | 37 | 81 |
| **N (rs2148483 AA)** | 4 | 2 | 4 |
| **Female (%)** | 37.7% | 54.3% | 63.6% |
| **Median Nutrition Metric (SD)** | 0.35 (1.5) | -2.14 (0.8) | 17.8 (3.8) |
| **Nutrition Metric Used** | WAZ or BAZ | WAZ | BMI |
| **Median Age in Years (SD)** | 11.4 (4.5) | 1.5 (0.18) | 24 (7.7) |
